# Harnessing the power of bee venom for therapeutic and regenerative medical applications: an updated review

**DOI:** 10.3389/fphar.2024.1412245

**Published:** 2024-07-18

**Authors:** Kadry M. Sadek, Naira A. Shib, Ehab S. Taher, Fatema Rashed, Mustafa Shukry, Gamal A. Atia, Noha Taymour, Mohammad El-Nablaway, Ateya M. Ibrahim, Mahmoud M. Ramadan, Afaf Abdelkader, Mohamed Abdo, Ilinca Imbrea, Elena Pet, Lashin S. Ali, Ahmed Abdeen

**Affiliations:** ^1^ Department of Biochemistry, Faculty of Veterinary Medicine, Damanhour University, Damanhour, Egypt; ^2^ Department of Basic Medical and Dental Sciences, Faculty of Dentistry, Zarqa University, Zarqa, Jordan; ^3^ Department of Physiology, Faculty of Veterinary Medicine, Kafrelsheikh University, Kafrelsheikh, Egypt; ^4^ Department of Oral Medicine, Periodontology, and Diagnosis, Faculty of Dentistry, Suez Canal University, Ismailia, Egypt; ^5^ Department of Substitutive Dental Sciences, College of Dentistry, Imam Abdulrahman Bin Faisal University, Dammam, Saudi Arabia; ^6^ Department of Basic Medical Sciences, College of Medicine, AlMaarefa University, Riyadh, Saudi Arabia; ^7^ Department of Medical Biochemistry, Faculty of Medicine, Mansoura University, Mansoura, Egypt; ^8^ Department of Administration and Nursing Education, College of Nursing, Prince Sattam Bin Abdulaziz University, Al-Kharj, Saudi Arabia; ^9^ Department of Family and Community Health Nursing, Faculty of Nursing, Port Said University, Port Said, Egypt; ^10^ Department of Clinical Sciences, College of Medicine, University of Sharjah, Sharjah, United Arab Emirates; ^11^ Department of Forensic Medicine and Clinical Toxicology, Faculty of Medicine, Benha University, Benha, Egypt; ^12^ Department of Animal Histology and Anatomy, School of Veterinary Medicine, Badr University in Cairo (BUC), Badr City, Egypt; ^13^ Department of Anatomy and Embryology, Faculty Veterinary Medicine, University of Sadat City, Sadat City, Egypt; ^14^ Department of Forestry, Faculty of Engineering and Applied Technologies, University of Life Sciences “King Mihai I” from Timisoara, Timisoara, Romania; ^15^ Department of Management and Rural Development, Faculty of Management and Rural Tourism, University of Life Sciences “King Mihai I” from Timisoara, Timisoara, Romania; ^16^ Department of Basic Medical Sciences, Faculty of Dentistry, Al-Ahliyya Amman University, Amman, Jordan; ^17^ Department of Forensic Medicine and Toxicology, Faculty of Veterinary Medicine, Benha University, Toukh, Egypt

**Keywords:** apitherapy, anti-inflammation, anticancer, antimicrobial, neuroprotection, wound healing

## Abstract

Honeybees have been helpful insects since ancient centuries, and this benefit is not limited to being a honey producer only. After the bee stings a person, pain, and swelling occur in this place, due to the effects of bee venom (BV). This is not a poison in the total sense of the word because it has many benefits, and this is due to its composition being rich in proteins, peptides, enzymes, and other types of molecules in low concentrations that show promise in the treatment of numerous diseases and conditions. BV has also demonstrated positive effects against various cancers, antimicrobial activity, and wound healing versus the human immunodeficiency virus (HIV). Even though topical BV therapy is used to varying degrees among countries, localized swelling or itching are common side effects that may occur in some patients. This review provides an in-depth analysis of the complex chemical composition of BV, highlighting the diverse range of bioactive compounds and their therapeutic applications, which extend beyond the well-known anti-inflammatory and pain-relieving effects, showcasing the versatility of BV in modern medicine. A specific search strategy was followed across various databases; Web of sciences, Scopus, Medline, and Google Scholar including *in vitro* and *in vivo* clinical studies.to outline an overview of BV composition, methods to use, preparation requirements, and Individual consumption contraindications. Furthermore, this review addresses safety concerns and emerging approaches, such as the use of nanoparticles, to mitigate adverse effects, demonstrating a balanced and holistic perspective. Importantly, the review also incorporates historical context and traditional uses, as well as a unique focus on veterinary applications, setting it apart from previous works and providing a valuable resource for researchers and practitioners in the field.

## 1 Introduction

Bees are commercially beneficial insects that have been around since the Cretaceous age of the Mesozoic Era. They also help fertilize many different crops. Bees are helpful, but their capacity to administer excruciating and poisonous stings constitutes a risk. Thankfully, most honeybees are not hostile to people and only resort to violence if they perceive danger (Pucca et al., 2019). *Apis mellifera* is the most often used honeybee species for agricultural pollination globally. All bee products, particularly venom, and honey, have been used for centuries, and their medicinal properties have been described in holy writings such as the Bible and the Quran (Ali, 2024; Dinu et al., 2024). Bee venom (BV) treatment involves injecting honeybee venom into the human body to cure various ailments. For over 5,000 years, this technique has been used in complementary therapies. It consists of either an indirect implementation, like isolating BV via stimulating electricity and then administering it into the body, or an immediate administration, such as bee stings (Wehbe et al., 2019). Applying BV in medicine stemmed from discovering that beekeepers rarely experience rheumatism or joint discomfort (Ullah et al., 2023). Across different cultures, BV has been employed in ethnomedicine as a natural remedy for a wide range of health conditions. Traditional practices have incorporated BV for its anti-inflammatory effects, pain relief properties, and immune system stimulation. In ethnomedicinal practices, BV has been used to alleviate symptoms of arthritis, rheumatism, and inflammatory conditions. Additionally, BV has been applied for its potential in wound healing, skin conditions, and even as an antimicrobial agent (Bava et al., 2023). BV is a colorless, scentless liquid of numerous molecules with a slightly acidic pH (4.5–5.5), which bees utilize as protection from enemies. A single drop of BV comprises 88% water and only 0.1 g of dry venom (Bellik, 2015). BV consists of several compounds and a significant quantity of water (Farook et al.; Essam Elenany, 2024). BV content has previously been identified via omics and fractionation techniques (Pucca et al., 2019). BV is a biotoxin or api-toxin produced by a gland in the bee’s abdomen cavity. It comprises a complicated blend of many physiologically active peptides-incredibly intricate peptide combinations. Melittin, adolapin, apamin, and MCD-peptide are only a few examples. It also has powerful enzymes, like phospholipase A2 (PLA2), hyaluronidase, acid phosphodiesterase, -D-glucosidase, and lysophospholipase are enzymes, and in addition to low-molecular-weight molecules naturally occurring amines (e.g., histamine and epinephrine) and micronutrients (Moreno and Giralt, 2015). Apitoxin formulation is influenced by bee age, where they live, seasonal shifts, and social standing. Melittin, apamin, hyaluronidase, and PLA2 levels are particularly sensitive to change.

Furthermore, the procedures for collecting BV influence the volatile component composition. Electricity that is used to stimulate histamine can vanish during BV extraction (Schmidt, 2018). Many investigations have examined the curative effects of these substances in managing numerous illnesses and conditions (Wehbe et al., 2019). BV possesses anticancer, antimicrobial, and antiviral activities (Gajski et al., 2024).

This article offers an updated and in-depth review of the intricate chemical profile of BV, emphasizing the wide variety of bioactive compounds and their therapeutic potential and clinical applications against various illness. This highlights the adaptability of BV in contemporary medicine.

## 2 Sources of bee venom

Female honeybees generate venom from a gland situated in their abdomen cavity (Ali, 2024). The gland is attached to a container capsule. Apis social insects rely heavily on their seminiferous system for protection. Bees sting near apiaries to defend their colonies (Piek, 2013). The queen uses stings to eliminate competitors (Portelli, 2020). When many queens are born simultaneously, they escape with a particular number of bees, murder the unborn queens in their cell, or participate in a dying battle. Each hive may only have one queen. BV has the maximum protein concentration in the initial 1–3 days of life, then declines following 7 days. This is critical for killing competitors in the fight for beehive leadership. Honeybee venom’s protein concentration decreases when the gland degenerates in subsequent days. Female honeybees do not produce detectable venom at the moment of emergence (Bava et al., 2023). Instead, it swiftly climbs during the next 2 days, stays steady for 14 days, and then decreases. Older honeybees generate less venom than younger bees. The venom’s makeup varies with age. Melittin is secreted as a dormant component becomes active during growth and passes into the guardian stage around day 20 of age (Bava et al., 2023). Honeybees withdraw their venom sac and pointed stinger from their abdomen when stinging.

In contrast to similar insects, they are limited to stinging once before dying (Elieh Ali Komi et al., 2018). When a bee strikes a human or an animal, the stinger remains attached under the outer layer of skin, and the honey bee passes away by pulling out its guts, muscles, and nerve center to remove itself. Many of the bees’ bodies are lost, leading to death. The stinger’s sharp tip has microscopic hooks that prevent it from being eliminated without causing damage. Once lodged, the venom is pushed into the wound using a separate piston mechanism (Lee et al., 2020). The stinger integrates into the tissue, discharging the components of the venom chamber within a few minutes (Elieh Ali Komi et al., 2018). BV sends an alarm pheromone that stimulates other honeybees to protect the beehive (Elieh Ali Komi et al., 2018). BV can induce localized inflammatory reactions, such as discomfort, warmth, and irritation, as well as systemically triggered allergic responses that can lead to anaphylactic shock and even death (Annila et al., 1996).

BV can be collected using various techniques, with the most common method being the electric shock extraction process. This method involves exposing bees to a low-voltage electric current (20–30 V), which induces them to release venom that is then collected on a glass plate. The bees are not harmed during this process, making it safe for them. Another traditional method involves surgically removing the venom gland from bees or squeezing individual bees until a drop of venom is extracted from the stinger tip. However, this method is less common compared to the electric shock technique. The venom collected through the electric shock process is considered effective and is widely used in clinical applications. The final product has varying properties depending on the extraction process used, with the most effective venom being that which is collected under water to prevent the evaporation of the highly volatile components (Otręba et al., 2021).

After collection, BV undergoes processing to extract its active components for medical use. Techniques such as chromatographic separation and molecular genetic methods are employed to isolate individual BV active ingredients for specialized medical purposes. These processes allow for the extraction of specific compounds like melittin and apamin, which possess unique therapeutic properties (Pucca et al., 2019; Bava et al., 2023).

To ensure the reproducibility and safety of BV-based therapies, it is crucial to accurately identify and measure the components of BV. Standardization of BV is necessary to guarantee the consistency of the final product and its therapeutic efficacy. Factors such as the bee species, season, and geographical location can influence the composition of BV. Therefore, quality control measures, including analysis of moisture content, protein profile, and cytotoxicity, are essential during the processing and extraction stages (El Mehdi et al., 2022).

## 3 Composition of bee venom

BV is a complex mixture composed primarily of water (88%) and various peptides, enzymes, and other compounds. The main components of BV including (1) peptides; melittin (50%–60% of dry venom), a small peptide with 26 amino acids that causes pore formation in membranes; apamin (2%–3% of dry venom), a neurotoxic peptide mast cell degranulating (MCD) peptide; and adolapin. (2) Enzymes; phospholipase A2 (10%–12% of dry venom) destroys phospholipids in cell membranes, hyaluronidase, phosphatase, α-glucosidase. (3) Biogenic amines; histamine, epinephrine (adrenaline). (4) Other compounds; lipids, carbohydrates, free amino acids, and minerals.

The composition of BV can vary depending on factors like bee species, season, and geographical location. However, melittin and phospholipase A2 are consistently the most abundant components, making up over 80% of dry BV. The complex chemical nature of BV contributes to its wide range of biological activities and therapeutic potential (Sharaf et al., 2024a). Hymenoptera venom includes several active elements, small-molecular-weight compounds, and aliphatic contents (Table 1) (Silva et al., 2015). The makeup is complex, with proteins accounting for 80% of the total. Proteins generally have large molecular weights, while peptides have lower molecular weights. Biogenic amines, such as dopamine, histamine, and serotonin, are crucial low-molecular-weight compounds found in BV. BV peptides, like adolapine, melittin, apamin, and peptide, have been extensively studied for their bioactivities and potential therapeutic applications (Isidorov et al., 2023).

**TABLE 1:** The biological significance of bee venom and its constituent ingredients.

Composition	Nature	Effect	Reference
Melittin	Peptide	The peptide has biological action. Melittin inhibits blood coagulation, inhibits pathogens, and protects against radiation. Melittin acts as an anti-inflammatory in tiny doses. It possesses both hemolytic and cytotoxic properties.	Jafari et al. (2024)
Phospholipase A2	Enzyme	Phospholipase is the most allergenic and damaging ingredient in bee venom.	Lee and Bae (2016b)
Hyaluronidase	Enzyme	This enzyme permits venom to enter tissues by widening blood vessels and boosting tissue permeability, increasing blood circulation.	Khan et al. (2018)
Acid phosphatase	Enzyme	Allergen.	Kim and Jin (2016)
Apamin	Peptide	Bioactive polypeptide: a neurotoxin.	Gu et al. (2020)
Mast cell degranulating peptide	Peptide	Causes the release of biogenic amines.	Starkl et al. (2022)
Protease inhibitor	Enzyme	It possesses anti-inflammatory and bleeding-related characteristics and suppresses proteases.	Lee et al. (2023)
Adolapin	Peptide	Anti-inflammatory, antirheumatic, and painkiller.	Bindlish and Sawal (2024)
Histamine	Biologic amine	It expands blood vessels and promotes the permeability of capillaries. It’s an allergy.	de Graaf et al. (2021)
Dopamine, noradrenaline	Biologic amine	Neurotransmitters have an impact on sensory perception and function.	Ahmed-Farid et al. (2021)
Alarm pheromone	Peptide	It sets the entire community on heightened alert.	López-Incera et al. (2021)
Secapin	Peptide	-Antibacterial -Anti-elastolytic -Anti-fibrinolytic	Lee et al. (2023)
Procamine A,B	Peptide	Suppresses the action of proteolytic enzymes, hence lowering inflammatory responses.	Răsinar et al. (2023)

The appropriate collection method is crucial to ensure the highest quality BV. It is essential to collect the venom without impurities from pollen, honey, or other colony byproducts to maintain its purity and efficacy (Essam Elenany, 2024). While there are no formal quality criteria for BV due to its classification outside the regulatory frameworks for medications and food products, researchers have suggested using quantitative analysis of stable components and comprehensive characterization as indirect means of assessing the purity and quality of BV. Additionally, efforts have been made to standardize and manage the quality control of Hymenoptera venom, including BV, to ensure its therapeutic efficacy (Oliveira Orsi et al., 2024).

### 3.1 Melittin

**FIGURE 1:**
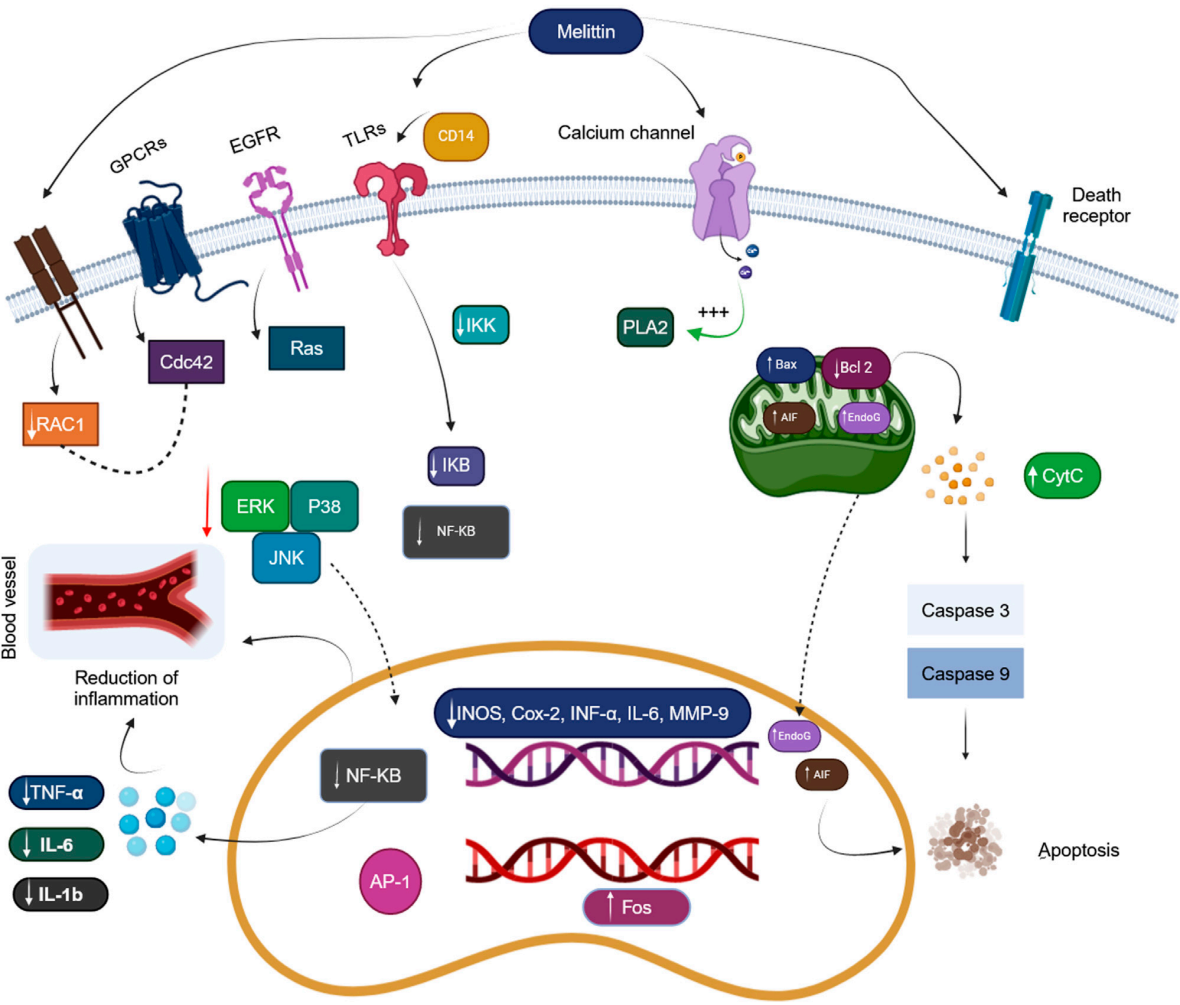
Mechanistic actions of bee venom melittin. (Generated by BioRender software).

MEL is BV’s primary potent physiological ingredient, representing 40%–50% of the dry mass. It can dissolve in linear, cationic, and hemorrhaging and weighs 2,840 Da. It is made up of 26 amino acids. The molecular structure C_131_H_229_N_39_O_31_, with the N-terminal part predominantly hydrophobic due to +4 charges and little lytic activity. The area located at the C-terminus is hydrophilic due to the +2 protons, responsible for the lytic activity, for a total of +6 charges at physiological Ph (Bava et al., 2023). Due to its amphipathic structure, Melittin is water soluble in monomeric and tetrameric forms. The previous study found that MEL possesses antimicrobial and anti-cancer benefits. It can mechanically and chemically damage many cellular membranes (Shi et al., 2016). MEL binds to the negatively charged membrane surface (Figure 1). As a result, it compromises the functioning of phospholipid bilayers by creating pores, atomic ion, and molecule leaks, and enhanced permeability, ultimately leading to cell lysis (Jamasbi et al., 2016). Melittin connects to membranes as monomers but works across the whole membrane. According to the dosage, this biopeptide might induce transitory or stable holes. Only ions can travel through the membrane when a transient pore is formed. When regular holes emerge, the membrane becomes accessible to big compounds like glucose. Melittin has recently been discovered to activate and sensitize nociceptor cells, resulting in neuronal plastic changes along pain signaling pathways. Similarly, melittin can function as a PLA2 activator. It is also a critical physiologically active component of BV, generating numerous biological effects when administered to the patient’s acupoint (Wehbe et al., 2019).

### 3.2 Apamin

Apamin is considered the second most abundant active peptide identified in BV. It represents 1% of all dried BV. This peptide possesses anti-inflammatory and antinociceptive qualities that enhance defensive line capacity. Following crossing across the blood-brain barrier, it enters the brain and affects the neurological system. Apamin suppresses vascular smooth muscle cell migration and growth in the vascular wall by targeting the Akt and Erk signaling pathways. It suppresses Erk1/2 and Akt phosphorylation generated by PDGF-BB. Apamin has been shown to block the G0/G1 cell cycle via the PDGF-BB signaling pathway, making it a possible therapy for atherosclerosis (Kim JungYeon et al., 2015).

Its structure consists of a disulfide bridge between cysteine residues 3 and 15, which is crucial for its biological activity. Apamin is a selective inhibitor of small-conductance calcium-activated potassium (SK) channels, exhibiting nanomolar or even subnanomolar affinity. This specificity allows apamin to modulate SK channel activity without affecting other ion channels or receptors. Apamin has been investigated for its therapeutic potential in various diseases, including ataxia, epilepsy, and inflammatory conditions (Kuzmenkov et al., 2022). The peptide’s ability to modulate SK channel activity has been linked to its therapeutic effects, as SK channels play a crucial role in neuronal excitability and synaptic plasticity. Additionally, apamin has been shown to have anti-inflammatory and anti-fibrotic effects, making it a promising candidate for the treatment of chronic diseases. Apamin has been explored for its potential in neuroprotection and neuroregeneration (Kim H. et al., 2021). Studies have demonstrated that apamin can cross the blood-brain barrier and bind to SK channels in the central nervous system, potentially contributing to learning and memory control. Furthermore, apamin has been shown to have neuroprotective effects in models of neurodegenerative diseases, such as Alzheimer’s and Parkinson’s. Apamin has also been investigated for its potential in cancer treatment (Villa et al., 2020). Studies have suggested that apamin can inhibit the growth of cancer cells by modulating SK channel activity and inducing apoptosis. Additionally, apamin has been shown to have anti-tumor effects in animal models, making it a promising candidate for the development of novel cancer therapies (Gu et al., 2020; Saravanan et al., 2023; Bindlish and Sawal, 2024).

### 3.3 Mast cell degranulating (MCD) peptide

**FIGURE 2:**
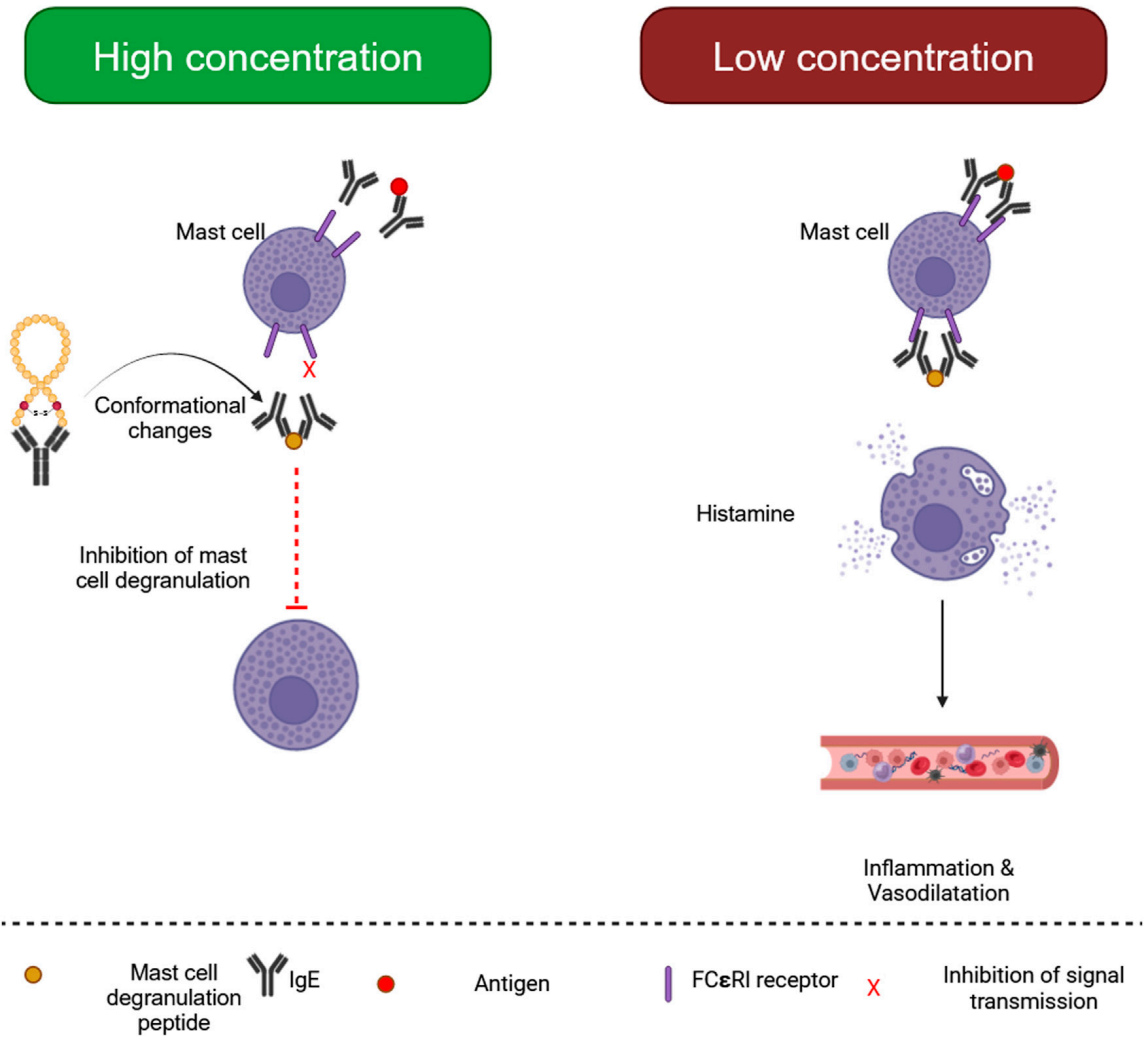
Biological processes of mast cell degranulating (MCD) peptide. (Generated by BioRender software).

Mast cell degranulating (MCD) Peptide, or peptide 401, is a BV polypeptide of 22 amino acids equivalent to apamin because it has two disulfide bridges (Răsinar et al., 2023). It makes up around 2%–3% of BV’s dry weight. The term MCD alludes to the biological function of mast cells in producing histamine. It is an epileptogenic neurotoxin, a significant antagonist of K+ channels, and may drastically drop rat blood pressure (Gajski et al., 2024). Some MCD biological activity takes different routes and might be an excellent example of the structure-function link (Figure 2). Based on research, MCD is a potent anti-inflammatory drug that might be utilized to investigate the secretion mechanisms of cells associated with inflammation, potentially leading to the creation of medicinal substances (Ullah et al., 2023).

### 3.4 Adolapin

Adolapin is a polypeptide with 103 amino acid sequences. It can be identified at 1% dry BV levels. It accounts for 2%–5% of the polypeptides present in BV (Gajski et al., 2024). Adolapin inhibits prostaglandin production and Cyclooxygenase function., providing anti-inflammation, anti-allergic, and fever resolution properties. It suppresses thrombocyte lipoxygenase activity and could possess painkilling characteristics (Sawicka et al., 2024).

### 3.5 Secapin

Secapin is a 25 amino acid polypeptide with a high proline content and one disulfide bond. It is a non-toxic polypeptide that makes up just 0.5% of dried BV. The Asiatic honey BV contains secapin (AcSecapin-1), which possesses antifibrinolytic, anti-elastolytic, and antibacterial effects (Dinata et al., 2023).

### 3.6 Procamine and tertiapin

Procamine, a polypeptide isolated from BV, suppresses protease activities, lowering inflammatory responses (Dinata et al., 2023). Tertiapin is a 21 amino acid peptide produced from BV. It has two disulfide bridges and an amidated C-terminal residue. It is a neurotoxic peptide similar to apamin. Tertiapin is a small part of the BV, representing 0.1% of its net content. Tertiapin inhibits the action of potassium gates throughout our bodies. It regulates potassium channels (Światły-Błaszkiewicz et al., 2020).

### 3.7 Phospholipase A2

**FIGURE 3:**
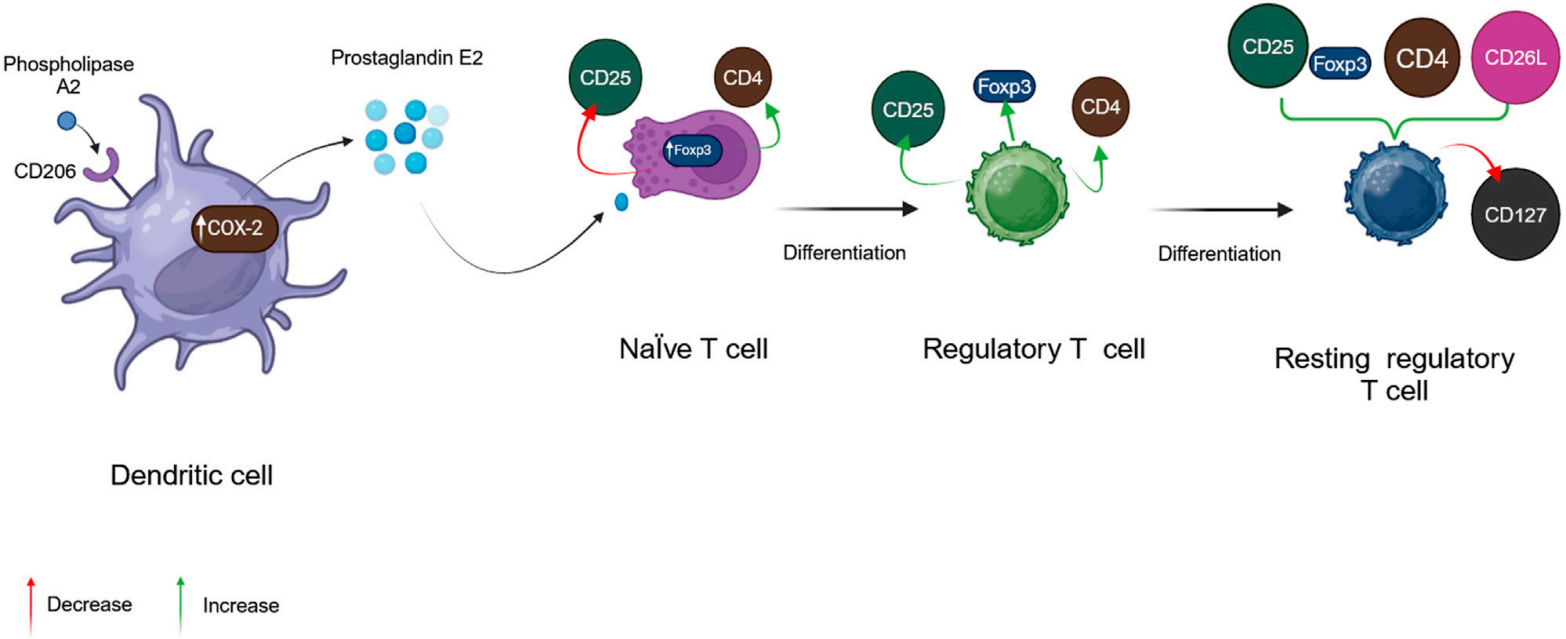
Physiological activity of phospholipase A2. (Generated by BioRender software).

Phospholipase A2 (PLA2), BV’s considerably more dangerous enzyme, is a polypeptide chain of 128 amino acids with four disulfide bridges. Its fundamental pH accounts for 12%–15% of BV’s dry mass (Gajski et al., 2024). Melittin, interestingly, can boost its action. This has been proven to occur throughout the red blood cell lysis process, showing synergistic activity between bvPLA2 and melittin (Frangieh et al., 2019). In addition, fresh, experimental results reveal that bvPLA2 has protective immunological reactions against several illnesses (Kim KH. et al., 2019). BvPLA2 causes microglial inactivation and decreases CD4+ T cell recruitment (Figure 3) (Jafarzadeh et al., 2023).

### 3.8 Hyaluronidase

Hyaluronidase amounts to about 1.5%–2% of BV dry weight and has been shown to break down hyaluronic acid. BV hyaluronidase enables the biologically functioning elements in BV to reach the targeted tissues with greater effectiveness by modifying its architectural durability and improving blood supply in the region. These two activities promote the spread of venom (Bava et al., 2023).

Aside from peptides, BV includes carbohydrates, free amino acids, and minerals. BV contains carbohydrates such as glucose and fructose. The primary components of amino acids are -aminobutyric acid and B-aminoisobutyric acid. BV also includes minerals such as magnesium, calcium, and phosphorus. It also includes volatile chemicals termed pheromones, symbolized by complicated ethers, which warn bees of hazards and trigger stinging responses (Salem and Marzouk, 2024). The RHGXRSP domain distinguishes these acid phosphatases from others. It releases histamine and creates particular IgE, which can be utilized in immunotherapy (Hossen et al., 2016). Dipeptidyl peptidase IV (DPIV) or Api m5 has a molecular mass of 102 kDa (Rzetecka et al., 2024). It is connected to transforming pro-toxins into activated metabolites (Blank et al., 2010; dos Santos-Pinto et al., 2018). Vitellogenin or Api m12 is BV’s compound with the most significant molecular weight (200 kDa) (dos Santos-Pinto et al., 2018). Furthermore, it has antibacterial and antioxidant properties (Park HeeGeun et al., 2018).

## 4 Delivery approaches of bee venom

Oral delivery is complicated because the protein composition of BV can be degraded by gastrointestinal enzymes (Carpena et al., 2020). However, a study conducted by Meligi and his group, who studied the protective potential of oral administration of BV against liver damage caused by lipopolysaccharide and carbon tetrachloride in rats contradicts other reports (Meligi et al., 2020). BV therapy is usually applied in different ways including bee stings at specified places, injections of pure and sterile Apis homeopathic preparations, BV ointments, lotions, capsules, drops, and phonophoresis. Honeybee sting therapy is considered a classic therapeutic approach. It has several disadvantages, including discomfort. Since melittin’s short half-life and the annoyance it gives patients, continuous administration of multiple rounds of stings or injections in the blood requires sting-induced inflammation and trouble maintaining average concentrations (Izhar et al., 2023). Due to BV’s short duration of action and the challenge of estimating its dosages, scientists and professionals have promoted and created alternate options, such as mixing polymers or nanoparticles (NPs) (Ojha et al., 2023; Sharma et al., 2024a).

Traditional methods of administering BV therapy have typically involved topical applications, such as intravaginal suppositories or gels containing BV extracts. These methods have been used to target specific areas affected by conditions like bacterial vaginosis (BV). However, advancements in drug delivery have introduced more sophisticated approaches to BV therapy. Controlled drug release systems now allow for precise delivery of the active ingredients, ensuring the right amount reaches the intended site of action. The shift towards advanced drug delivery methods has the potential to enhance the efficacy of BV therapy by improving targeted delivery, increasing bioavailability, and reducing side effects associated with traditional administration routes. For instance, nanotechnology-based delivery systems offer high sensitivity and specificity, allowing for rapid diagnosis and treatment of BV. These modern approaches not only improve the precision of drug delivery but also optimize therapeutic outcomes by ensuring that the active components of BV reach the target tissues more effectively.

## 5 Therapeutic mechanisms

**FIGURE 4:**
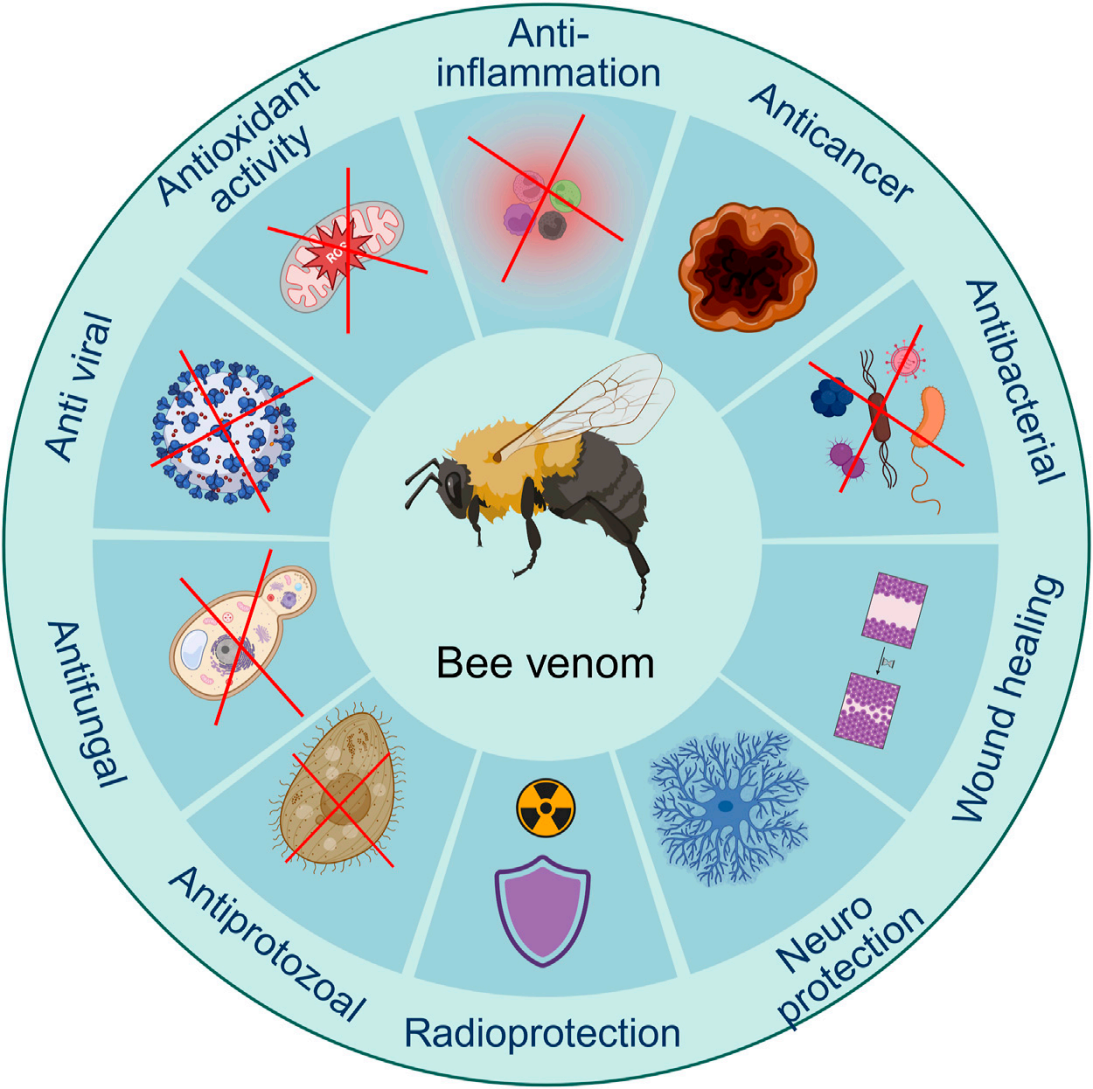
Biological activities of bee venom. (Generated by BioRender software).

According to its biological ingredients, BV has a wide variety of medicinal effects. Several studies have discovered the pharmaceutical advancement in one or several features of BV, primarily focusing on pain-relieving (Lee et al., 2005), neuroprotective (Ye et al., 2016b), anti-inflammation (Jeong et al., 2017), enhancing wound recovery (Hozzein et al., 2018), anti-microbial impacts (El-Seedi et al., 2020), and cancer-fighting (Małek et al., 2023). These show that BV has a wide variety of therapeutic uses, which might be due to its multi-target and multi-pathway features (Figure 4). The complex composition of BV, with its mixture of both inflammatory and anti-inflammatory compounds, contributes to its dual nature. While the venom can cause immediate pain and swelling upon a sting, it also has the potential to exert beneficial effects, such as reducing inflammation and providing pain relief, when used in controlled therapeutic settings (Sadek, 2023). The precise mechanisms of action for the various components of BV and their interactions are still being actively investigated. Ongoing research aims to investigate the specific pathways and targets through which BV and its constituents exert their pharmacological effects, both beneficial and detrimental, to optimize its therapeutic potential while minimizing adverse reactions (Pandey et al., 2023a).

### 5.1 Antioxidant activity

BV includes compounds with high antioxidant activity (AOA) (Martinello and Mutinelli, 2021). The phenomenon is caused by phospholipases A2, apamin, and melittin. The antioxidant action is based in many ways, such as free radical elimination, proton transfer, metallic ions binding, solitary oxygen suppressing, and serving as a trigger for removing superoxide and hydroxyl radicals (Yaacoub et al., 2023). The antioxidant benefits may be due to the substances’ capacity to impede lipid peroxidation and increase superoxide dismutase functionality. Superoxide dismutase is an enzyme that decreases tissue destruction by eliminating the superoxide radical in most cells when subjected to oxygen (Pinmanee et al., 2023).

BV includes extra antioxidants. Vitellogenin is a protective agent in mammalian cells, shielding them from oxidative stress and defending against free radicals. Few studies have employed traditional methods to determine the AOA of BV (Sobral et al., 2016; Somwongin et al., 2018). All samples included antioxidants, unconnected to the elements found and quantified in them. Evidence suggests that melittin alone has lower AOA than BV extracts, perhaps due to other metabolites (Pavel et al., 2014). Other tiny molecules may have a role in BV biological properties, with combinatorial or antagonistic effects at different concentrations resulting in varying consequences.

Previous studies found that honey BV significantly inhibited nonenzymatic lipid peroxidation. It competes with dimethyl sulfoxide for hydroxyl radicals, indicating its high detoxification properties. The findings suggest that honey BV’s antioxidant activity contributes to its anti-inflammatory characteristics, particularly its capacity to reduce interleukin-1 synthesis *in vitro* (Değer et al., 2023; Abd El-Aziz et al., 2024; Virk et al., 2024). Additional investigations assessed antioxidant capacity in combination with other criteria. For example, rabbits received 0.1, 0.2, and 0.3 mg Under the skin twice weekly for 20 weeks (El-Hanoun et al., 2020). The study found that treating rabbits boosted glutathione S-transferase (GST) and glutathione (GSH) levels. The readings of malondialdehyde (MDA) and thiobarbituric acid–reactive substances (TBARS) were also lower. These studies confirmed BV’s antioxidant capabilities. BV improved reproductive performance and semen antioxidant activity. Elkomy et al. (2021) found delivering BV via subcutaneous injection to rabbits improved total antioxidant capacity (TAC). Kim et al. introduced doses ranging from 10 to 500 μg per kilogram to the standard diet (Kim D-H. et al., 2019). The scientists found that ingesting BV improved the antioxidant properties and altered fatty acid metabolism in broilers.

### 5.2 Anti-inflammatory activity

Melittin may induce itching, inflammation, and localized discomfort in large dosages, although lower doses of melittin offer widespread anti-inflammatory benefits (Sawicka et al., 2024). Melittin inhibits the Toll-like receptors (TLRs) 2,4, CD14, nuclear factor-κB essential modulator (NEMO), and Platelet-derived growth factor receptor (PDGFR) signaling pathways. This decreases inflammatory reactions in numerous tissues (Lee and Bae, 2016a). BV has anti-nociceptive properties for inflammatory pain. BV has been utilized for decades to treat numerous discomfort reactions, including visceral, inflammatory, and heat (Ullah et al., 2023). Hypodermic apipuncture with BV decreases thermally and mechanically related hyperplasia Arthritis discomfort is caused by collagen (Elieh Ali Komi et al., 2018). Furthermore, a single ingredient of BV, adolapin, has analgesic effectiveness. Pre-treatment with BV has anti-nociceptive properties against the spinal cord. Fos activity correlates with formalin-generated discomfort in rats (Galante et al., 2023). Apamin is a pain reliever that reduces inflammation and muscle discomfort (Wehbe et al., 2019). Oxaliplatin can cause mechanical allodynia and coldness; however, phospholipase A2 can mitigate these effects (Hossen et al., 2017a). Subcutaneous injection of BV has been demonstrated to offer anti-nociceptive effects in mouse models for both visceral and somatic pain. Additionally, it prevents nociceptive responses in mice caused by acetic acid (Qi et al., 2023). BV is more effective than adjuvants for treating arthritic pain because of its anti-nociceptive properties (Ullah et al., 2023). BV acupuncture effectively alleviated neuritis pain caused by paclitaxel in mouse models (Shen et al., 2020).

### 5.3 Anticancer activity

Cancer is one of the most severe diseases harming humans and is one of the leading causes of death around the globe. According to current data, around 10 million freshly diagnosed patients with the illness each year, and over 6 million people die from it, accounting for around 12% of all fatalities globally. In 2020, 15 million new cancer cases will be diagnosed (Morgan et al., 2023). Cancer growth and advancement are complex activities. Smoking, pathogenic organisms, environmental contaminants, a lousy diet, and internal variables that include transmitted down anomalies in genes, hormonal changes, and immunological conditions might all contribute to the start of this illness (Golubnitschaja et al., 2016). BV has been frequently used to treat several malignancies (Varol et al., 2022). BV metabolites have received a lot of interest for their possible utility in oncotherapy. Melittin, a powerful antitumor polypeptide, maybe a more practical alternative than the entire BV (Rady et al., 2017).

Melittin has high membrane-perturbing action, which accounts for its anti-inflammatory, anti-microbial, and cancer-fighting abilities (Roversi et al., 2023). When numerous melittin particles enter the cell membrane, they degrade the phospholipids, resulting in cellular fragmentation. It breaks down both phospholipid and synthesized bilayers. It has been found to cause apoptosis and have anticancer properties. Melittin targets several tumor cell types, including kidney, hepatic, pulmonary, prostate, and urinary malignancies, in addition to malignancies of the breast and leukemia (Zarrinnahad et al., 2018).

### 5.4 Anti-microbial activity

Microbial infections are a challenge, especially with the growth of medication resistance; thus, scientists are seeking Recent discoveries of bioactive potential. Products from nature are considered sources of sustainability with fewer problems that can provide various active substances (Thawabteh et al., 2019).

#### 5.4.1 Anti-bacterial activity

Blending BV with regular medications is effective, helpful, and safe. Against specific microorganisms, but its uses are being carefully considered in both preclinical and clinical studies. BV and melittin have wide-ranging antibacterial properties against Gram-positive and Gram-negative bacteria, with MIC values ranging from 10 to 100 μg/mL and 30 to >500 μg/mL, respectively. BV, melittin, and oxacillin effectively killed Methicillin-resistant *Staphylococcus aureus* (MRSA) ATCC 33591. BV and melittin treatment led to modifications in the bacterial cell membrane due to membrane integrity loss and cellular shape changes involving cellular deformation. And cytoplasm loss (Pereira et al., 2020). Melittin’s antibacterial capacity was tested against numerous bacteria (Roversi et al., 2023). Gram-positive bacteria are more sensitive to melittin compared to Gram-negative bacteria due to variations in their cell membrane structures (Gong et al., 2023). Melittin may pass through the peptidoglycan layer of Gram-positive cell membranes more effectively than Gram-negative cell membranes, which are protected by a lipopolysaccharide layer. The proline residue at position 14 has been proven to play a crucial role in melittin’s antibacterial action (Sharma et al., 2024b). Melittin can dissolve biofilms generated by *S. aureus* and *Escherichia coli* (Rouhi et al., 2024).

#### 5.4.2 Anti-viral activity

Viral infections have appeared in the previous 10 years. These have jeopardized the lives of millions of people throughout the world, particularly immunocompromised individuals. BV and its constituents have significant antiviral activity against viral infections (Table 2) (Mohanty and Murhekar, 2023). An enhanced understanding of Melittin’s processes will help us enhance antiviral approaches. Several antimicrobial peptides (AMPs) primarily disrupt membranes to achieve their effects. Melittin’s direct interaction with viral capsid proteins or envelopes in this scenario hinders the binding or absorption of cells capable of producing viruses (Memariani et al., 2020). Furthermore, additional possible antiviral modes of action include inhibiting viral multiplication, reducing viral mRNA expression levels, producing changes in viral genome conformation (Memariani et al., 2020), inhibiting the virus’s cytotoxic properties, and inhibiting virally mediated cell fusion (Mohanty and Murhekar, 2023), and inhibit viral bundling (Peng et al., 2023). The possible use of melittin-loaded nanoparticles to eliminate the human immunodeficiency virus while leaving uninfected cells alone suggests a preventative method in which these nanoparticles are employed to make a vaginal lotion that stops HIV transmission. The theoretical underpinning is that Melittin molecules may be discovered on the viral envelope. Pore-like attack complexes develop when nanoparticles fuse (Peng et al., 2023). Another research discovered that bvPLA2 can similarly inhibit viral replication. The same team found the bvPLA2 peptide sequence, suppressing HIV replication (Wehbe et al., 2019).

**TABLE 2:** Application of bee venom in controlling the viral diseases.

Bee venom/isolated compounds	Model	Technique	Outcome	Reference
Phospholipase A2 (sPLA2)	Hepatitis C virus (HCV)	Plaque assay	PLA2 and its related compounds are promising prospects for the establishment of wide-spectrum antiviral medications targeting viral outer lipid bilayers produced from the endoplasmic reticulum membrane.	Chen et al. (2017)
Melittin	Human immunodeficiency virus (HIV)	Transient transfection Assays	Blocks viral replication. Blocks transcription of genes. Reduces intracellular protein and RNA production. Reduces the long terminal repeat (LTR) activity with an ID50 value. 0.9–1.4 µM after 24 h.	Wachinger et al. (1998)
Bee venom	Herpes simplex virus (HSV)	Plaque assay	Limits viral propagation EC50 1.52 ± 0.11 μg/mL	Uddin et al. (2016)
Bee venom	Papillomaviruses (HPV16 E6)	Reverse transcription assay	Blocks mRNA transcription. Blocks cell proliferation. Reduces protein levels. At 10 µg/mL, it decreases 0.35 ± 0.06-fold after 24 h.	Kim et al. (2015c)

#### 5.4.3 Anti-fungal activity

Fungal infections harm over a billion people globally and cause about 1.5 million deaths each year (Silva et al., 2019). Antifungal drugs have specific restrictions concerning biocompatibility, spectrum of action, and pharmacological characteristics. These concerns are worsened when these medicines can be utilized extensively and inappropriately, hastening the evolution of drug-resistant mutations over time (Smith et al., 2019).

The shortage of novel antifungal drugs under development needs the creation of potent antifungal agents, particularly those derived from natural ingredients. BV is effective against various fungi at micromolar doses (Table 3). Furthermore, emerging evidence suggests that melittin inhibits fungal growth via several processes, such as membrane permeability and apoptotic activation via the ROS-induced mitochondrial/caspase-dependent route, suppression of (1,3)-D-glucan synthase, and alterations in fungus genetic transcription. Animal models should be employed to evaluate melittin’s safety and effectiveness in the future. Melittin will likely offer new paths for numerous biological uses, from medicine to agriculture (Memariani et al., 2019).

**TABLE 3:** Applications of bee venom in controlling the fungal infections.

Bee venom/isolated compounds	Microbe	Technique	Outcome	Reference
Apamin	*Alternaria alternate*	Not reported	Melittin and apamin could prevent the fungal-related synthesis of inflammatory messengers and extracellular matrix (ECM) by nasal fibroblasts.	Shin et al. (2017)
Melittin	Unicellular fungal (UCF) pathogens (*Cryptococcus neoformans*, *Kodamaea ohmeri*, and *Candida albicans*)	automated Vitek2 system	BV/chitosan nanoparticles (CHNPs) inhibited biofilm development and the yeast-to-hyphal transformation of the investigated (UCF) pathogens. These findings demonstrated that BV-chitosan nanoparticles are a potential natural chemical for treating fungal diseases.	El-Didamony et al. (2022b)
Bee venom/melittin	*C. krusei*	Broth microdilution	Bee venom and melittin had intense antifungal action (MIC measures ranging from 30 to 100 μg/mL).	Issam et al. (2015)
Bee venom	*C. albicans*	Disc diffusion	Suppresses fungal dimorphism	Ali (2014)
Bee venom	*Trichophyton rubrum*	Disk diffusion method	The bee venom in its entirety provided a high amount of suppression, and the venom in isolated forms was ineffective. Furthermore, BV-based compounds may serve as promising antifungal treatments.	Park et al. (2018)

#### 5.4.4 Anti-protozoal activity

Investigations have shown that BV category III sPLA2 possesses anti-trypanosomiasis—activity BV group III sPLA2 transcription in a genetically engineered mosquito’s midgut inhibits Plasmodium ookinetes Cecropin, a melittin hybrid, effectively inhibits Leishmania Donavan promastigote by damaging its plasma membrane (Ullah et al., 2023). Melittin affects membrane integrity in prokaryotic and eukaryotic species, leading to membrane lysis and increased permeability. Melittin exhibits antibacterial, anti-fungal, and antileishmanial properties due to this interaction (Berhe et al., 2024). Previous studies suggest that in controlled circumstances, PLA2 has antiprotozoal activity against *Trypanosoma brucei*. Lasioglossins, a peptide found in BV, have potent antibacterial properties due to their membrane contact and DNA binding (Ullah et al., 2023). The peptide melittin has been shown to have antiprotozoal efficacy versus *Toxoplasma gondii*, *Trypanosoma cruzi*, *Plasmodium*, and *Leishmania*. Melittin has been used in vaccine production to boost protection against leishmaniasis. Melittin can kill *T. cruzi* (Memariani and Memariani, 2021). Although BV has antiprotozoal properties, its specific impact, route of action, and potential for therapeutic use remain unclear.

### 5.5 Wound healing

The reconstruction of wounds is an intricate mechanism that consists of four concurrent stages: hemostasis, clot formation, inflammation, and division (Duda et al., 2023). The research provided demonstrates that BV treatments have an impact on all stages of the healing process. BV can be used topically or subcutaneously on wounds (Table 4). An animal model study suggests BV might be a viable damage-healing therapy for diabetes patients. BV promotes the initial stage of wound healing (hemostasis) in diabetic mice by increasing the production of Transforming growth factor beta (TGF-β) and Vascular endothelial growth factor (VEGF).

**TABLE 4:** Application of bee venom in wound healing.

Active component	Action	References
Tertiapin	Anti-inflammatory properties action via inhibiting potassium gates.	Dadar et al. (2019)
Secapin	Anti-fibrinolytic, anti-elastolytic, and antimicrobial properties	Lee et al. (2016a)
Peptide 401 The mast cell degranulation peptide	Benefits include relief from pain, as well as anti-inflammatory and nociceptive effects.	Bellik (2015)
Melittin	Antioxidant, anti-inflammatory, and pro-angiogenic.	Shaik et al. (2023)
Hyaluronidase	Breaks down hyaluronic acid, allowing the venom to penetrate tissue. It causes dilatation and greater blood artery permeation, increasing blood circulation.	Son et al. (2007)
Apamin	Inhibits cyclooxygenase-2 and phospholipase A2.	Kim et al. (2020b)
Adolapin	Antioxidative, anti-apoptotic, and anti-inflammatory.	Han et al. (2011)
Acid phosphatase	Human-sensitized basophils produce a potent histamine releaser. It can be used in anti-BV immunotherapy.	Grunwald et al. (2006)

Activating Transcription Factor 3 (ATF3, and inducible nitric oxide synthase (iNOS) has been shown to impact on extracellular matrix biosynthesis through increased collagen synthesis (Wang et al., 2021b). In the Reepithelization stage, BV triggers human epidermal keratinocyte division and migration while decreasing interleukin-8 (IL-8) and Tumor necrosis factor-α (TNF-α). BV affects angiogenesis and neovascularization in diabetic mice by inhibiting caspase-3, -8, and -9 activity while enhancing TGF-β and VEGF levels. TGF-1, fibronectin, and VEGF levels are lowered during remodeling, whereas raising collagen-I mRNA levels reduces the expression of ATF-3 and Inos (Kurek-Górecka et al., 2020).

### 5.6 Neuroprotection

Neuroinflammation induced by prolonged stimulation of glial cells and microglia is linked to neurodegenerative disorders. Various BV components, such as PLA2 and apamin, have been investigated as anti-neuroinflammation medicines to boost the efficiency of multiple treatments against neurodegenerative illnesses (Mohammadi-Rad et al., 2019). It has been proposed that melittin might help address neurodegenerative disorders linked to stimulation of microglial cells since it substantially inhibits BV2 microglia’s pro-inflammatory activities (Zhang et al., 2024). The progressive neurodegenerative illness amyotrophic lateral sclerosis (ALS) damages motor neurons in the brain and spinal cord, resulting in muscle weakening and atrophy (Zarei et al., 2015). Yang et al. found that melittin therapy enhances the anti-inflammatory ability of the proteasome in the central nervous system (CNS) of Amyotrophic Lateral Sclerosis (ALS) model mice (Yang et al., 2011). Melittin-treated mice demonstrate less neuronal loss in the spinal cord and better motor control, which enhances motor performance. In addition, melittin therapy lowers p38 phosphorylation and the microglial cells count in the brainstem and spinal cord. Furthermore, melittin inhibits-synuclein post-transcriptional modification, a critical ALS pathogenic pathway, and activates chaperones to prevent protein misfolding. The findings support melittin’s anti-neuroinflammatory properties. Although the CNS is the major issue with ALS, the liver, spleen, and lungs are all impacted. In an animal model of ALS, melittin therapy lowers inflammation while increasing, suggesting cell viability in the spleen and lung (Lee and Bae, 2016a). AD, the most common neurodegenerative disease, comprises several pathogenic mechanisms that contribute to its development (Vickers, 2017). Although the etiology of Alzheimer’s disease is still unknown, research suggests that inflammatory reactions may play an important part. In its progression (Cummings et al., 2018). Current therapies for cognitive loss caused by Alzheimer’s disease include acetylcholinesterase (AChE) inhibitors and muscarinic or nicotinic receptor ligands. As an alternative strategy, bvPLA2 was shown to be effective in slowing the course of Alzheimer’s disease in transgenic mice. This is attributed to. bvPLA2’s capacity to minimize accumulation while improving cognitive function in mice brains. It also reveals that bvPLA2 can enhance brain glucose metabolism and lower the hippocampus’s neuroinflammatory reactions, potentially inhibiting AD progression (Ye et al., 2016b). Recent research has also shown that bvPLA2 therapy in a 3xTg-AD mouse model might affect regulatory T-cell populations. To prevent AD’s unfavorable inflammatory response, scientists developed a novel therapeutic method that combines bvPLA2 medication with AB vaccine therapy (Baek et al., 2018). In addition to creating protective benefits against inflammatory disorders, bPLA2 possesses anti-inflammatory actions, as previously stated (Palm et al., 2013). A recent study investigated PD in mice using a combination of 78% bPLA2 and 15% melittin (Carpena et al., 2020). bPLA2 reduced inflammation by activating Treg cells. Additionally, bPLA2 activation reduced the loss of dopaminergic cells (Kim KH. et al., 2019).

### 5.7 Radioprotective activity

Research indicates that BV can protect against the detrimental effects of ionizing radiation. In many evaluation systems, BV protects against gamma and X-ray radiation (Djahonkulovna, 2023). Additionally, BV contains antioxidant properties that neutralize free radicals while shielding the body from harmful radiation ()Shaaban et al., 2019. BV protects bone marrow cells from chromosomal mutations (aberrations) in the Wistar mouse model (Yu et al., 2023). It promotes hematopoiesis, induces the production of histamine from MCD, and lowers blood oxygen pressure through phospholipase A2 (Gajski et al., 2024). PLA2, a BV component, contains a forkhead box P3 (Foxp3), + CD4, + CD25, + Treg cell that protects against radiotherapy-induced extensive pulmonary inflammation (Hossen et al., 2017b). BV therapy significantly lowers levels of IL-6 and TNF-a following gamma radiation therapy (Hasan et al., 2023). BV decreases serum Aspartate transaminase (AST), Lactate Dehydrogenase (LDH), Creatine Kinase MB (CK-MB), Cardiac Troponin I (cTnI), and Alanine aminotransferase (ALT) concentrations in Wistar mice via decreasing hepatic NF-kB transcription. However, the amounts rose following gamma radiation (Salimo et al., 2023). According to investigations, BV exhibits radioprotective properties against oxidative and basal DNA damage (Montoro et al., 2023). Administering melittin a day before being subjected to the radiation (8.5 Gy) dramatically improves survival rates in mice (Duché and Sanderson, 2024). BV shields human peripheral blood lymphocytes against damaging gamma radiation (Yu et al., 2023). In an analogous study, BV was shown to protect blood cells against radiation (3–4 Gy) when administered 24 h beforehand (Khateeb and Taha, 2024). Contemporary technologies and non-ionizing radiation have become more prevalent daily, leading to detrimental consequences on the body. BV has demonstrated radioprotection, making it a potential non-toxic and efficient radioprotector agent for the future.

## 6 Medical applications

BV is a well-known pharmacologically active product of the hive synthesized by the venom glands associated with the sting apparatus of workers and queens, stored in the venom reservoir, and injected through the sting apparatus during the stinging process (Aparna, 2020). Due to its anti-oxidants, anti-coagulants, anti-inflammatory properties, and bioactive substances like melittin and phospholipase BV is mainly used to treat many inflammatory disorders such as arthritis, cancer, diseases of the nervous system, heart and blood system abnormalities, skin diseases, and others (Khalil et al., 2021). Furthermore, therapeutic application of BV includes their use in the management of bursitis, tendonitis, dissolving scar tissue, in the management of post herpetic neuralgia, Lyme disease, rheumatoid arthritis, osteoarthritis, multiple sclerosis, TMDs, and more (Table 5) (Kurek-Górecka et al., 2020).

**TABLE 5:** Physiochemical properties, doses, and benefits of bee venom, and its components in numerous medical applications.

Bee venom/Metabolites	Physiochemical properties	Disease	Model	Method of application	Dosage of use	Outcomes	References
Bee venom	Anti-inflammation and damaging effects on cells on several kinds of tumour cells.	Liver carcinoma Breast cancer Cervical cancer	Liver (Hep-G2), breast (MCF-7), and cervical (HPV-18 infected HeLa cells)] and two normal cells (splenocytes and macrophages (MQ		NA	Having an effect on IL-10, TNF-α, and IFN-γ. Increased Cas3 levels.	Salama et al. (2021)
	Inhibiting cervical-cancer tumorigenesis	Cervical cancer	HPV-positive cervical-cancer cell lines, such as Caski and HeLa cells, and not to HPV-negative cervical-cancer cells (C33A)		0, 0.625, 1.25, 2.5, 5, and 10 μg/mL of BV for 24 h	Cyclin A, cyclin B, Bcl-2, and Bcl-XL levels decreased. Effects on caspases and intercellular signaling pathways.	Kim et al. (2020a)
	Small conductance K+ (SK) channel inhibition may be effective in managing Parkinson’s disease in both short and long term.	Parkinson’s disease	Human	Injection	s.c. bee venom injections (100 μg)	Delayed disease progression.	Hartmann et al. (2016)
	Significant decrease in muscle tonus.The VAS scale decrease was statistically significant.	Temporoma ndibular disorder (RDC/TMD Ia and RDC/TMD Ib)	Human	Local dermal massage to the area of masseter muscle	0.0005% bee venom ointment	Relived masticatory muscles myalgia	Nitecka-Buchta et al. (2014)
	Increase locomotor velocity. ↑ Parkinson’s Disease Quality of Life Questionnaire. ↑ Motor manifestations	Parkinson’s disease (PD)	Human trial	Acupuncture	0.1 mL, diluted to 0.005% twice a week for 12 weeks.	significant improvements in gait speed	Doo et al. (2015)
	(RLPP) ↓ TNF-α	Recalcitrant localized plaque psoriasis (RLPP)	Human trial	Intradermal injection	Inject 0.05–0.1 mL of commercial BV (Epivac^®^) once a week for 3 months.	↓ Recalcitrant localized plaque psoriasis	Eltaher et al. (2015)
	(i) Lower blood glucose levels (ii). Reduced blood cholesterol concentrations, reduced low density lipoproteins, and boosted high density lipoproteins.	Diabetes	Rabbits	A bee sting	Includes 0.2–0.5mL of BV for 14 days.	Management of hyperglycemia and hyperlipidemia	Khulan et al. (2015)
	Remarkable improvement in blood levels of TNFα (−14%), IL1β (−52%), IL-6 (−53%), NFKB (−32.6%), and ESR (−39.8%).	Chronic Low Back Pain	Human trial	Bee stinging at GV14 acupuncture sites.		Effectively managed low back pain.	El Gendy et al. (2017)
	Destructive and apoptosis outcomes.	Breast cancer	MCF-7		2.5, 5, 7.5, 10, 12.5 (µg/mL)	CBV (in a dose-related fashion). NO generation, caspase-3 stimulation, MCF-7 survival, functioning of catalase, and glutathione level. CBV caused destruction of DNA in MCF-7 cells in a dosage-dependent way.	Kamran et al. (2020)
Melittin	Reduced amino acids in the proline/glutamine/arginine system. Reduced amounts of carnitines, polyamines, ATP, and NAD+.	Ovarian cancer	Human ovarian cancer cell lines A2780 (cisplatin-sensitive) and A2780CR (cisplatin-resistant)		4.5, and 6.8 μg/mL	Anti-cancer activity	Alonezi et al. (2016)
	Increased apoptosis. Ca2+ is released from the endoplasmic reticulum and accumulates in the mitochondria.	Leukemia	Acute lymphoblastic leukaemia (CCRF-CEM) and chronic myelogenous leukaemia (K-562)		0.001–100 µM	Disruption in mitochondrial membrane potential, annexin V binding, and caspase 3/7 activation.	Ceremuga et al. (2020)
	Anti-angiogenic, and anti-inflammatory.	Lung carcinoma	Mouse		0.1–5 mg/kg	Reduced the number of macrophages in the tumor environment and reduce VEGF and CD206 transcription in bone marrow-derived M2 macrophages.	Lee et al. (2017)
	Anti-inflammatory.	Liver inflammation	Mouse	i.p. injection, twice a week for 12 weeks	0.1 mg/kg	Reduced inflammation, fibrosis, and the production of VCAM-1, IL-6, and TNF-α in the liver.	Park et al. (2011)
	Anti-inflammatory	Neuro inflammation	*In vitro*		0.5–2 μg/mL	Reduced NO and iNOS levels Repressed NF-κB activity by inhibiting IκBα degradation and JNK and Akt phosphorylation.	Moon et al. (2007)
	Antitumor activity, and anti-angiogenic ations.	Non-small cell lung cancer	Human non-small cell lung cancer cell line, A549	subcutaneous	1 and 10 mg/kg	Lowering of HIF-1α and VEGF levels.	Zhang and Chen (2017)
	Anticancer	Malignant melanoma	Human malignant melanoma (A375) cells		3, 4 and 6 μg/mL	Increased expression of cytochrome c leads to its translocation to the cytosol, as well as Cas3 and Cas9. Decline in EGFR transcription.	Sangboonruang et al. (2020)
	Anti-cancer effects	Gastric cancer Colon cancer	AGS, COLO205 and HCT-15 cell lines		10–20 μg/mL	Membrane damage.	Soliman et al. (2019)
	Anti-inflammatory properties 100 times more potent than hydrocortisone Antimicrobial properties antinociceptive effect Antitumor activities Radioprotection	Atopic dermatitis	Mouse	TNF-α/IFN-γ stimulated human keratinocytes	(100, 200, and 500 g mixed with normal saline) was applied 5 times/week for 4 weeks	Reduced ad skin lesions. BV and melittin inhibited the generation of chemokines (e.g., CCL17 and CCL22) and cytokines associated with inflammation (e.g., IL-1β, IL-6, and IFN-γ) generated by 2,4-dinitrochlorobenzene.	An et al. (2018)
		Arthritis	raw 264.7 and synoviocytes obtained from patients with rheumatoid arthritis, *in vitro*		5–10 μg/mL	Blocked the transcription of LPS-induced COX-2, PGE2, cPLA2, NO, and iNOS Reduced JUK and NF-κB activation, IκB release, and nuclear translocation of the p50 subunit.	Park et al. (2004)
		Amyotrophic lateral sclerosis	Mouse, s.c. injection at ST36 acupoint three times a week		0.1 μg/g	Microglia and phospo-p38 levels have decreased in the spinal cord and brainstem. Improves motor abilities and prevents neuronal death in the spinal cord. Inhibited a-synuclein misfolding. Reduced transcription of Iba-1 and CD14 in the lung Obliterated expressed CD14 and COX-2 in the spleen. Elevated pERK and Bcl-2 in the spleen	Lee et al. (2014b)
	Anticancer activity	Osteosarcoma	Human MG63 osteosarcoma cells		1 µM	Stimulation of phospholipase A2 leads to cell death and Ca^2+^ influx, whereas inhibition of cell proliferation occurs via the stimulation of inositol-requiring protein-1a and Xbox-binding protein 1.	Chu et al. (2007)
Apamin	Anti- apoptosis, and anti-inflammatory actions	Atherosclerosis	THP-1 cell treated with oxLDL - LPS injection with high fat diet		0.5, 1 and 2 μg/mL	Reduced NF-κB signaling pathway.	Kim et al. (2012)
	SK channel blockage. Increased the action potential duration	Heart failure	Pacing induced heart failure	Rabbit	(100 nmol/L) infusion	Reduce reduced ventricular fibrillation (VF) vulnerability.	Yin et al. (2017)
	Blocked MAPK, Smad, and TGF-_1 signalling pathways.	Liver fibrosis	HSC-T6 cells, an immortalized rat hepatic stellate cell line		0.5, 1 and 2 μg/mL	Suppressed hepatic fibrosis	Kim et al. (2017)
	Improved memory acquisition	Alzheimer’s disease	Transgenic mouse			Enhanced efficiency of nicotinic signaling	Proulx et al. (2020)
	Increased membrane potential in postsynaptic cell	Neurofibromatosis	Heterozygous Nf1+/mouse model			SK channel blockage.	Kallarackal et al. (2013)
Mast-Cell Degranulating (MCD) Peptide (Peptide 401)	Anti-inflammatoryactivity	Edema	Carrageenin-induced swelling of the Rathin paw		10 mg/kg	Suppressed edema	Banks et al. (1990)
Adolapin	Analgesic, and anti-inflammatory actions	Brain, and spleen inflammations	Rat spleen and brain		40 μg/kg	Inhibited cyclooxygenase. Elevation of c-GMP level. Decrease of c-AMP.	Koburova et al. (1985)
Phospholipase A_2_	Neural protective effect	Multiple sclerosis (Experimental autoimmune encephalomyelitis)	C57BL/6 mouse	Injection	(0.2 mg/kg, i.p., daily for a period of 10 days)	bvPLA2 reduces limb paralysis and CD4+ cell invasion, but loses its benefits when Tregs are reduced.	Lee et al. (2019b)
	Anti-inflammatory and anti-arthritic activities	Rheumatoid arthritis	Male DBA/1 mouse	Intraperitoneal injections	(0.1, 0.5, 1.0 mg/kg, i.p., for 5 weeks)	Prevent bodily weight reduction. Reduce squeaking score. Paw thickness and arthritis index.	Choi et al. (2021)
	Alleviated atopic skin inflammation	Atopic dermatitis (AD)	Male C57BL/6 mouse	Alleviates atopic skin inflammation	(80 ng/ear, topical application, four times a week for 3 weeks)	Reduce atopic dermatitis-related skin edema, optimize ear thickness, reduce Th1 and Th2 cytokine expression, promote Treg, reduce epidermal and dermal thickness and macrophage invasion, and inhibit mast cell penetration.	Shin et al. (2018)
Hyaluronidase	Sperm like structure	Male Infertility	*In vitro*			Substantial homology with PH-20, a membrane protein of guinea pig sperm that regulates sperm-egg attachment.	Gmachl and Kreil (1993)
Acid phosphatase	Densensitization	Allergy	*Trichoplusia ni* cells			Optimized component-resolved strategy for honeybee venom allergic reactions, with a focus on developing improved diagnostic methods and improving allergenic immunotherapy.	(Stahelin, 2016; Murakami et al., 2017)

### 6.1 Liver diseases

Chronic liver disease (CLD) is a significant source of death and disability globally, with 2 million people dying from it every year (Younossi et al., 2023). In this sense, CLD is the 10th reason for mortality globally (Younossi and Henry, 2016; Heron, 2021). Besides death, CLD causes severe damage to patients’ quality of life, as well as substantial medical and economic costs (Stepanova et al., 2017). Nevertheless, various socioeconomic and medical shifts have happened worldwide during the previous 2 decades, potentially altering the impact of each CLD. BV has been demonstrated to help minimize hepatic damage, inflammation, and fibrosis (Table 6). Melittin reduces TNF release and IL-1 and IL-6 transcription in TNF-treated hepatic stellate cells (HSCs). Melittin suppresses inflammation and fibrosis in thioacetamide-induced liver fibrosis by blocking the NF-κB signaling pathway. Furthermore, they postulated that the controlled inflammatory response may impact melittin’s anti-fibrotic action in activated HSCs. They then demonstrate that melittin prevents liver failure in the D-galactosamine/LPS-induced mice liver failure model via suppressing NF-B signaling and apoptosis (Li et al., 2023). Melittin lowers the high mortality rate, alleviates hepatic pathological damage, suppresses hepatocyte apoptosis, and suppresses hepatic inflammatory responses (Jafari et al., 2024). They also observed that melittin inhibits NF-B transcription in TNF-induced liver injury. Melittin suppresses NF-κB DNA binding and promoter activity in TNF-treated hepatocytes, demonstrating that melittin prevents hepatocyte death by decreasing NF-κB activation. Recently, researchers observed that giving melittin decreased bile duct inflammation and fibrosis (Kim et al., 2015a).

**TABLE 6:** Application of bee venom in controlling the liver diseases.

Active component	Model	Outcome	Reference
Apamin	Cholestatic liver fibrosis in mice	Apamin therapy has reduced liver damage and pro-inflammatory cytokines concentrations.	Kim et al. (2017)
Bee venom	High-fat diet (HFD)-induced nonalcoholic fatty liver (NAFL) in rats	BV-treated rats had considerably decreased levels of all hepatic enzymes. Treatment resulted in the correction of adiponectin levels and substantial reductions in hepatic triglyceride and cholesterol levels.	Hanafi et al. (2018)
PLA2	Liver injury induced by acetaminophen in mice	PLA2 protects against hepatic impairment and stimulates anti-inflammatory cytokines to be generated in acetaminophen-injected rats.	Kim et al. (2014)
MEL	Acute liver failure (ALF) in mice	MEL lowered total bilirubin, ALT, AST, TNF-α, and IL-1β levels in acute liver failure mice, improving survival rates, reducing symptoms, and relieving hepatic inflammation. MEL demonstrated antioxidant and anti-inflammatory properties in LPS-stimulated RAW264.7 macrophages.	Fan et al. (2021)
phospholipase A2 (bvPLA2)	Cholestatic liver injury and fibrosis in a murine model	BvPLA2 treatment prevented hepatocyte death. Furthermore, bvPLA2 lowered cytokine production and inhibited the nuclear factor kappa-B pathway.	Kim et al. (2021b)

### 6.2 Cancer treatment

At the beginning of the 1950s, apitoxin’s influence on the colchicine-induced carcinogenic pathway was documented (Gajski et al., 2024). In turn, the study’s findings of reasons for mortality among qualified beekeepers compared with most of humanity revealed that BV had oncoprotective potential, particularly in the case of lung malignancy (Małek et al., 2023; Ali, 2024). Starting in the early 1980s, papers detailing the findings of studies on the cancer-fighting potential of BV began to appear (Table 7).

**TABLE 7:** Application of bee venom in cancer therapy.

Active component	Model	Action	Outcome	Reference
Bee venom	Human Lung cancer	Regulation of genetic transcription: Poly (ADP-ribose) polymerase (PARP), caspase-9, p53, BCL2, Box.	Apoptosis and cellular suppression.	Hwang et al. (2022)
Bee venom	Human Glioblastoma	Reduction in inflammation-promoting cytokines concentrations.	Insufficient toxic effects.	Sevin et al. (2023)
Bee venom	Pancreatic Cancer Human	Cyclin and Cyclin-dependent kinase (CDK) transcription and p53-p21 system activity can be modified.	Cell death and cellular arrest.	Zhao et al. (2022)
Melittin	Leukemia Human	Melittin-triggered decrease in mitochondria energy metabolism	Cytotoxic	Gasanoff et al. (2021)
Melittin	Gastric cancer Human	Inhibiting MMP2 and MMP9 activity leads to decreased activity in the Wnt/BMP and MMP-2 signaling pathways. Suppression of molecular adhesion.	Cytotoxicity, attachment, and infiltration suppression.	Huang et al. (2021)
Bee venom	Head and neck squamous cell carcinoma Human	Increased expression of Bax leads to reduced levels of Bcl2 and Epidermal growth factor receptor (EGFR), affecting the cell cycle.	Cisplatin has cytotoxic properties, arrests the cell cycle, and increases activity.	Grawish et al. (2020)
Bee venom, melittin	Malignant melanoma Human	Inhibits Phosphoinositide 3-kinases (PI3Ks)/AKT/mammalian target of rapamycin (mTOR) and mitogen-activated protein kinase (MAPK) processes. Increased transcription of Casp3 and Casp9	Cell death inhibits motility and colonization.	Lim et al. (2019)
Melittin	Ovarian cancer Human	Reduced amino acids in the proline/glutamine/arginine pathway. Decreasing amounts of carnitines, polyamines, ATP, and NAD+.	Cytotoxic	Alonezi et al. (2016)

MEL from *A. mellifera* is the most often used pharmacological peptide in cancer investigations. MEL may impact signaling and regulatory routes, resulting in numerous cancer-related death processes, including a decrease in the division, stimulation of the death of cells, limitation of vascular development, interruption of the cell cycle, and restriction of malignant tumor circulation, movement, the spread of cancer cells and invading forces according to available literature (Rady et al., 2017). How melittin antitumor activity has been studied, and it has been found that melittin may increase the death of hepatocellular carcinoma cells (HCC) through the CAMKII-TAK1-JNK/p38 system. In addition, melittin has been found to encourage TRAIL-resistant HCC cells to TRAIL-induced cell death, probably through stimulating the CAMKII-TAK1-JNK/p38 route and blocking the IKK-NFB signaling. The outcomes are consistent with melittin activating calcium channels, increasing Ca^2+^ concentrations inside the cells, and calcium-responsive CaMKII (Balasubramani et al., 2023). It has also been reported that BV and melittin inhibit the malignant expansion of cells by triggering caspases (3 and 9) and suppressing NF-B signaling (Ghadiri et al., 2024). It has been proven that BV inhibits proliferation and causes apoptotic cell death by activating death receptors (DR4 and DR5) (Khaliq et al., 2022). The primary reasons for cancer progression are metastasis and the infiltration of malignant cells. As a result, cancer researchers emphasize identifying the biological processes that drive cancerous cells to migrate and potential strategies to inhibit metastasis (Zuazo-Gaztelu and Casanovas, 2018).

In this context, melittin has been shown to impede *in vitro* and *in vivo* HCC cell movement via lowering Rac1-dependent processes (Haque et al., 2023). In contrast, a recent study demonstrated that combining melittin with anticancer drugs, such as temozolomide (TMZ), dramatically lowers melanoma cell growth and invasion compared to circumstances where TMZ or melittin was provided alone (Lim et al., 2019). Although MEL’s promise as a carcinoma treatment modality has long been identified, its fast breakdown in circulation and unexpected cellular lysis activities provide substantial obstacles. To address these concerns, current optimization strategies rely on nanoparticle-based melittin delivery. Nanotechnology has enabled the creation and practical testing of melittin conjugates against a variety of tumors of human kinds in preliminary studies (Singh et al., 2023; Jafari et al., 2024). To develop an effective and safe melittin delivery method that can lower hemolytic activity while maintaining cytotoxic properties. Consequently, a dual-secured nano-sting (DSNS) was created by combining zwitterionic glycol chitosan and disulfide links. At low concentrations, melittin-loaded DSNS displayed a virtually complete cytotoxic impact on numerous cancer cell types, including undamaged red blood cells (Giribaldi et al., 2021).

Only one study on cell cultivation found no statistically noteworthy cytotoxic impact on cancer cells (human glioblastoma). BV and melittin influence several intracellular pathways, and their blockage triggers apoptosis., melittin inhibits the PI3K/Akt/mTOR pathway in breast cancer cells (Pandey et al., 2023b), or liver cancer cells (Lv et al., 2023). Lim et al. found that BV and melittin suppress the PI3K/Akt/mTOR and MAPK pathways in cancerous melanoma cells (Lim et al., 2019). In two investigations on ovarian carcinoma cells, Alonezi et al. reveal a new explanation of melittin activity where compounds in the tricarboxylic acid cycle, oxidative phosphorylation, purine, and pyrimidine metabolic rate, and the arginine/proline pathways are decreased. In addition, the investigators discovered that melittin reduced carnitines, polyamines, adenosine triphosphate (ATP), and nicotinamide adenine dinucleotide (NAD+) (Alonezi et al., 2017). Erkoc et al. found that melittin induces calcium signaling apoptosis and inhibits Cyclic adenosine monophosphate (Camp) in breast cancer cells, resulting in cell death and an anti-division effect (Erkoc et al., 2022). BV also alters cell shape, resulting in DNA and protein disintegration (Jung et al., 2018). Li et al.’s study on lung cancer cells indicates that melittin boosts the generation of reactive oxygen species (ROS) and the formation of intracellular iron while affecting the activity of glutathione peroxidase 4 (GPX4); it causes mitochondrial dysfunction and death, a condition called ferroptosis (Li et al., 2022). In the past few years, some research has concentrated on the influence of BV. on cancer cell migration. The tumor mass grows complexly, with proteolytic enzymes gradually destroying the stromal tissue. Matrix metalloproteinases (MMPs) play a significant part in this process. BV inhibits the expression of MMP-2 and MMP-9 in glioblastoma cells in a dose-responsive way, but not in healthy hippocampus cells (Małek et al., 2022). It is important to note that, unlike several other pharmaceutical chemicals, BV active ingredients possess the ability to cross the blood-brain barrier, making them a potentially feasible approach for the management of CNS disorders in coming years. Additionally, research on gastric cancer cells show that melittin inhibits MMP-2 and MMP-9 function. BV and melittin have comparable effects on hepatocarcinoma cells (Mansour et al., 2021). Inhibiting angiogenesis is also linked to reduced tumor invasion. Shin et al. revealed that melittin suppresses hypoxia-inducible factor 1-alpha (HIF-1ɑ), an element that controls the production of (VEGF), a strong amplifier of vascular development. Zhang et al. found similar outcomes in non-small cell lung cancer (Zhang et al., 2015).

In another investigation on the U87 human glioblastoma line, significant cytotoxic properties began at a 5 μg/mL dose. The investigation skipped over the influence of venom on native cells. Gajski et al. found that BV had a cytotoxic impact on glioblastoma A1235 cells at 20 μg/mL (Gajski et al., 2016). However, 2 µg/mL of the crude BV caused a considerable reduction in cell survival (above 60%) in human colon cancer HCT116 cells. Another research employing the same colon cancer cell line confirmed the cytotoxic impact of BV at 1 μg/mL. Furthermore, BV had no harmful effects on FHC colon epithelial normal cells, even at a dosage of 10 μg/mL (Małek et al., 2023). The discovered BV levels that inhibit cancer cells may be inadequate to be considered in systemic-therapy without substantial adverse effects. Melittin’s great tendency to trigger hemolysis and quick metabolism might represent a hurdle to employing BV. BV has a cytotoxic impact on tumor cells at concentrations higher than 1 μg/mL (Based on the cellular lineage). To get such a BV concentration in the bodily fluids of a 75 kg individual, around 75 mg of venom would need to be administered. Nonetheless, in animal studies, a venom dosage between 0.5 and 1.0 mg/kg bw had been effectively implemented (Małek et al., 2023). Other disadvantages of systemic therapy might be eliminated by administering BV directly into the tumor. This approach has little toxicity and an excellent therapeutic index (El Bakary et al., 2020). Other disadvantages of systemic therapy might be eliminated by administering BV directly into the tumor. This approach has little toxicity and an excellent therapeutic index (Xing et al., 2003). Cell culture investigations provide for the evaluation of the molecular processes of a drug’s activity. Still, they do not answer how cancer behaves in the host’s natural environment. As a result, animal models can examine tumor growth in the body’s circumstances and the propensity to produce distant metastases. There has been significantly less animal research than *in vitro* studies on the anticancer properties of BV. In the Ehrlich ascites carcinoma mouse model, BV has been found to lower MMP-2 and MMP-9, as well as VEGF, TNF-α, and nitric oxide (NO) levels, hence decreasing tumor growth and preventing angiogenesis (El Bakary et al., 2020). Lee et al. discovered that melittin reduced VEGF levels in animals with lung cancer (Lee et al., 2017). Rocha et al. reported that melittin inhibited the formation of colorectal cancer metastases in a mouse model (Rocha et al., 2022). El-Beltagy et al. found that restoring histological abnormalities in ovarian and breast cancer rat models decreased serum MMP-1, NF-κB, and TNF-α levels (El-Beltagy et al., 2021).

Both *in vitro* and animal investigations show that BV and melittin have anticancer properties. The findings thus far put us closer to conducting clinical studies on using BV in cancer in the future (Jafari et al., 2024). Maybe additional proof of BV’s favorable benefits will lead to the initiation of human studies. As previously stated, the influence of BV on the body is multidirectional, and determining the effect of its different components on the complete human body takes extensive investigation. It is unclear if BV may achieve therapeutic quantities in tumor tissue without eliciting unpleasant consequences. It should be emphasized that in addition to the anti-cancer action, the venom has additional systemic properties that may contribute to the anticancer activity of BV.

### 6.3 In periodontal therapy

Periodontitis is a bacterial illness that damages tooth-supporting tissue, destroying ligaments and bones. Periodontitis can lead to loss of teeth, cardiovascular problems, and rheumatoid arthritis (Alkhursani et al., 2022; Atia et al., 2022; Nasser Atia et al., 2022; Atia GaN. et al., 2023). In individuals with periodontitis, osteoclasts stimulate bone resorption (Atia GaN. et al., 2023). Periodontitis is commonly treated with mechanical techniques and antibiotics. Nevertheless, there is a shortage of therapies addressing the disease’s immunological elements (Nasser Atia et al., 2022). *Porphyromonas gingivalis* is a crucial bacterium linked to chronic periodontitis commonly found in periodontal disease locations (How et al., 2016). *P. gingivalis* produces virulence-related substances to survive in the oral environment (Jia et al., 2019). These contaminants stimulate host cells, including gingival macrophages and fibroblasts, leading to inflammation in periodontal tissue and alveolar bone resorption (Xu et al., 2020; Atia GA. et al., 2023). Periodontal infection triggers inflammation that increases host immune cells and promotes the breakdown of bones through cytokines, chemokines, and attachment molecules (Cekici et al., 2014). BV inhibits *P. gingivalis*-triggered proinflammatory alveolar bone destruction *in vivo* and RANKL-associated osteoclasts development, stimulation, and metabolism *in vitro* (Gu et al., 2019).

### 6.4 Management of autoimmune diseases

Autoimmune illnesses that include rheumatoid arthritis, systemic lupus erythematosus, and multiple sclerosis have long been thought to be T helper cells (Th cells) 1-dominant; nevertheless, the importance of Th17 cells and Tregs in autoimmune diseases has only recently been discovered (Rosenblum et al., 2015). Rheumatoid arthritis is a prevalent autoimmune condition, although current treatment options are not always practical (Guo et al., 2018). BV has been utilized for decades for the management of chronic inflammatory conditions, such as rheumatoid arthritis (Wang et al., 2021a). BV’s anti-rheumatic and anti-inflammatory properties have been known for almost a century (Nainu et al., 2021).

#### 6.4.1 Rheumatoid arthritis

**FIGURE 5:**
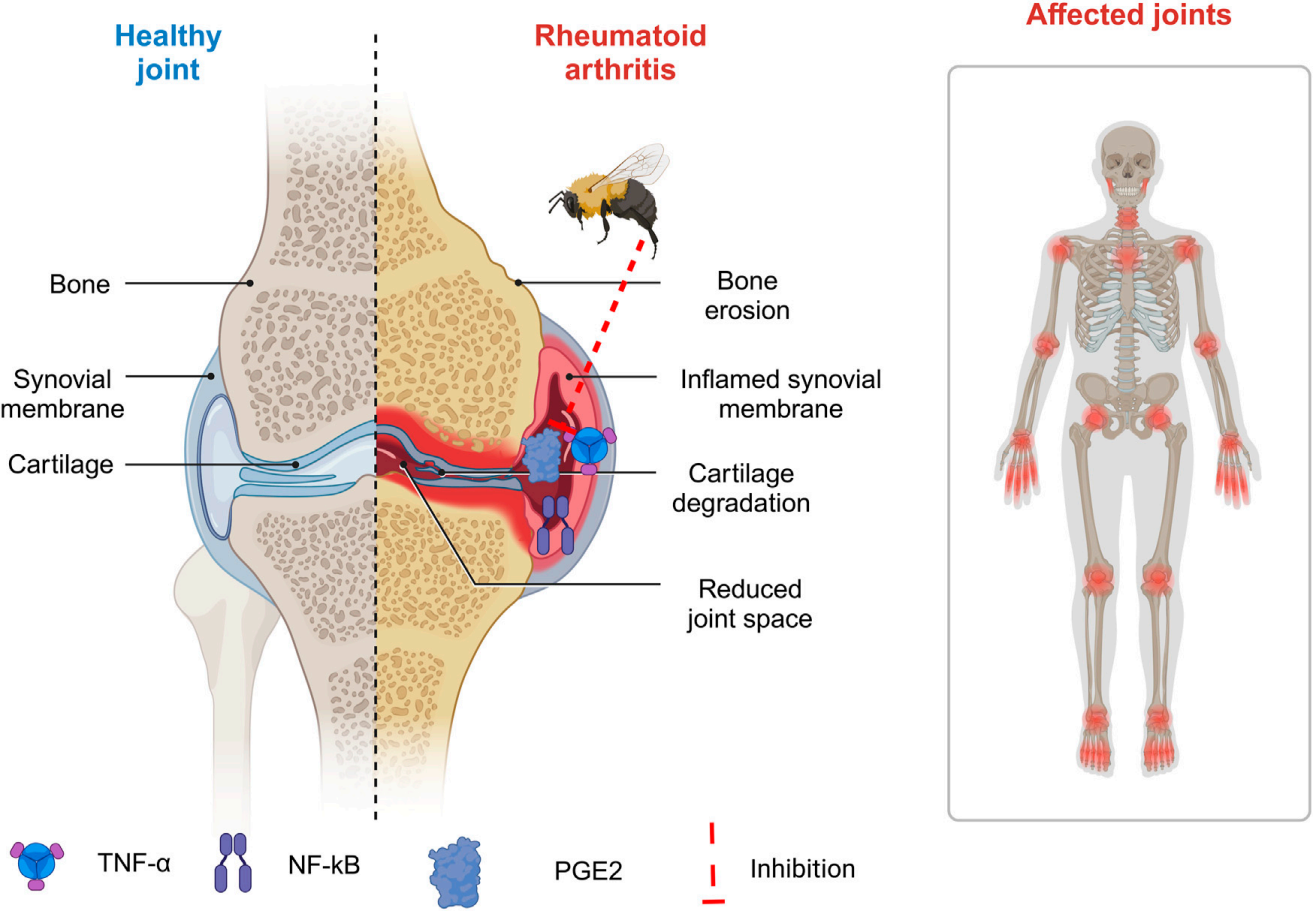
Implementation of bee venom in management of rheumatoid arthritis. (Generated by BioRender software).

Rheumatoid arthritis is an autoimmune condition characterized by synovial growth and cellular penetration. It can cause joint damage, malformation, incapacity, swelling, and pain in numerous joints (Sharif et al., 2018). Low-dose methotrexate is the most frequent therapy for rheumatoid arthritis. Methotrexate can cause hepatotoxicity, leading to insufficient drug tolerance (Wang et al., 2018). As a result, patients are more prone to pursue alternative therapies. Previous research suggests that BV injection may reduce inflammation and pain in animals with rheumatoid arthritis (Figure 5) (El-Tedawy et al., 2020). Li et al. employed Freund’s adjuvant-induced animal model to study arthritis. They discovered that treatment with BV at ST-36 inhibited Fos transcription in the superficial layer of the lumbar spinal cord, resulting in reduced paw edema and nociceptive behaviors on the injected side (Li et al., 2010). In a type II collagen-induced arthritis (CIA) model, Lee et al. discovered that BV injection treatment inhibits immunological responses. TNF-α production decreased significantly in the BV group compared to the control group, whereas IL-1β levels remained constant (Lee JaeDong et al., 2004). Another study found that rats with Cytosolic Iron-Sulfur Cluster Assembly (CIA) had much higher amounts of free radicals and protease activity than normal rats. BV injection (0.25 mg/kg) effectively modulated rheumatoid arthritis by reducing protease activity and eliminating ROS (Petronek et al., 2021). In conjunction with methotrexate, BV could boost effectiveness and have antiarthritic properties (Darwish et al., 2013).

BV injection has been shown to provide antiarthritic and antinociceptive effects in rats with arthritis, making it a potential alternative treatment for rheumatoid arthritis (RA) (El-Tedawy et al., 2020). Melittin treatment in arthritis decreases LPS-induced p50 redistribution into the nucleus, lowering inflammatory gene expression (Zhang et al., 2024). Melittin binds to p50 with high affinity (Kd = 1.2 108 M). They also looked at melittin’s anti-inflammatory properties through its associations with IKKs. Melittin suppresses TNF/LPS-induced NO and PGE2 generation in a mouse macrophage cell line and synoviocytes of rheumatoid arthritis patients (Yang et al., 2023). Additionally, they found that melittin suppresses inflammatory target gene expression and NF-κB activation through the JNK pathway (Xing et al., 2024).

#### 6.4.2 Multiple sclerosis

Multiple sclerosis is an inflammatory illness that can cause brain and spinal cord damage through inflammation of the central nervous system. The cause of multiple sclerosis is uncertain. It might be caused by a viral infection, environmental causes, or hereditary factors (Ghasemi et al., 2017). After 2000, numerous alternatives have been employed for the management of multiple sclerosis, including BV and cannabis-derived substances. The preliminary therapy with decreased nerve degeneration and glial stimulation, and cytokine production, including IFN-γ, IL-17, IL-17A, TNF-α, IL-1β, and chemokines (MCP-1 and MIP-1α). BV treatment can decrease CD4+, CD4+/Interferon-gamma (IFN-γ+), and CD4+/IL-17 + T cells while raising CD4+/Foxp3+ T cells in the nervous system and lymphatic systems of rats with acute experimental autoimmune encephalomyelitis (EAE) (Lee MJ. et al., 2016).

### 6.5 Management of neurological conditions

Among these neurological problems, neurodegenerative ailments have a substantial influence on not only people but also carers and society. The most common neurological illness is Alzheimer’s, followed by Parkinson’s and epilepsy (Nassan and Videnovic, 2022). They are a varied group with unrelenting progression, and aging is a crucial risk factor in their development. Regardless of their differences, all of these disorders are associated with intellectual disability, motor abnormalities, and behavioral abnormalities. The particular cause of neuronal degeneration and protein accumulation in many illnesses is unclear (Lamptey et al., 2022). BV products prove to be helpful in the management of neurological conditions (Table 8).

**TABLE 8:** Application of bee venom in the treatment of neurological diseases and disorders.

Active component	Disease	Model	Outcomes	Reference
Wistar rats	Spinal Cord Injury (SCI)	3–4 animals/group	BVA raised IL-10 expression at six h and decreased IL-6 production at 24 h following SCI compared to controls.	de Souza et al. (2017)
Bee venom	Intervertebral Disk Disease (IVDD)	Canines	BV injection effectively treated dogs with moderate to severe IVDD, reducing clinical rehabilitation time.	Tsai et al. (2015)
Melittin	Peripheral Neuropathy	Human	Following BVA, Patient Health Questionnaire (PHQ) scores and WHO Chemotherapy-induced peripheral neuropathy (CIPN) grades fell. VAS has also decreased.	Yoon et al. (2012)
Bee venom	Peripheral Neuropathy	Sprague–Dawley rats	BVA decreases oxaliplatin-induced acute cold allodynia by activating the serotonergic system and spinal 5-HT3 receptors.	Lee et al. (2014a)
Bee venom	Neuropathic Pain	ICR mice	BVA inhibits nociception through alpha2-adrenoceptors but not naloxone-sensitive opioid receptors.	Kwon et al. (2001)
bvPLA2	Parkinson’s disease		Injecting bvPLA2 has been demonstrated to improve motor function, reduce α-sympathy, increase microglial deactivation in the spinal cord, and normalize the M1/M2 microglial phenotypic ratio.	Ye et al. (2016a)
Bee venom/apamin	Parkinson’s disease	C57BL/6 mice	Apamin can somewhat mimic the neuroprotective properties of dopaminergic neurons.	Alvarez-Fischer et al. (2013)
Bee venom	Parkinson’s disease	C57BL/6 mice	Avoided the breakdown of tyrosine hydroxylase and reduced phospho-Jun immunoreactivity.	Doo et al. (2010)

#### 6.5.1 Parkinson’s disease

Parkinson’s disease (PD) is a degenerative movement illness that causes gradual impairment in sufferers. The illness is characterized by gradual loss of dopaminergic neurons in the substantia nigra and the appearance of Lewy bodies, which include aggregation of alpha-synuclein, extensively dispersed proteins in the cerebral tissues. Improper stimulation of microglial cells is a pathogenic marker in several neurodegenerative disorders, such as Parkinson’s (Cai et al., 2021). Several preclinical experiments examined how BV affects leukocyte migration and microglial activation in animal and cellular models (Avalo et al., 2022; Gao et al., 2023). In experimental mice models of Parkinson’s disease, BV acupuncture therapy (BVA) was shown to protect from rotenone-triggered oxidative damage, neurological inflammation, and cytotoxicity (Mi et al., 2024). Rotenone, a pesticide, may alter the pathophysiology of Parkinson’s disease (Tanner et al., 2011). BV effectively prevented dopamine depletion following rotenone injection.

Furthermore, BV treatment in PD mouse models restored locomotor activity (Morin Jr and Boudreau, 2024). The therapy reduced DNA damage and suppressed the transcription of cell death genetic factors Bax, Bcl-2, and caspase-3 in the brains of PD mice (Eleiwa et al., 2023). BV has been shown to safeguard dopamine neuronal cells against neurodegeneration in preclinical PD studies (Hassani and Esmaeili, 2024).

#### 6.5.2 Alzheimer’s disease

Alzheimer’s disease is the most prevalent neurodegenerative illness, with several pathogenic mechanisms contributing to its development (Aksoz et al., 2019). Although the cause of Alzheimer’s disease is uncertain, research suggests that inflammation could have a significant role in its progression (Twarowski and Herbet, 2023). Ye et al. discovered that bvPLA2 may successfully suppress the development of Alzheimer’s disease in transgenic mice (Ye et al., 2016b). BvPLA2 reduces Aβ buildup and improves cognitive performance in mouse brains. The study found that bvPLA2 can improve glucose metabolism and minimize neuroinflammatory reactions in the hippocampus, perhaps limiting AD development (Ye et al., 2016b). Recent research found that bvPLA2 therapy can change regulatory T-cell populations in a 3xTg-AD animal model. The investigators proposed pairing bvPLA2 therapy with Aβ vaccine therapy to slow the onset of Alzheimer’s disease while preventing harmful inflammatory responses (Baek et al., 2018).

#### 6.5.3 Amyotrophic lateral sclerosis (ALS)

ALS is a CNS illness that leads to the loss of nerve cells that control movement. ALS is characterized by aberrant buildup of mutant superoxide dismutase (SOD) 1 protein complexes (Quatorze et al.). Soares et al. characterized a mouse model of ALS with a mutant mtSOD1 gene with a Glycine to Alanine substitution (SOD1G93A) to clarify the etiology of the disease (Soares et al., 2023). Previous investigations on mutant SOD1 transgenic mice revealed pathogenic processes in motor neurons, including protein misfolding, mitochondrial malfunction, and neurofilament buildup (Zhou et al., 2023). Additionally, BV showed some potential for treating this illness. Unlike age-matched control mice, BV treatment at a particular stage of ALS development increases motor function and longevity in SOD1G93A mutant mice. This might be due to blocking active microglia, as shown in mouse models of ALS (Barreto-Núñez et al., 2024). The research found that BVA at ST36 reduces inflammation in neurons in the spinal cord of ALS animals, including TLR4, CD14, and TNF-α (Cai et al., 2015).

#### 6.5.4 Neuropathic pain

BV also helps to alleviate neuropsychiatric problems. Cold allodynia is a crucial indicator of neuropathic pain. Research suggests that BV injections may alleviate cold allodynia in mice with sciatic nerve CCIs (Er-Rouassi et al., 2023). BV, combined with peripheral β-adrenoceptor limitation, may help reduce inflammation-related pain. BVA may impact other receptors, including α4β2 nicotinic acetylcholine receptors, and relieve cold allodynia in oxaliplatin-injected mice (Yoon HeeRa et al., 2015). Numerous studies show that BV boosts Fos transcription and decreases nociceptive behavior. Coupled with BV, intrathecal clonidine-induced painkilling was much more effective (Ullah et al., 2023).

#### 6.5.5 Peripheral neuropathy

Chemotherapeutic drugs can produce peripheral neuropathy by damaging sensory and motor nerves in the peripheral nervous system. Oxaliplatin, an anticancer medication, causes neuropathic cold allodynia. A few studies have explored whether BVA can reduce mechanical allodynia (Liu F. et al., 2023; Peralta et al., 2023). Yoon et al. found that injecting DBV (0.1 mg/kg) at ST36 of the right hind leg for 2 weeks and then administering oxaliplatin (10 mg/kg) resulted in significantly decreased ipsilateral mechanical allodynia (Yoon et al., 2013). Another research study found that methysergide can inhibit BVA’s antiallodynic action linked to the serotonin system. BV has been shown to boost serotonin levels in the spinal cord and relieve cold allodynia in rats treated with oxaliplatin via activating spinal 5-HT3 receptors (Kim WooJin et al., 2016). Paclitaxel, a chemotherapy medication used to treat tumors, also caused agonizing peripheral neuropathy. In this research, BV reduced the negative consequences of paclitaxel. Additionally, BVA therapy had a strong antihyperalgesic impact at acupoint ST36 (Choi et al., 2017). Yoon et al. found that BV pharmacopuncture can lower the recommended toxicity guidelines for peripheral neuropathy (Yoon et al., 2012).

### 6.6 Male reproductive impairment

Recent research suggests that bee products may have preventative and therapeutic benefits for health, particularly infertility in men (Table 9). According to World Health Organization recommendations, 15%–25% of couples struggle to conceive. Infertility in men is triggered by changes in sperm quantity, movement, and/or shape that are found in collected samples. Recent research suggests that bee products may have preventative and therapeutic benefits for health, particularly infertility in men (Regeai et al., 2021; Suleiman et al., 2021).

**TABLE 9:** Application of bee venom in controlling the male reproductive impairment.

Active component	Model	Outcomes	Reference
Bee venom	Rabbit	Lower progesterone levels, with concomitant increase in had a boost in conception (17%) and fertility (10%).	Elkomy et al. (2021)
Bee venom	Mouse	- Restoration of normal levels of reproductive hormones and increased sperm quantity and quality.	Al-Shaeli et al. (2022)
Mellitin	Rat	Mellitin binds with proteins in tight connections between Sertoli cells.	El-Hanoun et al. (2020)
Bee venom	Rabbit	BV demonstrated a considerable favorable effect on several semen quality features, sexual behavior, antioxidant activity, lipid peroxidation indicators, and immunological response	(Al-Sayigh et al.)

According to WHO recommendations, 15%–25% of couples have difficulty conceiving. Infertility in men is triggered by changes in sperm quantity, movement, and/or shape that are found in collected samples. Infertility can be caused by various mechanisms, including steroidogenic pathway defects, pro and antioxidant activity imbalances, apoptotic pathway irregularities, pro and anti-inflammatory markers, and reactive oxygen species production (El-Hanoun et al., 2020). A study found that Iraqi bee stings protect and maintain sexual performance in mice by inhibiting Sertoli cell discharge of activin-B, which promotes spermatogonia and induces replication to create spermatocytes (Al-Sayigh et al.).

### 6.7 Skin care

BV has been utilized in medicine for therapy and as a beauty component. It has antimicrobial and anti-inflammatory properties, making it suitable for use in dermatology (Table 10). BV has a suppressive impact on *Cutibacterium acnes*. *C. acnes* is the primary cause of inflammation in acne.

**TABLE 10:** Application of bee venom in skin diseases.

Skin diseases	Model	Mechanism	References
hyperpigmentation	Hartley guinea pigs	Photoprotective, anti-melanogenic	Han et al. (2017)
Atopic dermatitis (AD)	BALB/c mouse	This medicine inhibits T cell proliferation and access and the production of Th1 and Th2 cytokines, IL-4, and IgE, which are common allergic Th2 responses in the bloodstream.	Sur et al. (2015)
*Acne Vulgaris*	HaCaT and THP-1 cells	Includes anti-inflammatory effects for Propionibacterium acnes. P. acnes secreted less IFN-γ, IL-1β, IL-8, and TNF-α after TLR2 expression was blocked.	Kim et al. (2015b)
Alopecia	C57BL/6 mouse	Increase the concentrations of the growth factors FGF-2, IGF-1R, and VEG.	Park et al. (2016)
Tissue regeneration	Mouse	Preventing inflammatory reactions may promote collagen synthesis and wound healing.	Sarhan and Azzazy (2017)
Aging marks	Human trial	Clinical investigations revealed decreases in total wrinkle area, count, and average depth, although the exact mechanism is uncertain.	Han et al. (2015)
Vitiligo	Normal human epidermal melanocyte	Triggers melanocytes to generate cAMP. The number of melanocytes increased.	Jeon et al. (2007)
Psoriasis	Twenty-five patients	TNF-α concentrations were significantly reduced than in the control group.	Eltaher et al. (2015)

An et al. found that applying BV to mice’s skin after injecting *C. acnes* into their ears decreased inflammation and levels of TNF-α and IL-1β. BV decreased the levels of TLR2 and CD14 in *C. acnes*-injected tissue (An et al., 2014). The results imply that BV can successfully cure acne. Research suggests that BV-based cosmetics can effectively treat acne vulgaris. At a dosage of 0.5 mg, concentrated BV effectively decreased C. acnes numbers (Han et al., 2013). Melittin in BV has antibacterial properties (Elieh Ali Komi et al., 2018). It effectively eliminates *S. aureus*, *Staphylococcus* epidermidis, and *Staphylococcus* pyrogens (Kim H. et al., 2019). Melittin is a poisonous peptide that destroys the bacterial cell wall (Abd El-Wahed et al., 2019). BV can treat fungal and viral skin problems. BV has been proven to have antifungal properties (Kim H. et al., 2019). The antiviral impact of BV on herpes simplex virus has been investigated. BV reduced the growth of this virus (Uddin et al., 2016). BV can block five α-reductase, which converts testosterone to dihydrotestosterone and promotes hair development, as demonstrated by studies on alopecia. Various percentages of BV (0.001%, 0.005%, and 0.01%) were compared to 2% minoxidil. BV enhanced hair development and prevented shifting from the anagen to the catagen phase. BV suppressed the levels of SRD5A2, a 5-α-reductase (Park et al., 2016).

BV may serve as a novel therapeutic for localized plaque psoriasis. Localized plaque psoriasis can be effectively treated with intradermal BV alone or oral propolis. BV includes melittin, which inhibits the production of inflammatory genes. BV suppresses COX-2 transcription, reducing the generation of inflammatory prostaglandins (Hegazi et al., 2013). BV components can have varying and sometimes contradictory immunological responses. Polypeptide adolapin suppresses prostaglandin production and inhibits the action of PLA2 and human lipoxygenase (Tusiimire et al., 2016). BV’s anti-inflammatory properties make it effective for treating atopic dermatitis. Emollients containing BV resulted in decreased eczema areas, severity indexes, and visual analog scale values compared to those without (You et al., 2016). BV’s biological activity has been utilized to cure wounds. Wound healing involves the production of TGF-β1, fibronectin, VEGF, and collagen-I.

The study on mice found that lowering wound size led to increased epithelial division. Local application of BV reduces wound size in animal models (Zhao et al., 2024). BV is used in wound dressing alongside polyvinyl alcohol and chitosan. Using 4% BV as a wound treatment in diabetic rats led to faster healing and less inflammation (Ivanov et al., 2023). Research found that combining 6% BV with chitosan improved wound healing (ÖZgenÇ and Sevin, 2023). BV was shown to enhance the growth and movement of human epidermal keratinocytes. Combining BV with hydrogel led to enhanced collagen synthesis. BV promotes wound healing through anti-inflammatory, anti-microbial, and antioxidant properties. BV is extremely successful in treating human melanoma A2058 cells. It was found that BV causes cell death by generating hydroxyl radicals (Gajski et al., 2024). In recent years, BV has been employed as an antiwrinkle treatment. Twenty-two women from South Korea used 0.006% BV serum as a cosmetic component, using 4 mL twice a day for 12 weeks. It reduced overall wrinkle area, measurement, and thickness. BV inhibits tyrosinase-related proteins, resulting in antimelanogenic action (Han et al., 2015). Han et al. found that BV reduces matrix metalloproteinase protein levels, indicating photoprotective action.

BV efficiently suppresses photoaging mechanisms and can treat photodamaged skin (Han et al., 2017). A gel with 0.06% BV did not cause photosensitive dermatitis, as proven in animals (Han et al., 2017). Melittin treats skin inflammation and protects against P. acnes-induced inflammatory reactions *in vitro* and *in vivo*. They studied the anti-inflammatory effects of melittin therapy in heat-killed P. acnes-treated HaCaT cells, which inhibited NF-B and p38 MAPK signaling. Furthermore, the anti-inflammatory efficacy of melittin was studied in a live P. acnes-induced inflammatory skin disease animal model. In the animal model, melittin-treated ears showed considerably lower swelling and granulomatous responses to P. acnes injection. Another study found that melittin reduced heat-killed P. acnes-induced apoptosis and inflammation in human THP-1 monocytic cells (Zhang et al., 2024).

### 6.8 Prevention and treatment of common cardiovascular diseases

BV can enter the body via direct stings or manual injection (Ali, 2012; Moreno and Giralt, 2015). Wehbe et al. thoroughly examine BV’s chemical makeup, biological characteristics, and mechanisms of action (Wehbe et al., 2019). Yook et al. discovered that sweet BV may impact fluctuations in heart rate, although nothing is known about its effect on CVDs (Yook et al., 2008). Wang et al. discovered that melittin, a polypeptide present in BV, can attenuate CVB3-induced myocarditis. Melittin treatment (0.1 mg/kg) lowered myocardial cell death, lowered Bax and caspase-3 expression, and increased Bcl-2. It also improved cardiac activity, as seen by echocardiography (Wang et al., 2016).

### 6.9 Management of asthma

Asthma is a potentially fatal inflammatory lung disorder with elevated CD4+ T cell counts. Research discovered that injecting BV (0.1 and 1 micro g/mL) increased regulatory T cells, inhibited cytokine production, and reduced peribronchial and perivascular inflammatory cell infiltrates in Balb/c mice with an ovalbumin-induced allergic asthma model (Reuter et al., 2024). Natural regulatory cells expressed more CD4+, CD25^+^, and FOXP3, whereas IgE levels in test animals’ blood dropped considerably (Haque et al., 2024).

### 6.10 Anti-hyperglycemic activity

Diabetes mellitus (DM) is a life-changing metabolic condition caused by abnormal insulin production, receptor activation, or both (Kharroubi and Darwish, 2015). Hyperglycemia causes excessive glycation of sugars and unbound amino groups in proteins, which alters their physical and chemical characteristics (Feng et al., 2023). BV doses (10, 20, and 40 μg/mL) were investigated *in vitro* for their effect on hemoglobin glycation when incubated with glucose. The amount of heme was evaluated by separating free amino groups with fluorescamine. The researchers discovered that BV prevents glycation-driven heme breakdown in hemoglobin. Because it has a solid antiglycation activity. It has been proposed as a natural therapy for glycation difficulties in DM (Jafari et al., 2024).

### 6.11 Protective impact against renal damage

Sepsis-induced acute kidney injury is a major worldwide health problem. Sepsis-induced acute kidney injury is a worldwide health problem. During septicemia, the endotoxin Lipopolysaccharide (LPS) causes systemic inflammation. BV, MEL, and apamin alleviated LPS-triggered severe renal damage in mice by lowering cellular oxidation damage, inflammatory processes, and cell death. MEL lowered TNF-α and IL-6 levels, decreased immune cell aggregation in the kidney, and blocked the NF-κB system. MEL decreased MDA levels, inhibited NOX4 expression, boosted Nrf2-mediated antioxidant defenses, and blocked apoptotic and necrotic processes following LPS administration. Furthermore, MEL treatment significantly prolonged the survival of mice treated with LPS (Son et al., 2007). MEL may help prevent and treat sepsis-related renal issues (Tiwari et al., 2022). In addition, MEL decreased cisplatin-induced acute kidney injury in mice by regulating M2 macrophage expression (Kim J-Y. et al., 2020).

Chronic accumulation of developing renal fibrosis produces irreversible harm to the kidneys, resulting in end-stage conditions such as renal failure. Apamin decreased renal fibrosis from unilateral ureteral obstruction *in vivo* and inhibited TGF-β1-induced activation of renal fibroblasts *in vitro*. This peptide inhibited TGF-β1/Smad2/3 and STAT3 pathways, leading to decreased inflammation, tubular atrophy, myofibroblast activation, and fibrotic gene expression (Gwon et al., 2021).

Dietary BV improved breast meat’s efficiency, yield, and nutritional value in grilled chickens. It also lowered the height of internal organs, caecal short-chain fatty acid, and ileal villus. This shows that BV might be an organic alternative to antibiotics in feed for improving animal health (Kim et al., 2018).

## 7 Clinical studies on the effect and safety of bee venom

BV has shown promising clinical effectiveness in treating a range of conditions, including asthma, allergic rhinitis, insomnia, headache, pain relief, and inflammation (Jang and Kim, 2020; Lin and Hsieh, 2020). However, its use is not without risk, as it can cause life-threatening adverse reactions (Jang and Kim, 2020). Despite this, BV is effective in treating skin varicosities veins, with potential benefits in dermatology. Further research is needed to fully understand the clinical applications and safety of BV therapy.

The safety of BV has been extensively studied, with mixed results (Bae et al., 2015; Cherniack and Govorushko, 2018; Sung et al., 2022). Cha, et al. found no significant adverse effects in mice, suggesting its potential as a safe natural antibiotic (Cha et al., 2012). However, Jang, et al. and Park, et al. both highlighted the need for caution, as BV therapy can lead to adverse events, including skin reactions and anaphylaxis (Park et al., 2019; Jang and Kim, 2020). Lee also emphasized the importance of practitioner education and qualifications in ensuring the safety of BV therapy (Lee CH. et al., 2019). Therefore, while BV may have potential as a therapeutic compound, its safety in clinical trials is still a matter of concern.

## 8 Contraindications and provisions

Many situations limit the use of BV in human therapeutics, including children under five, pregnancy, nursing, infections, post-vaccination, and medically compromised patients (Khalil et al., 2021). Systemic responses to bee stings have been seen in up to 3.4% of children and 7.5% of adults. Hence, allergy testing should be undertaken before employing BV treatment. These allergic responses can be modest, affecting only the cutaneous tissues, resulting in life-threatening anaphylaxis. Regarding venom-related allergies, venom immunotherapy is the sole medicine to prevent additional systemic sting responses (Sturm et al., 2018). To test for allergies, 0.05 mL (1.0 mg of unprocessed dried venom dissolved in 1.0 mL of normal saline is administered into the patient’s forearm flexor surface. It is infected slowly, resulting in a tiny hemispherical bleb. The absence of systemic manifestations 15–30 min after a subcutaneous administration of BV means the individual is judged not allergic to the examination. In the case of an allergic reaction, the patient should receive rapid medical assistance, which may involve the injection of adrenaline and/or dexamethasone. Since BV tolerance differs across individuals, there is no universal technique for everyone getting BV treatments. To get the finest benefits from BV treatments. All BV treatment courses should incorporate the following recommendations: (a) 2–3 mg of vitamin C daily; (b) 650 mg of acetaminophen can be administered in the event of fever and chills; (c) alcohol intake is forbidden during BV therapies; (d) an ice pack is helpful at the injection site if local reactions occur like edema; and (e) in anaphylactic shock, the patient should receive epinephrine (adrenaline) instantly and then be moved to the hospital (Bilò et al., 2019).

Advancements in controlled drug release systems and nanotechnology-based delivery methods can improve the targeted delivery of BV, increase bioavailability, and reduce side effects associated with traditional administration routes. These modern approaches allow for precise delivery of BV’s active ingredients, ensuring the right amount reaches the intended site of action. Nanotechnology-based delivery systems offer high sensitivity and specificity, allowing for rapid diagnosis and treatment of conditions like bacterial vaginosis while minimizing side effects. By optimizing therapeutic outcomes through more efficient drug delivery, these advancements have the potential to enhance the efficacy and appeal of BV therapy.

## 9 Toxicity of bee venom

Toxicology reports on BV have highlighted both its benefits and potential risks. Studies have documented adverse effects related to BV therapy, including hyperventilation, fatigue, appetite loss, extreme pain, increased bleeding risk, and vomiting (Jang and Kim, 2020; Lee et al., 2021). Furthermore, research has focused on the cellular toxicity assessment of BV microspheres in prostate cancer treatment, emphasizing the need for a thorough understanding of its impact on cellular health (El-Didamony et al., 2022a). Toxicological studies have delved into the hemolytic activity of BV in various species, such as horses, humans, sheep, and rabbits, shedding light on its cytotoxic effects. Additionally, investigations have explored the accidental ingestion of honeybee venom, emphasizing the importance of understanding the potential risks associated with exposure to BV components (de Roodt et al., 2020; Lee et al., 2021; Tanuwidjaja et al., 2021). The presence of compounds like melittin, phospholipase A2, and biogenic amines in BV has been a subject of interest, with melittin, in particular, being a major toxin known for its cytotoxic effects (Shi et al., 2022). While, BV therapy has shown promise in treating various health conditions, including inflammation and chronic illnesses, caution is warranted due to the potential for serious side effects. The use of BV products or undergoing BV therapy should be supervised by trained medical professionals to mitigate risks. Research has highlighted the dual nature of BV, with compounds like melittin, and apamin exhibiting anti-inflammatory and pain-relieving properties, but also carrying the risk of irritation and allergic reactions in sensitized individuals (Jang and Kim, 2020; Ullah et al., 2023).

## 10 Scaffolds based delivery for bee venom delivery

Biological preparations comprise substances from vegetation and animals; some of these products possess an extensive record of use, while others have been found in recent years (Giannenas et al., 2020). For instance, the therapeutic efficacy of natural extracts and components has been hampered by a variety of issues, notably an absence of targeting capability and low bioavailability. Scientists recommended employing scaffolds infused with BV to address its disadvantages and boost its physiological and medicinal benefits (Atia GA. et al., 2023; Liu H. et al., 2023; Atia et al., 2024; El-Nablaway et al., 2024). Despite advances in cancer treatment, the illness remains the leading cause of death globally. BV has a variety of biological characteristics and has been shown *in vitro* to be cytotoxic against many cancer cell lines. However, its use in humans remains challenging because to allergic responses, discomfort at administration sites, and other serious toxic events including hemolysis, nonspecific cytotoxic effects, itching feeling, and hypersensitivity during therapeutic therapy. BV-loaded nanoliposomes (BV-NLs) were synthesized and characterized, and their anticancer efficacy was tested *in vitro* against the HepG-2, MCF-7, and HCT-116 cell lines. They outperformed raw BV in terms of anticancer efficacy against the 3 cell lines evaluated. It demonstrated preferential cytotoxicity and was more potent against the HCT-116 cell line, with an IC50 of 4.16 μg/mL. BV-NLs also regulate the mRNA transcription of apoptosis genes (Abd El-Gawad et al., 2023). Another study used the freeze-thawing process to create various formulations of polyvinyl alcohol (PVA) and chitosan (Ch) hydrogel wound dressings incorporating BV. It was more swollen, flexible, and elastic than the other formulations. Furthermore, it demonstrated anti-inflammatory properties equivalent to diclofenac gel, commonly used anti-inflammation medicine. Meanwhile, wound tissues coated with this mixture demonstrated greater hydroxyproline and glutathione amounts and decreased IL-6 levels relative to the control (Amin and Abdel-Raheem, 2014). The BV or its contents (e.g., melittin) were intensively explored as prospective therapy and antagonists of tumor types; they shown numerous prospective biological anticancer processes (Borojeni et al., 2020; Lebel et al., 2021; Zein et al., 2024). Nanotechnology has become widely used in most medical disciplines, with nanomaterials being used as antimicrobial, cancer-fighting, or loaded with biologically active drugs/compounds that improve their solubility, stability, performance, and transport to the human body (Alkhursani et al., 2022; El-Nablaway et al., 2024). In this line, nano chitosan produced from Fusarium oxysporum-grown mycelia had been utilized to transport BV. The *in vitro* antitumor capacity evaluation against HeLa cervical carcinoma indicated that they all displayed significant dose-related cancer-fighting properties, with BV/NFC nanoconjugates being the most efficient. The fluorescent labeling of these cells demonstrated the presence of initial apoptosis, subsequent apoptosis, and additional necrosis indicators, and their increase with contact extension (Alalawy et al., 2020). Due to the increasing prevalence of adaptive human microorganisms across the world, antimicrobial resistance has become one of the most pressing medical concerns. Chitosan synthesized from dead bee exoskeletons laden with BV has previously been developed and evaluated against eight prevalent human infections (two fungal and six bacterial strains) as well as two cancer cell lines. It has increased antibacterial action against six prevalent human diseases, including Gram-positive and Gram-negative bacterial and fungal strains. Although hazardous at high doses, the nanoparticles demonstrated exceptional activity against the human colon cancer cell line (Caco2 ATCC ATP-37) and human liver cancer cell line (HepG2 ATCC HB-8065), eliminating around 72% of cancer cells (Sharaf et al., 2024b). In another work, BV-loaded chitosan nanoparticles (ChNPs) had weak anti-MERS-COV activity (SI = 4.6), whereas ChNPs had considerable anti-MERS-COV activity (SI = 8.6). Meanwhile. It showed significant anti-MERS-COV activity (SI = 12.1). Furthermore, the synthesized platform demonstrated better antibacterial action against both Gram-positive and Gram-negative bacteria when contrasted with ChNPs, BV, or the used experimental medication (Elnosary et al., 2023).

## 11 Conclusion

BV is a complex combination of compounds widely researched because of their biological effects. Proteins and peptides comprise most of its content, with additional molecules present in small amounts. Between its constituents, melittin is the most prevalent and researched constituent of BV, after which comes PLA2, an enzyme that is regarded (along with histamine) as the primary allergen of BV. Furthermore, additional investigation is needed, particularly on BV’s less significant elements. Numerous physiological characteristics have been documented. However, a study has focused chiefly on anti-inflammation and immune-modulating benefits. However, further *in vitro* and *in vivo* research is required to understand the BV action mechanisms better. Due to its beneficial effects on certain conditions, including musculoskeletal and neurological ailments, BV’s primary applications are therapeutic. Nowadays, attempts are being made to develop safer dosages and methods and new trends like novel delivery technologies to reduce undesirable effects. In addition, the study advances the comprehension of the pathways of BV ingredients and evaluates their medicinal applicability.

## Figures and Tables

**Table:** 

**ACHE**	Acetylcholinesterase
**AD**	Alzheimer’s disease
**ALF**	Acute liver failure
**ALS**	Amyotrophic lateral sclerosis
**ALS**	Amyotrophic lateral sclerosis
**ALT**	Alanine aminotransferase
**Amps**	Antimicrobial peptides
**AOA**	Antioxidant activity
**AST**	Aspartate transaminase
**ATF3**	Activating transcription factor 3
**BV**	Bee venom
**BV-nls**	BV-loaded nanoliposomes
**Bvpla2**	Bee venom phosphao lipase A2
**Camp**	Cyclic adenosine monophosphate
**C-AMP**	Cyclic adenosine monophosphate
**CDK**	Cyclin-dependent kinase
**C-GMP**	Cyclic guanosine monophosphate
**Ch**	Chitosan
**ChNPs**	Chitosan nanoparticles
**CIA**	Collagen-induced arthritis
**CK-MB**	Creatine Kinase MB
**CLD**	Chronic liver disease
**COX-2**	Cyclooxygenase-2
**CTNI**	Cardiac Troponin I
**DM**	Diabetes mellitus
**DPIV**	Dipeptidyl peptidase IV
**DSNS**	Dual-secured nano-sting
**EAE**	Experimental autoimmune encephalomyelitis
**ECM**	Extracellular matrix
**Foxp3**	Forkhead box P3
**GSH**	Glutathione
**GST**	Glutathione S-transferase
**HCC**	Hepatocellular carcinoma cells
**HCV**	Hepatitis C virus
**HFD**	High-fat diet
**HIF-1ɑ**	Hypoxia-inducible factor 1-alpha
**HIV**	Human immunodeficiency virus
**HPV**	Papillomaviruse
**Hscs**	Hepatic stellate cells
**HSV**	Herpes simplex virus
**IFN-γ**	Interferon-gamma
**IL-8**	Interleukin-8
**iNOS**	Inducible nitric oxide synthase
**LDH**	Lactate Dehydrogenase
**LPS**	Lipopolysaccharides
**MAPK**	Mitogen-activated protein kinase
**MCD**	Mast cell degranulating
**MDA**	Malondialdehyde
**MEL**	Melittin
**MIC**	Minimum inhibitory concentration
**MMPS**	Matrix metalloproteinases
**NAD** ^**+**^	Nicotinamide adenine dinucleotide
**NAFL**	Nonalcoholic fatty liver
**NF-κB**	Nuclear factor kappa B
**NO**	Nitric oxide
**PDGF-BB**	Platelet derived growth factor-BB
**PI3Ks**	Phosphoinositide 3-kinases
**PLA2**	Phospholipase A2
**PVA**	Polyvinyl alcohol
**RA**	Rheumatoid arthritis
**RANKL**	Receptor activator of nuclear factor kappa-Β ligand
**RLPP**	Recalcitrant localized plaque psoriasis
**RNA**	Ribonucleic acid
**SK**	Small-conductance calcium-activated potassium
**SOD**	Superoxide dismutase
**STAT3**	Signal transducer and activator of transcription 3
**TAC**	Total antioxidant capacity
**TBARS**	Thiobarbituric acid–reactive substances
**TGF-β**	Transforming growth factor beta
**Th cells**	T helper cells
**TLR4**	Toll-like receptor 4
**TMDs**	Tempormandibular disorders
**TMZ**	Temozolomide
**TNF-α**	Tumor necrosis factor-α
**Tregs**	Regulatory T-cells
**UCF**	Unicellular fungal
**VEGF**	Vascular endothelial growth factor

## References

[B1] Abdel N. Atia G. Shalaby H. K. Zehravi M. Ghobashy M. M. Ahmad Z. (2022). Locally applied repositioned hormones for oral bone and periodontal tissue engineering: a narrative review. Polymers 14 (14), 2964. 10.3390/polym14142964 35890740 PMC9319147

[B2] Abd El-Aziz A. H. El-Kasrawy N. I. Abd El-Hack M. E. Swelum A. A. Suliman G. Tufarelli V. (2024). Impact of bee venom supplement on productive performance, health status and economics of weaned male rabbits: considering breed and dosage factors. J. Animal Physiology Animal Nutr. 104, 186–195. 10.1111/jpn.13227 38311831

[B3] Abd El-Gawad A. Kenawy M. A. El-Messery T. M. Hassan M. E. El-Nekeety A. A. Abdel-Wahhab M. A. (2023). Fabrication and characterization of bee venom-loaded nanoliposomes: enhanced anticancer activity against different human cancer cell lines via the modulation of apoptosis-related genes. J. Drug Deliv. Sci. Technol. 84, 104545. 10.1016/j.jddst.2023.104545

[B4] Abd El-Wahed A. A. Khalifa S. a. M. Sheikh B. Y. Farag M. A. Saeed A. Larik F. A. (2019). Bee venom composition: from chemistry to biological activity. Stud. Nat. Prod. Chem. 60, 459–484. 10.1016/b978-0-444-64181-6.00013-9

[B5] Ahmed-Farid O. A. Taha M. Bakeer R. M. Radwan O. K. Hendawy H. a. M. Soliman A. S. (2021). Effects of bee venom and dopamine-loaded nanoparticles on reserpine-induced Parkinson’s disease rat model. Sci. Rep. 11 (1), 21141. 10.1038/s41598-021-00764-y 34707203 PMC8551202

[B6] Aksoz E. Gocmez S. S. Sahin T. D. Aksit D. Aksit H. Utkan T. (2019). The protective effect of metformin in scopolamine-induced learning and memory impairment in rats. Pharmacol. Rep. 71 (5), 818–825. 10.1016/j.pharep.2019.04.015 31382167

[B7] Alalawy A. I. El Rabey H. A. Almutairi F. M. Tayel A. A. Al-Duais M. A. Zidan N. S. (2020). Effectual anticancer potentiality of loaded bee venom onto fungal chitosan nanoparticles. Int. J. Polym. Sci. 2020, 1–9. 10.1155/2020/2785304

[B8] Ali A. M. H. (2024). Advancements in bee venom bioactivities for current therapeutic applications: a Review. Adv. Life Sci. 11 (1), 49–55.

[B9] Ali E. M. (2014). Contributions of some biological activities of honey bee venom. J. Apic. Res. 53 (4), 441–451. 10.3896/ibra.1.53.4.13

[B10] Ali M. (2012). Studies on bee venom and its medical uses. Int. J. Adv. Res. Technol. 1 (2), 69–83.

[B11] Alkhursani S. A. Ghobashy M. M. Al-Gahtany S. A. Meganid A. S. Abd El-Halim S. M. Ahmad Z. (2022). Application of nano-inspired scaffolds-based biopolymer hydrogel for bone and periodontal tissue regeneration. Polymers 14 (18), 3791. 10.3390/polym14183791 36145936 PMC9504130

[B12] Alonezi S. Tusiimire J. Wallace J. Dufton M. J. Parkinson J. A. Young L. C. (2016). Metabolomic profiling of the effects of melittin on cisplatin resistant and cisplatin sensitive ovarian cancer cells using mass spectrometry and biolog microarray technology. Metabolites 6 (4), 35. 10.3390/metabo6040035 27754384 PMC5192441

[B13] Alonezi S. Tusiimire J. Wallace J. Dufton M. J. Parkinson J. A. Young L. C. (2017). Metabolomic profiling of the synergistic effects of melittin in combination with cisplatin on ovarian cancer cells. Metabolites 7 (2), 14. 10.3390/metabo7020014 28420117 PMC5487985

[B14] Al-Sayigh M. A. Al-Mallah K. H. Abdul-Rasoul E. M. Al-Sadi H. I. (2024). Effect of bee venom on sexual efficiency in normal and hydrogen peroxide treated adult male rats.

[B15] Al-Shaeli S. J. J. Hussen T. J. Ethaeb A. M. (2022). Effect of honey bee venom on the histological changes of testes and hormonal disturbance in diabetic mice. Veterinary World 15 (9), 2357–2364. 10.14202/vetworld.2022.2357-2364 36341058 PMC9631373

[B16] Alvarez-Fischer D. Noelker C. Vulinović F. Grünewald A. Chevarin C. Klein C. (2013). Bee venom and its component apamin as neuroprotective agents in a Parkinson disease mouse model. PLoS One 8 (4), e61700. 10.1371/journal.pone.0061700 23637888 PMC3630120

[B17] Amin M. A. Abdel-Raheem I. T. (2014). Accelerated wound healing and anti-inflammatory effects of physically cross linked polyvinyl alcohol–chitosan hydrogel containing honey bee venom in diabetic rats. Archives pharmacal Res. 37, 1016–1031. 10.1007/s12272-013-0308-y 24293065

[B18] An H. J. Kim J. Y. Kim W. H. Gwon M. G. Gu H. M. Jeon M. J. (2018). Therapeutic effects of bee venom and its major component, melittin, on atopic dermatitis *in vivo* and *in vitro* . Br. J. Pharmacol. 175 (23), 4310–4324. 10.1111/bph.14487 30187459 PMC6240132

[B19] An H.-J. Lee W.-R. Kim K.-H. Kim J.-Y. Lee S.-J. Han S.-M. (2014). Inhibitory effects of bee venom on Propionibacterium acnes-induced inflammatory skin disease in an animal model. Int. J. Mol. Med. 34 (5), 1341–1348. 10.3892/ijmm.2014.1933 25215662

[B20] Annila I. T. Karjalainen E. S. Annila P. A. Kuusisto P. A. (1996). Bee and wasp sting reactions in current beekeepers. Ann. Allergy, Asthma and Immunol. 77 (5), 423–427. 10.1016/S1081-1206(10)63342-X 8933782

[B21] Aparna N. (2020). Production technology of royal jelly and bee venom. Econ. Entomol., 115–132.

[B22] Atia G. A. Shalaby H. K. Roomi A. B. Ghobashy M. M. Attia H. A. Mohamed S. Z. (2023a). Macro, micro, and nano-inspired bioactive polymeric biomaterials in therapeutic, and regenerative orofacial applications. Drug Des. Dev. Ther. 17, 2985–3021. 10.2147/DDDT.S419361 PMC10543943 37789970

[B23] Atia G. a. N. Mohamed S. Z. Halim H. A. Ghobashy M. M. Foda T. Shalaby H. K. (2024). Advances in Bioceramic silicates for therapeutic, and regenerative Dentofacial reconstruction. Ceram. Int. 50, 22184–22208. 10.1016/j.ceramint.2024.04.035

[B24] Atia G. a. N. Shalaby H. K. Ali N. G. Morsy S. M. Ghobashy M. M. Attia H. a. N. (2023b). New challenges and prospective applications of three-dimensional bioactive polymeric hydrogels in oral and craniofacial tissue engineering: a narrative review. Pharmaceuticals 16 (5), 702. 10.3390/ph16050702 37242485 PMC10224377

[B25] Atia G. a. N. Shalaby H. K. Zehravi M. Ghobashy M. M. Attia H. a. N. Ahmad Z. (2022). Drug-loaded chitosan scaffolds for periodontal tissue regeneration. Polymers 14 (15), 3192. 10.3390/polym14153192 35956708 PMC9371089

[B26] Avalo Z. Barrera M. C. Agudelo-Delgado M. Tobón G. J. Cañas C. A. (2022). Biological effects of animal venoms on the human immune system. Toxins 14 (5), 344. 10.3390/toxins14050344 35622591 PMC9143185

[B27] Bae Y. H. Lee C. H. Kim H. S. Kim H. S. Suh C. Y. Kim N. H. (2015). Stability and safety of bee venom with and without additives. J. Acupunct. Res. 32 (3), 127–133. 10.13045/acupunct.2015045

[B28] Baek H. Lee C.-J. Choi D. B. Kim N.-S. Kim Y.-S. Ye Y. J. (2018). Bee venom phospholipase A2 ameliorates Alzheimer's disease pathology in Aβ vaccination treatment without inducing neuro-inflammation in a 3xTg-AD mouse model. Sci. Rep. 8 (1), 17369. 10.1038/s41598-018-35030-1 30478329 PMC6255868

[B29] Balasubramani G. Muthu M. Gopal J. Chun S. (2023). A review on the impact of TRAIL on cancer signaling and targeting via phytochemicals for possible cancer therapy. Int. J. Biol. Macromol. 253, 127162. 10.1016/j.ijbiomac.2023.127162 37788732

[B30] Banks B. E. Dempsey C. E. Vernon C. A. Warner J. A. Yamey J. (1990). Anti-inflammatory activity of bee venom peptide 401 (mast cell degranulating peptide) and compound 48/80 results from mast cell degranulation *in vivo* . Br. J. Pharmacol. 99 (2), 350–354. 10.1111/j.1476-5381.1990.tb14707.x 2328399 PMC1917405

[B31] Barreto‐Núñez R. Béland L. C. Boutej H. Picher‐Martel V. Dupré N. Barbeito L. (2024). Chronically activated microglia in ALS gradually lose their immune functions and develop unconventional proteome. Glia 72, 1319–1339. 10.1002/glia.24531 38577970

[B32] Bava R. Castagna F. Musella V. Lupia C. Palma E. Britti D. (2023). Therapeutic use of bee venom and potential applications in veterinary medicine. Veterinary Sci. 10 (2), 119. 10.3390/vetsci10020119 PMC9965945 36851423

[B33] Bellik Y. (2015). Bee venom: its potential use in alternative medicine. Anti-infective agents 13 (1), 3–16. 10.2174/2211352513666150318234624

[B34] Berhe H. Kumar Cinthakunta Sridhar M. Zerihun M. Qvit N. (2024). The potential use of peptides in the fight against chagas disease and leishmaniasis. Pharmaceutics 16 (2), 227. 10.3390/pharmaceutics16020227 38399281 PMC10892537

[B35] Bilò M. B. Pravettoni V. Bignardi D. Bonadonna P. Mauro M. Novembre E. (2019). Hymenoptera venom allergy: management of children and adults in clinical practice. J. investigational allergology Clin. Immunol. 29 (3), 180–205. 10.18176/jiaci.0310 30183660

[B36] Bindlish A. Sawal A. (2024). Bee sting venom as a viable therapy for breast cancer: a review article. Cureus 16 (2), e54855. 10.7759/cureus.54855 38533165 PMC10964279

[B37] Blank S. Seismann H. Bockisch B. Braren I. Cifuentes L. Mcintyre M. (2010). Identification, recombinant expression, and characterization of the 100 kDa high molecular weight Hymenoptera venom allergens Api m 5 and Ves v 3. J. Immunol. 184 (9), 5403–5413. 10.4049/jimmunol.0803709 20348419

[B38] Borojeni S. K. Zolfagharian H. Babaie M. Javadi I. (2020). Cytotoxic effect of bee (*A. mellifera*) venom on cancer cell lines. J. Pharmacopuncture 23 (4), 212–219. 10.3831/KPI.2020.23.4.212 33408897 PMC7772077

[B39] Cai H.-Y. Fu X.-X. Jiang H. Han S. (2021). Adjusting vascular permeability, leukocyte infiltration, and microglial cell activation to rescue dopaminergic neurons in rodent models of Parkinson’s disease. npj Parkinson's Dis. 7 (1), 91. 10.1038/s41531-021-00233-3 34625569 PMC8501121

[B40] Cai M. Choi S.-M. Yang E. J. (2015). The effects of bee venom acupuncture on the central nervous system and muscle in an animal hSOD1G93A mutant. Toxins 7 (3), 846–858. 10.3390/toxins7030846 25781653 PMC4379529

[B41] Carpena M. Nuñez-Estevez B. Soria-Lopez A. Simal-Gandara J. (2020). Bee venom: an updating review of its bioactive molecules and its health applications. Nutrients 12 (11), 3360. 10.3390/nu12113360 33142794 PMC7693387

[B42] Cekici A. Kantarci A. Hasturk H. Van Dyke T. E. (2014). Inflammatory and immune pathways in the pathogenesis of periodontal disease. Periodontology 64 (1), 57–80. 10.1111/prd.12002 PMC4500791 24320956

[B43] Ceremuga M. Stela M. Janik E. Gorniak L. Synowiec E. Sliwinski T. (2020). Melittin—a natural peptide from bee venom which induces apoptosis in human leukaemia cells. Biomolecules 10 (2), 247. 10.3390/biom10020247 32041197 PMC7072249

[B44] Cha C.-N. Lee Y.-E. Kim S. Lee H. J. (2012). Evaluation for the safety of bee venom in ICR mice. J. Biomed. Res. 13 (1), 65–70. 10.12729/jbr.2012.13.1.65

[B45] Chen M. Aoki-Utsubo C. Kameoka M. Deng L. Terada Y. Kamitani W. (2017). Broad-spectrum antiviral agents: secreted phospholipase A2 targets viral envelope lipid bilayers derived from the endoplasmic reticulum membrane. Sci. Rep. 7 (1), 15931. 10.1038/s41598-017-16130-w 29162867 PMC5698466

[B46] Cherniack E. P. Govorushko S. (2018). To bee or not to bee: the potential efficacy and safety of bee venom acupuncture in humans. Toxicon 154, 74–78. 10.1016/j.toxicon.2018.09.013 30268393

[B47] Choi G.-M. Lee B. Hong R. Park S.-Y. Cho D.-E. Yeom M. (2021). Bee venom phospholipase A2 alleviates collagen-induced polyarthritis by inducing Foxp3+ regulatory T cell polarization in mice. Sci. Rep. 11 (1), 3511. 10.1038/s41598-021-82298-x 33568685 PMC7876016

[B48] Choi J. Jeon C. Lee J. H. Jang J. U. Quan F. S. Lee K. (2017). Suppressive effects of bee venom acupuncture on paclitaxel-induced neuropathic pain in rats: mediation by spinal α₂-adrenergic receptor. Toxins 9 (11), 351. 10.3390/toxins9110351 29088102 PMC5705966

[B49] Chu S.-T. Cheng H.-H. Huang C.-J. Chang H.-C. Chi C.-C. Su H.-H. (2007). Phospholipase A2-independent Ca2+ entry and subsequent apoptosis induced by melittin in human MG63 osteosarcoma cells. Life Sci. 80 (4), 364–369. 10.1016/j.lfs.2006.09.024 17054998

[B50] Cummings J. Lee G. Ritter A. Zhong K. (2018). Alzheimer's disease drug development pipeline: 2018. Alzheimer's Dementia Transl. Res. Clin. Interventions 4, 195–214. 10.1016/j.trci.2018.03.009 PMC6021548 29955663

[B51] Dadar M. Shahali Y. Chakraborty S. Prasad M. Tahoori F. Tiwari R. (2019). Antiinflammatory peptides: current knowledge and promising prospects. Inflamm. Res. 68, 125–145. 10.1007/s00011-018-1208-x 30560372

[B52] Darwish S. F. El-Bakly W. M. Arafa H. M. El-Demerdash E. (2013). Targeting TNF-α and NF-κB activation by bee venom: role in suppressing adjuvant induced arthritis and methotrexate hepatotoxicity in rats. PLoS One 8 (11), e79284. 10.1371/journal.pone.0079284 24278124 PMC3835890

[B53] Değer O. Yiğit E. Korkmaz K. Aygün P. Asghari A. Çakıroğlu K. A. (2023). Protective effect of bee products against oxidative damage in erythrocytes. Gümüşhane Üniversitesi Sağlık Bilim. Derg. 12 (1), 167–174. 10.37989/gumussagbil.1095925

[B54] De Graaf D. C. Brochetto Braga M. R. De Abreu R. M. M. Blank S. Bridts C. H. De Clerck L. S. (2021). Standard methods for *Apis mellifera* venom research. J. Apic. Res. 60 (4), 1–31. 10.1080/00218839.2020.1801073

[B55] De Roodt A. R. Lanari L. C. Lago N. R. Bustillo S. Litwin S. Morón-Goñi F. (2020). Toxicological study of bee venom (*Apis mellifera* mellifera) from different regions of the province of Buenos Aires, Argentina. Toxicon 188, 27–38. 10.1016/j.toxicon.2020.09.014 33007351

[B56] De Souza R. N. Silva F. K. De Medeiros M. A. (2017). Bee venom acupuncture reduces interleukin-6, increases interleukin-10, and induces locomotor recovery in a model of spinal cord compression. J. Acupunct. meridian Stud. 10 (3), 204–210. 10.1016/j.jams.2017.04.003 28712480

[B57] Dinata R. Arati C. Manikandan B. Abinash G. Nisa N. Bhanushree B. (2023). Pharmacological and therapeutic potential of honey bee antimicrobial peptides: bee antimicrobial peptides. Indian J. Biochem. Biophysics (IJBB) 60 (5), 365–384.

[B58] Dinu M. Tatu A. L. Cocoș D. I. Nwabudike L. C. Chirilov A. M. Stefan C. S. (2024). Natural sources of therapeutic agents used in skin conditions. Life 14 (4), 492. 10.3390/life14040492 38672762 PMC11051086

[B59] Djahonkulovna S. L. (2023). Analysis of modern pathomorphological data obtained in experimental studies. Tex. J. Eng. Technol. 20, 38–44.

[B60] Doo A.-R. Kim S.-T. Kim S.-N. Moon W. Yin C. S. Chae Y. (2010). Neuroprotective effects of bee venom pharmaceutical acupuncture in acute 1-methyl-4-phenyl-1, 2, 3, 6-tetrahydropyridine-induced mouse model of Parkinson's disease. Neurological Res. 32 (Suppl. 1), 88–91. 10.1179/016164109X12537002794282 20034453

[B61] Doo K.-H. Lee J.-H. Cho S.-Y. Jung W.-S. Moon S.-K. Park J.-M. (2015). A prospective open-label study of combined treatment for idiopathic Parkinson's disease using acupuncture and bee venom acupuncture as an adjunctive treatment. J. Altern. Complementary Med. 21 (10), 598–603. 10.1089/acm.2015.0078 26230989

[B62] Dos Santos-Pinto J. R. A. Perez-Riverol A. Lasa A. M. Palma M. S. (2018). Diversity of peptidic and proteinaceous toxins from social Hymenoptera venoms. Toxicon 148, 172–196. 10.1016/j.toxicon.2018.04.029 29715467

[B63] Duché G. Sanderson J. M. (2024). The chemical reactivity of membrane lipids. Chem. Rev. 124 (6), 3284–3330. 10.1021/acs.chemrev.3c00608 38498932 PMC10979411

[B64] Duda G. N. Geissler S. Checa S. Tsitsilonis S. Petersen A. Schmidt-Bleek K. (2023). The decisive early phase of bone regeneration. Nat. Rev. Rheumatol. 19 (2), 78–95. 10.1038/s41584-022-00887-0 36624263

[B65] El Bakary N. M. Alsharkawy A. Z. Shouaib Z. A. Barakat E. M. S. (2020). Role of bee venom and melittin on restraining angiogenesis and metastasis in γ-irradiated solid ehrlich carcinoma-bearing mice. Integr. cancer Ther. 19, 1534735420944476. 10.1177/1534735420944476 32735464 PMC7401046

[B66] El-Beltagy A. E.-F. B. M. Elsyyad H. I. H. Abdelaziz K. K. Madany A. S. Elghazaly M. M. (2021). Therapeutic role of Annona muricata fruit and bee venom against MNU-induced breast cancer in pregnant rats and its complications on the ovaries. Breast Cancer Targets Ther. 13, 431–445. 10.2147/BCTT.S306971 PMC8275159 34267553

[B67] El-Didamony S. E. Amer R. I. El-Osaily G. H. (2022a). Formulation, characterization and cellular toxicity assessment of a novel bee-venom microsphere in prostate cancer treatment. Sci. Rep. 12 (1), 13213. 10.1038/s41598-022-17391-w 35918370 PMC9346107

[B68] El-Didamony S. E. Kalaba M. H. El-Fakharany E. M. Sultan M. H. Sharaf M. H. (2022b). Antifungal and antibiofilm activities of bee venom loaded on chitosan nanoparticles: a novel approach for combating fungal human pathogens. World J. Microbiol. Biotechnol. 38 (12), 244. 10.1007/s11274-022-03425-y 36280608 PMC9592658

[B69] Eleiwa N. Z. H. Ali M. a.-A. Said E. N. Metwally M. M. M. Abd-Elhakim Y. M. (2023). Bee venom (*Apis mellifera* L.) rescues zinc oxide nanoparticles induced neurobehavioral and neurotoxic impact via controlling neurofilament and GAP-43 in rat brain. Environ. Sci. Pollut. Res. 30 (38), 88685–88703. 10.1007/s11356-023-28538-1 PMC10412495 37442924

[B70] El Gendy A. Saber M. M. Daoud E. M. Abdel-Wahhab K. G. Abd El-Rahman E. Hegazi A. G. (2017). Role of bee venom acupuncture in improving pain and life quality in Egyptian chronic low back pain patients. J. Appl. Pharm. Sci. 7 (8), 168–174. 10.7324/JAPS.2017.70823

[B71] El-Hanoun A. El-Komy A. El-Sabrout K. Abdella M. (2020). Effect of bee venom on reproductive performance and immune response of male rabbits. Physiology Behav. 223, 112987. 10.1016/j.physbeh.2020.112987 32492496

[B72] Elieh Ali Komi D. Shafaghat F. Zwiener R. D. (2018). Immunology of bee venom. Clin. Rev. allergy and Immunol. 54, 386–396. 10.1007/s12016-017-8597-4 28105558

[B73] Elkomy A. El‐Hanoun A. Abdella M. El‐Sabrout K. (2021). Improving the reproductive, immunity and health status of rabbit does using honey bee venom. J. Animal Physiology Animal Nutr. 105 (5), 975–983. 10.1111/jpn.13552 33856075

[B74] El Mehdi I. Falcão S. I. Boujraf S. Mustapha H. Campos M. G. Vilas-Boas M. (2022). Analytical methods for honeybee venom characterization. J. Adv. Pharm. Technol. Res. 13 (3), 154–160. 10.4103/japtr.japtr_166_21 35935688 PMC9355049

[B75] El-Nablaway M. Rashed F. Taher E. S. Foda T. Abdeen A. Abdo M. (2024). Prospectives and challenges of nano-tailored biomaterials-assisted biological molecules delivery for tissue engineering purposes. Life Sci. 349, 122671. 10.1016/j.lfs.2024.122671 38697279

[B76] Elnosary M. E. Aboelmagd H. A. Habaka M. A. Salem S. R. El-Naggar M. E. (2023). Synthesis of bee venom loaded chitosan nanoparticles for anti-MERS-COV and multi-drug resistance bacteria. Int. J. Biol. Macromol. 224, 871–880. 10.1016/j.ijbiomac.2022.10.173 36283561 PMC9595425

[B77] El-Seedi H. Abd El-Wahed A. Yosri N. Musharraf S. G. Chen L. Moustafa M. (2020). Antimicrobial properties of *Apis mellifera*’s bee venom. Toxins 12 (7), 451. 10.3390/toxins12070451 32664544 PMC7404974

[B78] Eltaher S. Mohammed G. F. Younes S. Elakhras A. (2015). Efficacy of the apitherapy in the treatment of recalcitrant localized plaque psoriasis and evaluation of tumor necrosis factor-alpha (TNF-α) serum level: a double-blind randomized clinical trial. J. Dermatological Treat. 26 (4), 335–339. 10.3109/09546634.2014.990411 25424047

[B79] El-Tedawy D. M. Abd-Alhaseeb M. M. Helmy M. W. Ghoneim A. I. (2020). Systemic bee venom exerts anti-arthritic and anti-inflammatory properties in a rat model of arthritis. Biomed. Rep. 13 (4), 20–21. 10.3892/br.2020.1327 32765859 PMC7403832

[B80] Erkoc P. Von Reumont B. M. Lüddecke T. Henke M. Ulshöfer T. Vilcinskas A. (2022). The pharmacological potential of novel melittin variants from the honeybee and solitary bees against inflammation and cancer. Toxins 14 (12), 818. 10.3390/toxins14120818 36548715 PMC9786170

[B81] Er-Rouassi H. Bakour M. Touzani S. Vilas-Boas M. Falcão S. Vidal C. (2023). Beneficial effect of bee venom and its major components on facial nerve injury induced in mice. Biomolecules 13 (4), 680. 10.3390/biom13040680 37189427 PMC10135545

[B82] Essam Elenany Y. (2024). Factors affecting the quantity and quality of honey bee venom collected from entrance and inside the beehive under Egyptian conditions. J. Apic. Res., 1–7. 10.1080/00218839.2024.2328473

[B83] Fan X.-G. Pei S.-Y. Zhou D. Zhou P.-C. Huang Y. Hu X.-W. (2021). Melittin ameliorates inflammation in mouse acute liver failure via inhibition of PKM2-mediated Warburg effect. Acta Pharmacol. Sin. 42 (8), 1256–1266. 10.1038/s41401-020-00516-0 32939034 PMC8285470

[B84] Farook U. B. Dar S. A. Arifie U. Javid R. Khaliq N. Singh R. (2024). Bee venom: composition and therapeutic potential. Honey Bees, Beekeep. Bee Prod., 189–202.

[B85] Feng N. Feng Y. Tan J. Zhou C. Xu J. Chen Y. (2023). Inhibition of advance glycation end products formation, gastrointestinal digestion absorption and toxicity: a comprehensive review. Int. J. Biol. Macromol., 125814. 10.1016/j.ijbiomac.2023.125814 37451379

[B86] Frangieh J. Salma Y. Haddad K. Mattei C. Legros C. Fajloun Z. (2019). First characterization of the venom from apis mellifera syriaca, a honeybee from the middle east region. Toxins 11 (4), 191. 10.3390/toxins11040191 30935025 PMC6521255

[B87] Gajski G. Čimbora-Zovko T. Rak S. Osmak M. Garaj-Vrhovac V. (2016). Antitumour action on human glioblastoma A1235 cells through cooperation of bee venom and cisplatin. Cytotechnology 68, 1197–1205. 10.1007/s10616-015-9879-4 25916941 PMC4960167

[B88] Gajski G. Leonova E. Sjakste N. (2024). Bee venom: composition and anticancer properties. Toxins 16 (3), 117. 10.3390/toxins16030117 38535786 PMC10975291

[B89] Galante P. Campos G. a. A. Moser J. C. G. Martins D. B. Dos Santos Cabrera M. P. Rangel M. (2023). Exploring the therapeutic potential of an antinociceptive and anti-inflammatory peptide from wasp venom. Sci. Rep. 13 (1), 12491. 10.1038/s41598-023-38828-w 37528129 PMC10393941

[B90] Gao C. Jiang J. Tan Y. Chen S. (2023). Microglia in neurodegenerative diseases: mechanism and potential therapeutic targets. Signal Transduct. Target. Ther. 8 (1), 359. 10.1038/s41392-023-01588-0 37735487 PMC10514343

[B91] Gasanoff E. Liu Y. Li F. Hanlon P. Garab G. (2021). Bee venom melittin disintegrates the respiration of mitochondria in healthy cells and lymphoblasts, and induces the formation of non-bilayer structures in model inner mitochondrial membranes. Int. J. Mol. Sci. 22 (20), 11122. 10.3390/ijms222011122 34681781 PMC8538590

[B92] Ghadiri N. Javidan M. Sheikhi S. Taştan Ö. Parodi A. Liao Z. (2024). Bioactive peptides: an alternative therapeutic approach for cancer management. Front. Immunol. 15, 1310443. 10.3389/fimmu.2024.1310443 38327525 PMC10847386

[B93] Ghasemi N. Razavi S. Nikzad E. (2017). Multiple sclerosis: pathogenesis, symptoms, diagnoses and cell-based therapy. Cell. J. (Yakhteh) 19 (1), 1–10. 10.22074/cellj.2016.4867 PMC5241505 28367411

[B94] Giannenas I. Sidiropoulou E. Bonos E. Christaki E. Florou-Paneri P. (2020). Feed additives. Elsevier, 1–18.

[B95] Giribaldi J. Smith J. J. Schroeder C. I. (2021). Recent developments in animal venom peptide nanotherapeutics with improved selectivity for cancer cells. Biotechnol. Adv. 50, 107769. 10.1016/j.biotechadv.2021.107769 33989705

[B96] Gmachl M. Kreil G. (1993). Bee venom hyaluronidase is homologous to a membrane protein of mammalian sperm. Proc. Natl. Acad. Sci. 90 (8), 3569–3573. 10.1073/pnas.90.8.3569 7682712 PMC46342

[B97] Golubnitschaja O. Debald M. Yeghiazaryan K. Kuhn W. Pešta M. Costigliola V. (2016). Breast cancer epidemic in the early twenty-first century: evaluation of risk factors, cumulative questionnaires and recommendations for preventive measures. Tumor Biol. 37, 12941–12957. 10.1007/s13277-016-5168-x 27448308

[B98] Gong H. Hu X. Zhang L. Fa K. Liao M. Liu H. (2023). How do antimicrobial peptides disrupt the lipopolysaccharide membrane leaflet of Gram-negative bacteria? J. Colloid Interface Sci. 637, 182–192. 10.1016/j.jcis.2023.01.051 36701864

[B99] Grawish M. E. Mourad M. I. Esmaeil D. a. M. Ahmed R. A. Ateia I. M. Hany E. (2020). Emerging therapeutic modality enhancing the efficiency of chemotherapeutic agents against head and neck squamous cell carcinoma cell lines. Cancer Treat. Res. Commun. 25, 100242. 10.1016/j.ctarc.2020.100242 33249209

[B100] Grunwald T. Bockisch B. Spillner E. Ring J. Bredehorst R. Ollert M. W. (2006). Molecular cloning and expression in insect cells of honeybee venom allergen acid phosphatase (Api m 3). J. allergy Clin. Immunol. 117 (4), 848–854. 10.1016/j.jaci.2005.12.1331 16630944

[B101] Gu H. An H.-J. Kim J.-Y. Kim W.-H. Gwon M.-G. Kim H.-J. (2019). Bee venom attenuates Porphyromonas gingivalis and RANKL-induced bone resorption with osteoclastogenic differentiation. Food Chem. Toxicol. 129, 344–353. 10.1016/j.fct.2019.05.001 31055000

[B102] Gu H. Han S. M. Park K.-K. (2020). Therapeutic effects of apamin as a bee venom component for non-neoplastic disease. Toxins 12 (3), 195. 10.3390/toxins12030195 32204567 PMC7150898

[B103] Guo Q. Wang Y. Xu D. Nossent J. Pavlos N. J. Xu J. (2018). Rheumatoid arthritis: pathological mechanisms and modern pharmacologic therapies. Bone Res. 6 (1), 15. 10.1038/s41413-018-0016-9 29736302 PMC5920070

[B104] Gwon M.-G. An H.-J. Gu H. Kim Y.-A. Han S. M. Park K.-K. (2021). Apamin inhibits renal fibrosis via suppressing TGF-β1 and STAT3 signaling *in vivo* and *in vitro* . J. Mol. Med. 99 (9), 1265–1277. 10.1007/s00109-021-02087-x 34031696

[B105] Han S. Lee K. Yeo J. Kim W. Park K. (2011). Biological effects of treatment of an animal skin wound with honeybee (*Apis mellifera*. L) venom. J. Plastic, Reconstr. Aesthetic Surg. 64 (3), e67–e72. 10.1016/j.bjps.2010.08.022 20943448

[B106] Han S. M. Hong I. P. Woo S. O. Chun S. N. Park K. K. Nicholls Y. M. (2015). The beneficial effects of honeybee-venom serum on facial wrinkles in humans. Clin. interventions aging 10, 1587–1592. 10.2147/CIA.S84940 PMC4598227 26491274

[B107] Han S. M. Hong I. P. Woo S. O. Kim S. G. Jang H. R. Park K. K. (2017). Evaluation of the skin phototoxicity and photosensitivity of honeybee venom. J. Cosmet. Dermatology 16 (4), e68–e75. 10.1111/jocd.12350 28509378

[B108] Han S. M. Lee K. G. Pak S. C. (2013). Effects of cosmetics containing purified honeybee (*Apis mellifera* L.) venom on acne vulgaris. J. Integr. Med. 11 (5), 320–326. 10.3736/jintegrmed2013043 24063779

[B109] Hanafi M. Y. Zaher E. L. M. El-Adely S. E. M. Sakr A. Ghobashi A. H. M. Hemly M. H. (2018). The therapeutic effects of bee venom on some metabolic and antioxidant parameters associated with HFD-induced non-alcoholic fatty liver in rats. Exp. Ther. Med. 15 (6), 5091–5099. 10.3892/etm.2018.6028 29805535 PMC5952098

[B110] Haque S. Hussain A. Joshi H. Sharma U. Sharma B. Aggarwal D. (2023). Melittin: a possible regulator of cancer proliferation in preclinical cell culture and animal models. J. Cancer Res. Clin. Oncol. 149 (19), 17709–17726. 10.1007/s00432-023-05458-8 37919474 PMC11797004

[B111] Haque T. T. Weissler K. A. Schmiechen Z. Laky K. Schwartz D. M. Li J. (2024). TGFβ prevents IgE-mediated allergic disease by restraining T follicular helper 2 differentiation. Sci. Immunol. 9 (91), eadg8691. 10.1126/sciimmunol.adg8691 38241399

[B112] Hartmann A. Müllner J. Meier N. Hesekamp H. Van Meerbeeck P. Habert M.-O. (2016). Bee venom for the treatment of Parkinson disease–a randomized controlled clinical trial. PLoS One 11 (7), e0158235. 10.1371/journal.pone.0158235 27403743 PMC4942057

[B113] Hasan N. Hasani N. a. H. Omar E. Sham F. R. Fuad S. B. S. A. Karim M. K. A. (2023). A single targeted gamma-ray irradiation induced an acute modulation of immune cells and related cytokines in EMT6 mouse-bearing tumour model. Cancer Biomarkers 38 (1), 61–75. 10.3233/CBM-220268 37522193 PMC12412841

[B114] Hassani S. Esmaeili A. (2024). The neuroprotective effects of Ferulic acid in toxin-induced models of Parkinson's disease: a review. Ageing Res. Rev. 97, 102299. 10.1016/j.arr.2024.102299 38604452

[B115] Hegazi A. G. Abd Raboh F. A. Ramzy N. E. Shaaban D. M. Khader D. Y. (2013). Bee venom and propolis as new treatment modality in patients with localized plaque psoriases. Int. Res. J. Med. Med. Sci. 1 (1), 27–33.

[B116] Heron M. P. (2021). Deaths: leading causes for 2018. 34029179

[B117] Hossen M. S. Gan S. H. Khalil M. I. (2017a). Melittin, a potential natural toxin of crude bee venom: probable future arsenal in the treatment of diabetes mellitus. J. Chem. 2017, 1–7. 10.1155/2017/4035626

[B118] Hossen M. S. Gan S. H. Khalil M. I. J. J. O. C. (2017b). Melittin, a potential natural toxin of crude bee venom: probable future arsenal in the treatment of diabetes mellitus.

[B119] Hossen M. S. Shapla U. M. Gan S. H. Khalil M. I. (2016). Impact of bee venom enzymes on diseases and immune responses. Molecules 22 (1), 25. 10.3390/molecules22010025 28035985 PMC6155781

[B120] How K. Y. Song K. P. Chan K. G. (2016). Porphyromonas gingivalis: an overview of periodontopathic pathogen below the gum line. Front. Microbiol. 7, 53. 10.3389/fmicb.2016.00053 26903954 PMC4746253

[B121] Hozzein W. N. Badr G. Badr B. M. Allam A. Al Ghamdi A. Al-Wadaan M. A. (2018). Bee venom improves diabetic wound healing by protecting functional macrophages from apoptosis and enhancing Nrf2, Ang-1 and Tie-2 signaling. Mol. Immunol. 103, 322–335. 10.1016/j.molimm.2018.10.016 30366166

[B122] Huang J.-Y. Peng S.-F. Chueh F.-S. Chen P.-Y. Huang Y.-P. Huang W.-W. (2021). Melittin suppresses epithelial–mesenchymal transition and metastasis in human gastric cancer AGS cells via regulating Wnt/BMP associated pathway. Biosci. Biotechnol. Biochem. 85 (11), 2250–2262. 10.1093/bbb/zbab153 34482401

[B123] Hwang Y.-N. Kwon I.-S. Na H.-H. Park J.-S. Kim K.-C. (2022). Dual cytotoxic responses induced by treatment of A549 human lung cancer cells with sweet bee venom in a dose-dependent manner. J. Pharmacopuncture 25 (4), 390–395. 10.3831/KPI.2022.25.4.390 36628342 PMC9806155

[B124] Isidorov V. Zalewski A. Zambrowski G. Swiecicka I. (2023). Chemical composition and antimicrobial properties of honey bee venom. Molecules 28 (10), 4135. 10.3390/molecules28104135 37241876 PMC10223701

[B125] Issam A. L. A. Zimmermann S. Reichling J. Wink M. (2015). Pharmacological synergism of bee venom and melittin with antibiotics and plant secondary metabolites against multi-drug resistant microbial pathogens. Phytomedicine 22 (2), 245–255. 10.1016/j.phymed.2014.11.019 25765829

[B126] Ivanov E. Akhmetshina M. Erdiakov A. Gavrilova S. (2023). Sympathetic system in wound healing: multistage control in normal and diabetic skin. Int. J. Mol. Sci. 24 (3), 2045. 10.3390/ijms24032045 36768369 PMC9916402

[B127] Izhar M. P. Hafeez A. Kushwaha P. Simrah (2023). Drug delivery through niosomes: a comprehensive review with therapeutic applications. J. Clust. Sci. 34 (5), 2257–2273. 10.1007/s10876-023-02423-w

[B128] Jafari Z. Sadeghi S. Dehaghi M. M. Bigham A. Honarmand S. Tavasoli A. (2024). Immunomodulatory activities and biomedical applications of melittin and its recent advances. Arch. Pharm. 357, e2300569. 10.1002/ardp.202300569 38251938

[B129] Jafarzadeh A. Sheikhi A. Jafarzadeh Z. Nemati M. (2023). Differential roles of regulatory T cells in Alzheimer's disease. Cell. Immunol. 393, 104778. 10.1016/j.cellimm.2023.104778 37907046

[B130] Jamasbi E. Mularski A. Separovic F. (2016). Model membrane and cell studies of antimicrobial activity of melittin analogues. Curr. Top. Med. Chem. 16 (1), 40–45. 10.2174/1568026615666150703115919 26139117

[B131] Jang S. Kim K. H. (2020). Clinical effectiveness and adverse events of bee venom therapy: a systematic review of randomized controlled trials. Toxins 12 (9), 558. 10.3390/toxins12090558 32872552 PMC7551670

[B132] Jeon S. Kim N.-H. Koo B.-S. Lee H.-J. Lee A.-Y. (2007). Bee venom stimulates human melanocyte proliferation, melanogenesis, dendricity and migration. Exp. Mol. Med. 39 (5), 603–613. 10.1038/emm.2007.66 18059136

[B133] Jeong C. H. Cheng W. N. Bae H. Lee K. W. Han S. M. Petriello M. C. (2017). Bee venom decreases LPS-induced inflammatory responses in bovine mammary epithelial cells. J. Microbiol. Biotechnol. 27 (10), 1827–1836. 10.4014/jmb.1706.06003 28813781

[B134] Jia L. Han N. Du J. Guo L. Luo Z. Liu Y. (2019). Pathogenesis of important virulence factors of Porphyromonas gingivalis via toll-like receptors. Front. Cell. Infect. Microbiol. 9, 262. 10.3389/fcimb.2019.00262 31380305 PMC6657652

[B135] Jung G. B. Huh J.-E. Lee H.-J. Kim D. Lee G.-J. Park H.-K. (2018). Anti-cancer effect of bee venom on human MDA-MB-231 breast cancer cells using Raman spectroscopy. Biomed. Opt. express 9 (11), 5703–5718. 10.1364/BOE.9.005703 30460157 PMC6238932

[B136] Kallarackal A. J. Simard J. M. Bailey A. M. (2013). The effect of apamin, a small conductance calcium activated potassium (SK) channel blocker, on a mouse model of neurofibromatosis 1. Behav. Brain Res. 237, 71–75. 10.1016/j.bbr.2012.09.009 22983217

[B137] Kamran M. R. Zargan J. Alikhani H. K. Hajinoormohamadi A. (2020). The Comparative cytotoxic effects of apis mellifera crude venom on MCF-7 Breast Cancer cell line in 2D and 3D cell cultures. Int. J. peptide Res. Ther. 26, 1819–1828. 10.1007/s10989-019-09979-0

[B138] Khalil A. Elesawy B. H. Ali T. M. Ahmed O. M. (2021). Bee venom: from venom to drug. Molecules 26 (16), 4941. 10.3390/molecules26164941 34443529 PMC8400317

[B139] Khaliq R. Iqbal P. Wani A. Y. (2022). Handbook of research on natural products and their bioactive compounds as cancer therapeutics. IGI Glob., 119–152.

[B140] Khan N. Niazi Z. R. Akhtar A. Khan M. M. Khan S. Baloch N. (2018). Hyaluronidases: a therapeutic enzyme. Protein Peptide Lett. 25 (7), 663–676. 10.2174/0929866525666180629121823 29956608

[B141] Kharroubi A. T. Darwish H. M. (2015). Diabetes mellitus: the epidemic of the century. World J. diabetes 6 (6), 850–867. 10.4239/wjd.v6.i6.850 26131326 PMC4478580

[B142] Khateeb S. Taha E. F. S. (2024). Comparative study of the anti-inflammatory activity of etoricoxib and Matcha green tea against acute kidney injury induced by gamma radiation in rats. Int. J. Radiat. Biol. 100, 940–964. 10.1080/09553002.2024.2338515 38647648

[B143] Khulan T. S. Ambaga M. Chimedragcha C. H. (2015). Effect of honey bee venom (*Apis mellifera*) on hyperglycemia and hyperlipidemia in alloxan induced diabetic rabbits. J. Diabetes Metab. 6, 507. 10.4172/2155-6156.1000507

[B144] Kim B. Y. Jin B. R. (2016). Molecular characterization of a venom acid phosphatase from the Asiatic honeybee *Apis cerana* . J. Asia-Pacific Entomology 19 (3), 793–797. 10.1016/j.aspen.2016.07.013

[B145] Kim D.-H. Han S.-M. Choi Y.-S. Kang H.-K. Lee H.-G. Lee K.-W. (2019a). Effects of dietary bee venom on serum characteristic, antioxidant activity and liver fatty acid composition in broiler chickens. Korean J. Poult. Sci. 46 (1), 39–46. 10.5536/kjps.2019.46.1.39

[B146] Kim D.-H. Han S.-M. Keum M. C. Lee S. An B.-K. Lee S. R. (2018). Evaluation of bee venom as a novel feed additive in fast-growing broilers. Br. Poult. Sci. 59 (4), 435–442. 10.1080/00071668.2018.1476675 29774758

[B147] Kim D.-H. Lee H.-W. Park H.-W. Lee H.-W. Chun K.-H. (2020a). Bee venom inhibits the proliferation and migration of cervical-cancer cells in an HPV E6/E7-dependent manner. BMB Rep. 53 (8), 419–424. 10.5483/BMBRep.2020.53.8.031 32317085 PMC7473477

[B148] Kim H. Hong J. Y. Lee J. Jeon W.-J. Ha I.-H. (2021a). Apamin enhances neurite outgrowth and regeneration after laceration injury in cortical neurons. Toxins 13 (9), 603. 10.3390/toxins13090603 34564607 PMC8472698

[B149] Kim H. Keum D. J. Kwak J. W. Chung H.-S. Bae H. (2014). Bee venom phospholipase A2 protects against acetaminophen-induced acute liver injury by modulating regulatory T cells and IL-10 in mice. PLoS One 9 (12), e114726. 10.1371/journal.pone.0114726 25478691 PMC4257707

[B150] Kim H. Park S.-Y. Lee G. (2019b). Potential therapeutic applications of bee venom on skin disease and its mechanisms: a literature review. Toxins 11 (7), 374. 10.3390/toxins11070374 31252651 PMC6669657

[B151] Kim J.-Y. An H.-J. Kim W.-H. Park Y.-Y. Park K. D. Park K.-K. (2017). Apamin suppresses biliary fibrosis and activation of hepatic stellate cells. Int. J. Mol. Med. 39 (5), 1188–1194. 10.3892/ijmm.2017.2922 28405682 PMC5403474

[B152] Kim J.-Y. Jang H.-J. Leem J. Kim G.-M. (2021b). Protective effects of bee venom-derived phospholipase A2 against cholestatic liver disease in mice. Biomedicines 9 (8), 992. 10.3390/biomedicines9080992 34440196 PMC8394029

[B153] Kim J.-Y. Kim K.-H. Lee W.-R. An H.-J. Lee S.-J. Han S.-M. (2015a). Apamin inhibits PDGF-BB-induced vascular smooth muscle cell proliferation and migration through suppressions of activated Akt and Erk signaling pathway. Vasc. Pharmacol. 70, 8–14. 10.1016/j.vph.2014.12.004 25737404

[B154] Kim J.-Y. Lee W.-R. Kim K.-H. An H.-J. Chang Y.-C. Han S.-M. (2015b). Effects of bee venom against Propionibacterium acnes-induced inflammation in human keratinocytes and monocytes. Int. J. Mol. Med. 35 (6), 1651–1656. 10.3892/ijmm.2015.2180 25872535

[B155] Kim J.-Y. Leem J. Park K.-K. (2020b). Antioxidative, antiapoptotic, and anti-inflammatory effects of apamin in a murine model of lipopolysaccharide-induced acute kidney injury. Molecules 25 (23), 5717. 10.3390/molecules25235717 33287398 PMC7731169

[B156] Kim K. H. Lee S. Y. Shin J. Hwang J.-T. Jeon H. N. Bae H. (2019c). Dose-dependent neuroprotective effect of standardized bee venom phospholipase A2 against MPTP-induced Parkinson’s disease in mice. Front. Aging Neurosci. 11, 80. 10.3389/fnagi.2019.00080 31024294 PMC6462482

[B157] Kim S.-J. Park J.-H. Kim K.-H. Lee W.-R. An H.-J. Min B.-K. (2012). Apamin inhibits THP-1-derived macrophage apoptosis via mitochondria-related apoptotic pathway. Exp. Mol. pathology 93 (1), 129–134. 10.1016/j.yexmp.2012.04.003 22537544

[B158] Kim W. K. W. Kim Minjoon K. M. Go Donghyun G. D. Min Byungil M. B. Na Heungsik N. H. Kim Sunkwang K. S. (2016). Combined effects of bee venom acupuncture and morphine on oxaliplatin-induced neuropathic pain in mice. 10.3390/toxins8020033 PMC4773786 26805884

[B159] Kim Y.-W. Chaturvedi P. K. Chun S. N. Lee Y. G. Ahn W. S. (2015c). Honeybee venom possesses anticancer and antiviral effects by differential inhibition of HPV E6 and E7 expression on cervical cancer cell line. Oncol. Rep. 33 (4), 1675–1682. 10.3892/or.2015.3760 25633640

[B160] Kim Jungyeon K. J. Kim Kyunghyun K. K. Lee Wooram L. W. An Hyunjin A. H. Lee S. L. S. Han S. H. S. (2015). Apamin inhibits PDGF-BB-induced vascular smooth muscle cell proliferation and migration through suppressions of activated Akt and Erk signaling pathway. 10.1016/j.vph.2014.12.004 25737404

[B161] Koburova K. L. Michailova S. G. Shkenderov S. V. (1985). Further investigation on the antiinflammatory properties of adolapin--bee venom polypeptide. Acta physiologica Pharmacol. Bulg. 11 (2), 50–55. 2996298

[B162] Kurek-Górecka A. Komosinska-Vassev K. Rzepecka-Stojko A. Olczyk P. (2020). Bee venom in wound healing. Molecules 26 (1), 148. 10.3390/molecules26010148 33396220 PMC7795515

[B163] Kuzmenkov A. I. Peigneur S. Nasburg J. A. Mineev K. S. Nikolaev M. V. Pinheiro-Junior E. L. (2022). Apamin structure and pharmacology revisited. Front. Pharmacol. 13, 977440. 10.3389/fphar.2022.977440 36188602 PMC9523135

[B164] Kwon Y.-B. Kang M.-S. Han H.-J. Beitz A. J. Lee J.-H. (2001). Visceral antinociception produced by bee venom stimulation of the Zhongwan acupuncture point in mice: role of alpha(2) adrenoceptors. Neurosci. Lett. 308 (2), 133–137. 10.1016/s0304-3940(01)01989-9 11457577

[B165] Lamptey R. N. L. Chaulagain B. Trivedi R. Gothwal A. Layek B. Singh J. (2022). A review of the common neurodegenerative disorders: current therapeutic approaches and the potential role of nanotherapeutics. Int. J. Mol. Sci. 23 (3), 1851. 10.3390/ijms23031851 35163773 PMC8837071

[B166] Lebel A. A. Kisembo M. V. Soucy M.-F. N. Hébert M. P. A. Boudreau L. H. (2021). Molecular characterization of the anticancer properties associated with bee venom and its components in glioblastoma multiforme. Chemico-Biological Interact. 347, 109622. 10.1016/j.cbi.2021.109622 34375656

[B167] Lee C. Bae S.-J. S. Joo H. Bae H. (2017). Melittin suppresses tumor progression by regulating tumor-associated macrophages in a Lewis lung carcinoma mouse model. Oncotarget 8 (33), 54951–54965. 10.18632/oncotarget.18627 28903394 PMC5589633

[B168] Lee C. H. Yoon J.-Y. Shim S.-E. Kim J. H. Kim J.-Y. Kim H.-N. (2019a). A retrospective study on the clinical safety of bee venom pharmacopuncture at craniofacial acupuncture points for the treatment of facial disorders.

[B169] Lee G. Bae H. (2016a). Anti-inflammatory applications of melittin, a major component of bee venom: detailed mechanism of action and adverse effects. Molecules 21 (5), 616. 10.3390/molecules21050616 27187328 PMC6273919

[B170] Lee G. Bae H. (2016b). Bee venom phospholipase A2: yesterday’s enemy becomes today’s friend. Toxins 8 (2), 48. 10.3390/toxins8020048 26907347 PMC4773801

[B171] Lee G. Kang G.-H. Bae H. (2019b). Bee venom phospholipase A2 suppression of experimental autoimmune encephalomyelitis is dependent on its enzymatic activity. Mol. Cell. Toxicol. 15, 307–313. 10.1007/s13273-019-0034-8

[B172] Lee H.-S. Kim Y. S. Lee K.-S. Seo H.-S. Lee C.-Y. Kim K. K. (2021). Detoxification of bee venom increases its anti-inflammatory activity and decreases its cytotoxicity and allergenic activity. Appl. Biochem. Biotechnol. 193, 4068–4082. 10.1007/s12010-021-03653-2 34542820 PMC8450311

[B173] Lee J. A. Singletary E. Charlton N. (2020). Methods of honey bee stinger removal: a systematic review of the literature. Cureus 12 (5), e8078. 10.7759/cureus.8078 32542133 PMC7292703

[B174] Lee J.-D. Park H.-J. Chae Y. Lim S. (2005). An overview of bee venom acupuncture in the treatment of arthritis. Evidence-based complementary Altern. Med. 2, 79–84. 10.1093/ecam/neh070 PMC1062163 15841281

[B175] Lee J.-H. Li D. X. Yoon H. Go D. Quan F. S. Min B.-I. (2014a). Serotonergic mechanism of the relieving effect of bee venom acupuncture on oxaliplatin-induced neuropathic cold allodynia in rats. BMC complementary Altern. Med. 14, 471–477. 10.1186/1472-6882-14-471 PMC4295325 25481535

[B176] Lee K. S. Kim B. Y. Yoon H. J. Choi Y. S. Jin B. R. (2016a). Secapin, a bee venom peptide, exhibits anti-fibrinolytic, anti-elastolytic, and anti-microbial activities. Dev. Comp. Immunol. 63, 27–35. 10.1016/j.dci.2016.05.011 27208884

[B177] Lee K. S. Kim B. Y. Yoon H. J. Jin B. R. (2023). Proteases and protease inhibitors in bee venoms. J. Apic. 38 (4), 391–396. 10.17519/apiculture.2023.11.38.4.391

[B178] Lee M. J. Jang M. Choi J. Lee G. Min H. J. Chung W.-S. (2016b). Bee venom acupuncture alleviates experimental autoimmune encephalomyelitis by upregulating regulatory T cells and suppressing Th1 and Th17 responses. Mol. Neurobiol. 53, 1419–1445. 10.1007/s12035-014-9012-2 25579380

[B179] Lee S.-H. Choi S.-M. Yang E. J. (2014b). Melittin ameliorates the inflammation of organs in an amyotrophic lateral sclerosis animal model. Exp. Neurobiol. 23 (1), 86–92. 10.5607/en.2014.23.1.86 24737943 PMC3984960

[B180] Lee Jaedong L. J. Kim Suyoung K. S. Kim Taewoo K. T. Lee Sanghoon L. S. Yang Hyungin Y. H. Lee Dooik L. D. (2004). Anti-inflammatory effect of bee venom on type II collagen-induced arthritis.

[B181] Li B. Huang Y. Zou Q. (2023). Peptide‐based nanoarchitectonics for the treatment of liver fibrosis. ChemBioChem 24 (9), e202300002. 10.1002/cbic.202300002 36781383

[B182] Li J. Ke T. He C. Cao W. Wei M. Zhang L. (2010). The anti-arthritic effects of synthetic melittin on the complete Freund's adjuvant-induced rheumatoid arthritis model in rats. Am. J. Chin. Med. 38 (06), 1039–1049. 10.1142/S0192415X10008457 21061459

[B183] Li X. Zhu S. Li Z. Meng Y.-Q. Huang S.-J. Yu Q.-Y. (2022). Melittin induces ferroptosis and ER stress-CHOP-mediated apoptosis in A549 cells. Free Radic. Res. 56 (5-6), 398–410. 10.1080/10715762.2022.2131551 36194238

[B184] Lim H. N. Baek S. B. Jung H. J. (2019). Bee venom and its peptide component melittin suppress growth and migration of melanoma cells via inhibition of PI3K/AKT/mTOR and MAPK pathways. Molecules 24 (5), 929. 10.3390/molecules24050929 30866426 PMC6429308

[B185] Lin T.-Y. Hsieh C.-L. (2020). Clinical applications of bee venom acupoint injection. Toxins 12 (10), 618. 10.3390/toxins12100618 32992601 PMC7601520

[B186] Liu F. Chen F. Yang L. Qiu F. Zhong G. Gao S. (2023a). Melittin acupoint injection in attenuating bone erosion in collagen-induced arthritis mice via inhibition of the RANKL/NF-κB signaling pathway. Quantitative Imaging Med. Surg. 13 (9), 5996–6013. 10.21037/qims-23-254 PMC10498218 37711782

[B187] Liu H. Bai Y. Huang C. Wang Y. Ji Y. Du Y. (2023b). Recent progress of electrospun herbal medicine nanofibers. Biomolecules 13 (1), 184. 10.3390/biom13010184 36671570 PMC9855805

[B188] López-Incera A. Nouvian M. Ried K. Müller T. Briegel H. J. (2021). Honeybee communication during collective defence is shaped by predation. BMC Biol. 19, 106–116. 10.1186/s12915-021-01028-x 34030690 PMC8147350

[B189] Lv C. Chen J. Huang F. Fang F. Li B. (2023). Melittin inhibits the proliferation migration and invasion of HCC cells by regulating ADAMTS9-AS2 demethylation. Toxicon 222, 106996. 10.1016/j.toxicon.2022.106996 36535531

[B190] Małek A. Kocot J. Mitrowska K. Posyniak A. Kurzepa J. (2022). Bee venom effect on glioblastoma cells viability and gelatinase secretion. Front. Neurosci. 16, 792970. 10.3389/fnins.2022.792970 35221898 PMC8873382

[B191] Małek A. Strzemski M. Kurzepa J. Kurzepa J. (2023). Can bee venom be used as anticancer agent in modern medicine? Cancers 15 (14), 3714. 10.3390/cancers15143714 37509375 PMC10378503

[B192] Mansour G. H. El-Magd M. A. Mahfouz D. H. Abdelhamid I. A. Mohamed M. F. Ibrahim N. S. (2021). Bee venom and its active component Melittin synergistically potentiate the anticancer effect of Sorafenib against HepG2 cells. Bioorg. Chem. 116, 105329. 10.1016/j.bioorg.2021.105329 34544028

[B193] Martinello M. Mutinelli F. (2021). Antioxidant activity in bee products: a review. Antioxidants 10 (1), 71. 10.3390/antiox10010071 33430511 PMC7827872

[B194] Meligi N. M. Ismail S. A. Tawfik N. S. (2020). Protective effects of honey and bee venom against lipopolysaccharide and carbon tetrachloride-induced hepatoxicity and lipid peroxidation in rats. Toxicol. Res. 9 (5), 693–705. 10.1093/toxres/tfaa077 PMC7640919 33178430

[B195] Memariani H. Memariani M. (2021). Melittin as a promising anti-protozoan peptide: current knowledge and future prospects. Amb. Express 11 (1), 69. 10.1186/s13568-021-01229-1 33983454 PMC8119515

[B196] Memariani H. Memariani M. Moravvej H. Shahidi-Dadras M. (2020). Melittin: a venom-derived peptide with promising anti-viral properties. Eur. J. Clin. Microbiol. Infect. Dis. 39 (1), 5–17. 10.1007/s10096-019-03674-0 31422545 PMC7224078

[B197] Memariani H. Memariani M. Shahidi-Dadras M. Nasiri S. Akhavan M. M. Moravvej H. (2019). Melittin: from honeybees to superbugs. Appl. Microbiol. Biotechnol. 103, 3265–3276. 10.1007/s00253-019-09698-y 30824944

[B198] Mi W. Meng M. Xu F. Sun L. (2024). Efficacy of acupuncture as adjunct therapy for sleep disorders in Parkinson's disease: a systematic review and meta-analysis. Complementary Ther. Med. 82, 103044. 10.1016/j.ctim.2024.103044 38679147

[B199] Mohammadi-Rad M. Ghasemi N. Aliomrani M. (2019). Evaluation of apamin effects on myelination process in C57BL/6 mice model of multiple sclerosis. Res. Pharm. Sci. 14 (5), 424–431. 10.4103/1735-5362.268203 31798659 PMC6827192

[B200] Mohanty M. C. Murhekar M. M. (2023). Anti-viral metabolites from medicinal plants. Springer, 429–460.

[B201] Montoro A. Obrador E. Mistry D. Forte G. I. Bravatà V. Minafra L. (2023). Radiobiology textbook. Springer, 571–628.

[B202] Moon D.-O. Park S.-Y. Lee K.-J. Heo M.-S. Kim K.-C. Kim M.-O. (2007). Bee venom and melittin reduce proinflammatory mediators in lipopolysaccharide-stimulated BV2 microglia. Int. Immunopharmacol. 7 (8), 1092–1101. 10.1016/j.intimp.2007.04.005 17570326

[B203] Moreno M. Giralt E. (2015). Three valuable peptides from bee and wasp venoms for therapeutic and biotechnological use: melittin, apamin and mastoparan. Toxins 7 (4), 1126–1150. 10.3390/toxins7041126 25835385 PMC4417959

[B204] Morgan E. Arnold M. Gini A. Lorenzoni V. Cabasag C. J. Laversanne M. (2023). Global burden of colorectal cancer in 2020 and 2040: incidence and mortality estimates from GLOBOCAN. Gut 72 (2), 338–344. 10.1136/gutjnl-2022-327736 36604116

[B205] Morin Jr P. Boudreau L. H. (2024). Natural molecules in neuroprotection and neurotoxicity. Elsevier, 405–413.

[B206] Murakami M. Nakatani Y. Atsumi G.-I. Inoue K. Kudo I. (2017). Regulatory functions of phospholipase A 2. Crit. Reviews™ Immunol. 37 (2-6), 121–179. 10.1615/critrevimmunol.v37.i2-6.20 29773019

[B207] Nainu F. Masyita A. Bahar M. A. Raihan M. Prova S. R. Mitra S. (2021). Pharmaceutical prospects of bee products: special focus on anticancer, antibacterial, antiviral, and antiparasitic properties. Antibiotics 10 (7), 822. 10.3390/antibiotics10070822 34356743 PMC8300842

[B208] Nassan M. Videnovic A. (2022). Circadian rhythms in neurodegenerative disorders. Nat. Rev. Neurol. 18 (1), 7–24. 10.1038/s41582-021-00577-7 34759373

[B209] Nasser Atia G. A. Barai H. R. Shalaby H. K. Ali N. G. Morsy S. M. Ghobashy M. M. (2022). Baghdadite: a novel and promising calcium silicate in regenerative dentistry and medicine. ACS omega 7 (49), 44532–44541. 10.1021/acsomega.2c05596 36530225 PMC9753547

[B210] Nitecka-Buchta A. Buchta P. Tabeńska-Bosakowska E. Walczyńska-Dragoń K. Baron S. (2014). Myorelaxant effect of bee venom topical skin application in patients with RDC/TMD Ia and RDC/TMD Ib: a randomized, double blinded study. BioMed Res. Int. 2014, 296053. 10.1155/2014/296053 25050337 PMC4094729

[B211] Ojha S. Yadav S. Aggarwal B. Gupta S. K. Mishra S. (2023). Considering the conception of nanotechnology integrated on herbal formulation for the management of cancer. Lett. Drug Des. Discov. 20 (10), 1437–1457. 10.2174/1570180819666220901093732

[B212] Oliveira Orsi R. Zaluski R. De Barros L. C. Barraviera B. Pimenta D. C. Ferreira Junior R. S. (2024). Standardized guidelines for Africanized honeybee venom production needed for development of new apilic antivenom. J. Toxicol. Environ. Health, Part B 27, 73–90. 10.1080/10937404.2023.2300786 38247328

[B213] Otręba M. Marek Ł. Tyczyńska N. Stojko J. Rzepecka-Stojko A. (2021). Bee venom, honey, and royal jelly in the treatment of bacterial infections of the oral cavity: a review. Life 11 (12), 1311. 10.3390/life11121311 34947842 PMC8709083

[B214] Özgenç Ö. Sevin S. (2023). Arı zehirinin yağ dokusu kaynaklı mezenkimal kök hücre üzerindeki yara iyileştirici etkileri. Veteriner Hekimler Derneği Derg. 94 (1), 59–66. 10.33188/vetheder.1183380

[B215] Palm N. W. Rosenstein R. K. Yu S. Schenten D. D. Florsheim E. Medzhitov R. (2013). Bee venom phospholipase A2 induces a primary type 2 response that is dependent on the receptor ST2 and confers protective immunity. Immunity 39 (5), 976–985. 10.1016/j.immuni.2013.10.006 24210353 PMC3852615

[B216] Pandey P. Khan F. Khan M. A. Kumar R. Upadhyay T. K. (2023a). An updated review summarizing the anticancer efficacy of melittin from bee venom in several models of human cancers. Nutrients 15 (14), 3111. 10.3390/nu15143111 37513529 PMC10385528

[B217] Pandey P. Khan F. Khan M. A. Kumar R. Upadhyay T. K. J. N. (2023b). An updated review summarizing the anticancer efficacy of melittin from bee venom in several models of human cancers. Nutrients 15 (14), 3111. 10.3390/nu15143111 37513529 PMC10385528

[B218] Park H. J. Lee S. H. Son D. J. Oh K. W. Kim K. H. Song H. S. (2004). Antiarthritic effect of bee venom: inhibition of inflammation mediator generation by suppression of NF-kappaB through interaction with the p50 subunit. Arthritis and rheumatism 50 (11), 3504–3515. 10.1002/art.20626 15529353

[B219] Park J. Kwon O. An H.-J. Park K. K. (2018). Antifungal effects of bee venom components on Trichophyton rubrum: a novel approach of bee venom study for possible emerging antifungal agent. Ann. dermatology 30 (2), 202–210. 10.5021/ad.2018.30.2.202 PMC5839892 29606818

[B220] Park J. E. Kim K. H. Kang S. Lee E. K. Kim J.-C. Jang B.-H. (2019). Usage status and satisfaction with pharmacopuncture in Korea: a survey among Korean medicine doctors. Eur. J. Integr. Med. 27, 121–130. 10.1016/j.eujim.2019.03.001

[B221] Park J.-H. Kum Y.-S. Lee T.-I. Kim S.-J. Lee W.-R. Kim B.-I. (2011). Melittin attenuates liver injury in thioacetamide-treated mice through modulating inflammation and fibrogenesis. Exp. Biol. Med. 236 (11), 1306–1313. 10.1258/ebm.2011.011127 21969711

[B222] Park S. Erdogan S. Hwang D. Hwang S. Han E. H. Lim Y.-H. (2016). Bee venom promotes hair growth in association with inhibiting 5α-reductase expression. Biol. Pharm. Bull. 39 (6), 1060–1068. 10.1248/bpb.b16-00158 27040904

[B223] Park Heegeun P. H. Lee Kwangsik L. K. Kim Boyeon K. B. Yoon Hyungjoo Y. H. Choi Y. C. Y. Lee Kyeongyong L. K. (2018). Honeybee (*Apis cerana*) vitellogenin acts as an antimicrobial and antioxidant agent in the body and venom. 10.1016/j.dci.2018.04.001 29621531

[B224] Pavel C. I. Mărghitaş L. A. Dezmirean D. S. Tomoş L. I. Bonta V. Şapcaliu A. (2014). Comparison between local and commercial royal jelly—use of antioxidant activity and 10-hydroxy-2-decenoic acid as quality parameter. J. Apic. Res. 53 (1), 116–123. 10.3896/ibra.1.53.1.12

[B225] Peng Y. Zong Y. Wang D. Chen J. Chen Z.-S. Peng F. (2023). Current drugs for HIV-1: from challenges to potential in HIV/AIDS. Front. Pharmacol. 14, 1294966. 10.3389/fphar.2023.1294966 37954841 PMC10637376

[B226] Peralta F. De La Iglesia F. V. Gomis A. (2023). OXALIPLATIN INDUCES COLD AND MECHANICAL ALLODYNIA IN A SEX-DEPENDENT MANNER AND ALTERS MECHANOSENSITIVITY OF MOUSE SOMATOSENSORY NEURONS. IBRO Neurosci. Rep. 15, S707. 10.1016/j.ibneur.2023.08.1432

[B227] Pereira A. F. M. Albano M. Alves F. C. B. Andrade B. F. M. T. Furlanetto A. Rall V. L. M. (2020). Influence of apitoxin and melittin from *Apis mellifera* bee on *Staphylococcus aureus* strains. Microb. Pathog. 141, 104011. 10.1016/j.micpath.2020.104011 32004624

[B228] Petronek M. S. Spitz D. R. Allen B. G. (2021). Iron–sulfur cluster biogenesis as a critical target in cancer. Antioxidants 10 (9), 1458. 10.3390/antiox10091458 34573089 PMC8465902

[B229] Piek T. (2013). Venoms of the Hymenoptera: biochemical, pharmacological and behavioural aspects. Elsevier.

[B230] Pinmanee P. Sompinit K. Jantimaporn A. Khongkow M. Haltrich D. Nimchua T. (2023). Purification and immobilization of superoxide dismutase obtained from *Saccharomyces cerevisiae* TBRC657 on bacterial cellulose and its protective effect against oxidative damage in fibroblasts. Biomolecules 13 (7), 1156. 10.3390/biom13071156 37509191 PMC10377281

[B231] Portelli P. (2020). Insects and other invertebrates in Maltese culture and tradition.

[B232] Proulx É. Power S. K. Oliver D. K. Sargin D. Mclaurin J. Lambe E. K. (2020). Apamin improves prefrontal nicotinic impairment in mouse model of Alzheimer’s disease. Cereb. Cortex 30 (2), 563–574. 10.1093/cercor/bhz107 31188425

[B233] Pucca M. B. Cerni F. A. Oliveira I. S. Jenkins T. P. Argemí L. Sørensen C. V. (2019). Bee updated: current knowledge on bee venom and bee envenoming therapy. Front. Immunol. 10, 2090. 10.3389/fimmu.2019.02090 31552038 PMC6743376

[B234] Qi X. Aiyasamy K. Alenezi S. K. Alanazi I. M. Alshammari M. S. Ibrahim I. a. A. (2023). Anti-nociceptive and anti-inflammatory activities of visnagin in different nociceptive and inflammatory mice models. Appl. Biochem. Biotechnol. 196, 3441–3455. 10.1007/s12010-023-04677-6 37659050

[B235] Quatorze M. Silva F. Duarte A. I. Cardoso J. Caetano C. Ramalho-Santos J. (2024). Amyotrophic lateral sclerosis: when nerve cells run out of power.

[B236] Rady I. Siddiqui I. A. Rady M. Mukhtar H. (2017). Melittin, a major peptide component of bee venom, and its conjugates in cancer therapy. Cancer Lett. 402, 16–31. 10.1016/j.canlet.2017.05.010 28536009 PMC5682937

[B237] Rasinar A.-D. Moraru D. Lazăr R. Pătruică S. (2023). The biotechnological potential of bee venom. Sci. Pap. ANIMAL Sci. Biotechnol. 56 (1), 104.

[B238] Regeai S. O. Abusrer S. A. Shibani N. S. (2021). Low semen quality and adverse histological changes in testes of adult male mice treated with bee venom (*Apis mellifera*). Open Veterinary J. 11 (1), 70–79. 10.4314/ovj.v11i1.11 PMC8057216 33898286

[B239] Reuter S. Raspe J. Taube C. (2024). Microbes little helpers and suppliers for therapeutic asthma approaches. Respir. Res. 25 (1), 29. 10.1186/s12931-023-02660-7 38218816 PMC10787474

[B240] Rocha M. M. Dariva I. Zornoff G. C. De Laurentis G. S. Mendes G. C. Santana M. G. (2022). A new therapeutic approach for bone metastasis in colorectal cancer: intratumoral melittin. J. Venom. Animals Toxins Incl. Trop. Dis. 28, e20210067. 10.1590/1678-9199-JVATITD-2021-0067 PMC8922758 35321289

[B241] Rosenblum M. D. Remedios K. A. Abbas A. K. (2015). Mechanisms of human autoimmunity. J. Clin. investigation 125 (6), 2228–2233. 10.1172/JCI78088 PMC4518692 25893595

[B242] Rouhi A. Yousefi Y. Falah F. Azghandi M. Behbahani B. A. Tabatabaei-Yazdi F. (2024). Exploring the potential of melittin peptide: expression, purification, anti-pathogenic properties, and promising applications as a bio-preservative for beef slices. LWT 199, 116083. 10.1016/j.lwt.2024.116083

[B243] Roversi D. Troiano C. Salnikov E. Giordano L. Riccitelli F. De Zotti M. (2023). Effects of antimicrobial peptides on membrane dynamics: a comparison of fluorescence and NMR experiments. Biophys. Chem. 300, 107060. 10.1016/j.bpc.2023.107060 37336097

[B244] Rzetecka N. Matuszewska E. Plewa S. Matysiak J. Klupczynska-Gabryszak A. (2024). Bee products as valuable nutritional ingredients: determination of broad free amino acid profiles in bee pollen, royal jelly, and propolis. J. Food Compos. Analysis 126, 105860. 10.1016/j.jfca.2023.105860

[B245] Sadek M. (2023). The sting that cures: bee venom and its therapeutic future (apis mellifera).

[B246] Salama M. A. Younis M. A. Talaat R. M. (2021). Cytokine and inflammatory mediators are associated with cytotoxic, anti-inflammatory and apoptotic activity of honeybee venom. J. Complementary Integr. Med. 18 (1), 75–86. 10.1515/jcim-2019-0182 32452823

[B247] Salem R. A. Marzouk W. M. (2024). Histological changes of bee worker (*Apis mellifera* L.) by bee venom and amino acids feeding. Kufa J. Agric. Sci. 16 (1), 177–188. 10.36077/kjas/2024/v16i1.13633

[B248] Salimo Z. M. Yakubu M. N. Da Silva E. L. De Almeida A. C. G. Chaves Y. O. Costa E. V. (2023). Chemistry and Pharmacology of Bergenin or its derivatives: a promising molecule. Biomolecules 13 (3), 403. 10.3390/biom13030403 36979338 PMC10046151

[B249] Sangboonruang S. Kitidee K. Chantawannakul P. Tragoolpua K. Tragoolpua Y. (2020). Melittin from Apis florea venom as a promising therapeutic agent for skin cancer treatment. Antibiotics 9 (8), 517. 10.3390/antibiotics9080517 32823904 PMC7460526

[B250] Saravanan D. Rafi S. M. Mohan M. (2023). Identification of novel Bioactivities from Bee venom to target TNF-α for cancer therapy. Archives Clin. Toxicol. 5 (1), 22–27. 10.46439/toxicology.5.021

[B251] Sarhan W. A. Azzazy H. M. E. (2017). Apitherapeutics and phage-loaded nanofibers as wound dressings with enhanced wound healing and antibacterial activity. Nanomedicine 12 (17), 2055–2067. 10.2217/nnm-2017-0151 28805554

[B252] Sawicka B. Messaoudi M. Achar R. R. Himathi M. U. Pszczółkowski P. (2024). Antidotes to toxins and drugs. Elsevier, 37–70.

[B253] Schmidt J. O. (2018). Clinical consequences of toxic envenomations by Hymenoptera. Toxicon 150, 96–104. 10.1016/j.toxicon.2018.05.013 29782951

[B254] Sevin S. Kivrak İ. Tutun H. Uyar R. Ayaz F. (2023). *Apis mellifera* anatoliaca venom exerted anti-inflammatory activity on LPS-stimulated mammalian macrophages by reducing the production of the inflammatory cytokines. Appl. Biochem. Biotechnol. 195 (5), 3194–3205. 10.1007/s12010-022-04284-x 36574137

[B255] Shaaban A. M. M. Hamza R. G. El shahat A. (2019). Studying the ameliorative effect of bee venom against damage and inflammation induced in gamma-irradiated rats. Arab J. Nucl. Sci. Appl. 52 (1), 178–184. 10.21608/ajnsa.2018.3107.1071

[B256] Shaik R. A. Alotaibi M. F. Nasrullah M. Z. Alrabia M. W. Asfour H. Z. Abdel-Naim A. B. (2023). Cordycepin-melittin nanoconjugate intensifies wound healing efficacy in diabetic rats. Saudi Pharm. J. 31 (5), 736–745. 10.1016/j.jsps.2023.03.014 37181143 PMC10172630

[B257] Sharaf M. Zahra A. A. Alharbi M. Mekky A. E. Shehata A. M. Alkhudhayri A. (2024a). Bee chitosan nanoparticles loaded with apitoxin as a novel approach to eradication of common human bacterial, fungal pathogens and treating cancer. Front. Microbiol. 15, 1345478. 10.3389/fmicb.2024.1345478 38559346 PMC10978808

[B258] Sharaf M. Zahra A. A. Alharbi M. Mekky A. E. Shehata A. M. Alkhudhayri A. (2024b). Bee chitosan nanoparticles loaded with apitoxin as a novel approach to eradication of common human bacterial, fungal pathogens and treating cancer. Front. Microbiol. 15, 1345478. 10.3389/fmicb.2024.1345478 38559346 PMC10978808

[B259] Sharif K. Sharif A. Jumah F. Oskouian R. Tubbs R. S. (2018). Rheumatoid arthritis in review: clinical, anatomical, cellular and molecular points of view. Clin. Anat. 31 (2), 216–223. 10.1002/ca.22980 28833647

[B260] Sharma A. Dheer D. Puri V. Alsayari A. Wahab S. Kesharwani P. (2024a). Insights of biopolymeric blended formulations for diabetic wound healing. Int. J. Pharm. 656, 124099. 10.1016/j.ijpharm.2024.124099 38614431

[B261] Sharma P. Vaiwala R. Gopinath A. K. Chockalingam R. Ayappa K. G. (2024b). Structure of the bacterial cell envelope and interactions with antimicrobials: insights from molecular dynamics simulations. Langmuir 40 (15), 7791–7811. 10.1021/acs.langmuir.3c03474 38451026

[B262] Shen L. Lee J. H. Joo J. C. Park S. J. Song Y. S. (2020). Bee venom acupuncture for shoulder pain: a systematic review and meta-analysis of randomized controlled trials. J. Pharmacopuncture 23 (2), 44–53. 10.3831/KPI.2020.23.008 32685232 PMC7338706

[B263] Shi P. Xie S. Yang J. Zhang Y. Han S. Su S. (2022). Pharmacological effects and mechanisms of bee venom and its main components: recent progress and perspective. Front. Pharmacol. 13, 1001553. 10.3389/fphar.2022.1001553 36238572 PMC9553197

[B264] Shi W. Li C. Li M. Zong X. Han D. Chen Y. (2016). Antimicrobial peptide melittin against Xanthomonas oryzae pv. oryzae, the bacterial leaf blight pathogen in rice. Appl. Microbiol. Biotechnol. 100, 5059–5067. 10.1007/s00253-016-7400-4 26948237 PMC4866983

[B265] Shin D. Choi W. Bae H. (2018). Bee venom phospholipase A2 alleviate house dust mite-induced atopic dermatitis-like skin lesions by the CD206 mannose receptor. Toxins 10 (4), 146. 10.3390/toxins10040146 29614845 PMC5923312

[B266] Shin S.-H. Ye M.-K. Choi S.-Y. Park K.-K. (2017). The effects of melittin and apamin on airborne fungi-induced chemical mediator and extracellular matrix production from nasal polyp fibroblasts. Toxins 9 (11), 348. 10.3390/toxins9110348 29076987 PMC5705963

[B267] Silva J. Monge-Fuentes V. Gomes F. Lopes K. Dos Anjos L. Campos G. (2015). Pharmacological alternatives for the treatment of neurodegenerative disorders: wasp and bee venoms and their components as new neuroactive tools. Toxins 7 (8), 3179–3209. 10.3390/toxins7083179 26295258 PMC4549745

[B268] Silva L. N. De Mello T. P. De Souza Ramos L. Branquinha M. H. Dos Santos A. L. S. (2019). Current challenges and updates on the therapy of fungal infections. Curr. Top. Med. Chem. 19 (7), 495–499. 10.2174/156802661907190531093808 31210103

[B269] Singh A. K. Malviya R. Prajapati B. Singh S. Yadav D. Kumar A. (2023). Nanotechnology-aided advancement in combating the cancer metastasis. Pharmaceuticals 16 (6), 899. 10.3390/ph16060899 37375846 PMC10304141

[B270] Smith B. D. Kaufman M. D. Lu W.-P. Gupta A. Leary C. B. Wise S. C. (2019). Ripretinib (DCC-2618) is a switch control kinase inhibitor of a broad spectrum of oncogenic and drug-resistant KIT and PDGFRA variants. Cancer Cell. 35 (5), 738–751. 10.1016/j.ccell.2019.04.006 31085175

[B271] Soares P. Silva C. Chavarria D. Silva F. S. G. Oliveira P. J. Borges F. (2023). Drug discovery and amyotrophic lateral sclerosis: emerging challenges and therapeutic opportunities. Ageing Res. Rev. 83, 101790. 10.1016/j.arr.2022.101790 36402404

[B272] Sobral F. Sampaio A. Falcão S. Queiroz M. J. R. P. Calhelha R. C. Vilas-Boas M. (2016). Chemical characterization, antioxidant, anti-inflammatory and cytotoxic properties of bee venom collected in Northeast Portugal. Food Chem. Toxicol. 94, 172–177. 10.1016/j.fct.2016.06.008 27288930

[B273] Soliman C. Eastwood S. Truong V. K. Ramsland P. A. Elbourne A. (2019). The membrane effects of melittin on gastric and colorectal cancer. PLoS One 14 (10), e0224028. 10.1371/journal.pone.0224028 31622415 PMC6797111

[B274] Somwongin S. Chantawannakul P. Chaiyana W. (2018). Antioxidant activity and irritation property of venoms from Apis species. Toxicon 145, 32–39. 10.1016/j.toxicon.2018.02.049 29499244

[B275] Son D. J. Lee J. W. Lee Y. H. Song H. S. Lee C. K. Hong J. T. (2007). Therapeutic application of anti-arthritis, pain-releasing, and anti-cancer effects of bee venom and its constituent compounds. Pharmacol. Ther. 115 (2), 246–270. 10.1016/j.pharmthera.2007.04.004 17555825

[B276] Stahelin R. V. (2016). Biochemistry of lipids, lipoproteins and membranes. Elsevier, 237–257.

[B277] Starkl P. Gaudenzio N. Marichal T. Reber L. L. Sibilano R. Watzenboeck M. L. (2022). IgE antibodies increase honeybee venom responsiveness and detoxification efficiency of mast cells. Allergy 77 (2), 499–512. 10.1111/all.14852 33840121 PMC8502784

[B278] Stepanova M. Clement S. Wong R. Saab S. Ahmed A. Younossi Z. M. (2017). Patients with diabetes and chronic liver disease are at increased risk for overall mortality: a population study from the United States. Clin. Diabetes 35 (2), 79–83. 10.2337/cd16-0018 28442821 PMC5391819

[B279] Sturm G. J. Varga E. M. Roberts G. Mosbech H. Bilò M. B. Akdis C. A. (2018). EAACI guidelines on allergen immunotherapy: Hymenoptera venom allergy. Allergy 73 (4), 744–764. 10.1111/all.13262 28748641

[B280] Suleiman J. B. Bakar A. B. A. Mohamed M. (2021). Review on bee products as potential protective and therapeutic agents in male reproductive impairment. Molecules 26 (11), 3421. 10.3390/molecules26113421 34198728 PMC8201164

[B281] Sung W.-S. Kim J.-H. Lee D. H. Kim E.-J. Seo B.-K. Hong S.-U. (2022). Effectiveness and safety of bee venom pharmacopuncture for rheumatoid arthritis: a systematic review protocol. BMJ open 12 (3), e056545. 10.1136/bmjopen-2021-056545 PMC8921846 35288390

[B282] Sur B. Lee B. Yeom M. Hong J.-H. Kwon S. Kim S.-T. (2015). Bee venom acupuncture alleviates trimellitic anhydride-induced atopic dermatitis-like skin lesions in mice. BMC complementary Altern. Med. 16, 38–13. 10.1186/s12906-016-1019-y PMC4731956 26825274

[B283] Światły-Błaszkiewicz A. Mrówczyńska L. Matuszewska E. Lubawy J. Urbański A. Kokot Z. J. (2020). The effect of bee venom peptides melittin, tertiapin, and apamin on the human erythrocytes ghosts: a preliminary study. Metabolites 10 (5), 191. 10.3390/metabo10050191 32413967 PMC7281017

[B284] Tanner C. M. Kamel F. Ross G. W. Hoppin J. A. Goldman S. M. Korell M. (2011). Rotenone, paraquat, and Parkinson’s disease. Environ. health Perspect. 119 (6), 866–872. 10.1289/ehp.1002839 21269927 PMC3114824

[B285] Tanuwidjaja I. Svečnjak L. Gugić D. Levanić M. Jurić S. Vinceković M. (2021). Chemical profiling and antimicrobial properties of honey bee (*Apis mellifera* L.) venom. Molecules 26 (10), 3049. 10.3390/molecules26103049 34065282 PMC8160683

[B286] Thawabteh A. Juma S. Bader M. Karaman D. Scrano L. Bufo S. A. (2019). The biological activity of natural alkaloids against herbivores, cancerous cells and pathogens. Toxins 11 (11), 656. 10.3390/toxins11110656 31717922 PMC6891610

[B287] Tiwari R. Tiwari G. Lahiri A. Ramachandran V. Rai A. (2022). Melittin: a natural peptide with expanded therapeutic applications. Nat. Prod. J. 12 (2), 13–29. 10.2174/2210315510999201210143035

[B288] Tsai L.-C. Lin Y.-W. Hsieh C.-L. (2015). Effects of bee venom injections at acupoints on neurologic dysfunction induced by thoracolumbar intervertebral disc disorders in canines: a randomized, controlled prospective study. BioMed Res. Int. 2015, 363801. 10.1155/2015/363801 26693480 PMC4676995

[B289] Tusiimire J. Wallace J. Woods N. Dufton M. J. Parkinson J. A. Abbott G. (2016). Effect of bee venom and its fractions on the release of pro-inflammatory cytokines in PMA-differentiated U937 cells co-stimulated with LPS. Vaccines 4 (2), 11. 10.3390/vaccines4020011 27104574 PMC4931628

[B290] Twarowski B. Herbet M. (2023). Inflammatory processes in alzheimer’s disease—pathomechanism, diagnosis and treatment: a review. Int. J. Mol. Sci. 24 (7), 6518. 10.3390/ijms24076518 37047492 PMC10095343

[B291] Uddin M. B. Lee B.-H. Nikapitiya C. Kim J.-H. Kim T.-H. Lee H.-C. (2016). Inhibitory effects of bee venom and its components against viruses *in vitro* and *in vivo* . J. Microbiol. 54, 853–866. 10.1007/s12275-016-6376-1 27888461 PMC7091203

[B292] Ullah A. Aldakheel F. M. Anjum S. I. Raza G. Khan S. A. Gajger I. T. (2023). Pharmacological properties and therapeutic potential of honey bee venom. Saudi Pharm. J. 31 (1), 96–109. 10.1016/j.jsps.2022.11.008 36685303 PMC9845117

[B293] Varol A. Sezen S. Evcimen D. Zarepour A. Ulus G. Zarrabi A. (2022). Cellular targets and molecular activity mechanisms of bee venom in cancer: recent trends and developments. Toxin Rev. 41 (4), 1382–1395. 10.1080/15569543.2021.2024576

[B294] Vickers N. J. (2017). Animal communication: when i’m calling you, will you answer too? Curr. Biol. 27 (14), R713–R715. 10.1016/j.cub.2017.05.064 28743020

[B295] Villa C. Suphesiz H. Combi R. Akyuz E. (2020). Potassium channels in the neuronal homeostasis and neurodegenerative pathways underlying Alzheimer’s disease: an update. Mech. ageing Dev. 185, 111197. 10.1016/j.mad.2019.111197 31862274

[B296] Virk M. S. Virk M. A. He Y. Tufail T. Gul M. Qayum A. (2024). The anti-inflammatory and curative exponent of probiotics: a comprehensive and authentic ingredient for the sustained functioning of major human organs. Nutrients 16 (4), 546. 10.3390/nu16040546 38398870 PMC10893534

[B297] Wachinger M. Kleinschmidt A. Winder D. Von Pechmann N. Ludvigsen A. Neumann M. (1998). Antimicrobial peptides melittin and cecropin inhibit replication of human immunodeficiency virus 1 by suppressing viral gene expression. J. General Virology 79 (4), 731–740. 10.1099/0022-1317-79-4-731 9568968

[B298] Wang Q. Qin X. Fang J. Sun X. (2021a). Nanomedicines for the treatment of rheumatoid arthritis: state of art and potential therapeutic strategies. Acta Pharm. Sin. B 11 (5), 1158–1174. 10.1016/j.apsb.2021.03.013 34094826 PMC8144894

[B299] Wang T. Zhang J. Xiao A. Liu W. Shang Y. An J. (2016). Melittin ameliorates CVB3-induced myocarditis via activation of the HDAC2-mediated GSK-3β/Nrf2/ARE signaling pathway. Biochem. biophysical Res. Commun. 480 (1), 126–131. 10.1016/j.bbrc.2016.09.135 27693786

[B300] Wang W. Zhou H. Liu L. (2018). Side effects of methotrexate therapy for rheumatoid arthritis: a systematic review. Eur. J. Med. Chem. 158, 502–516. 10.1016/j.ejmech.2018.09.027 30243154

[B301] Wang X.-M. Liu X.-M. Wang Y. Chen Z.-Y. (2021b). Activating transcription factor 3 (ATF3) regulates cell growth, apoptosis, invasion and collagen synthesis in keloid fibroblast through transforming growth factor beta (TGF-beta)/SMAD signaling pathway. Bioengineered 12 (1), 117–126. 10.1080/21655979.2020.1860491 33315500 PMC8806324

[B302] Wehbe R. Frangieh J. Rima M. El Obeid D. Sabatier J.-M. Fajloun Z. (2019). Bee venom: overview of main compounds and bioactivities for therapeutic interests. Molecules 24 (16), 2997. 10.3390/molecules24162997 31430861 PMC6720840

[B303] Xing L. Dawei C. Liping X. Rongqing Z. (2003). Oral colon-specific drug delivery for bee venom peptide: development of a coated calcium alginate gel beads-entrapped liposome. J. Control. Release 93 (3), 293–300. 10.1016/j.jconrel.2003.08.019 14644579

[B304] Xing X. Zhang X. Fan J. Zhang C. Zhang L. Duan R. (2024). Neuroprotective effects of melittin against cerebral ischemia and inflammatory injury via upregulation of MCPIP1 to suppress NF-κB activation *in vivo* and *in vitro* . Neurochem. Res. 49 (2), 348–362. 10.1007/s11064-023-04030-7 37812268 PMC10787673

[B305] Xu W. Zhou W. Wang H. Liang S. (2020). Roles of Porphyromonas gingivalis and its virulence factors in periodontitis. Adv. protein Chem. Struct. Biol. 120, 45–84. 10.1016/bs.apcsb.2019.12.001 32085888 PMC8204362

[B306] Yaacoub C. Wehbe R. Roufayel R. Fajloun Z. Coutard B. (2023). Bee venom and its two main components—melittin and phospholipase A2—as promising antiviral drug candidates. Pathogens 12 (11), 1354. 10.3390/pathogens12111354 38003818 PMC10674158

[B307] Yang E. J. Kim S. H. Yang S. C. Lee S. M. Choi S.-M. (2011). Melittin restores proteasome function in an animal model of ALS. J. neuroinflammation 8, 69–9. 10.1186/1742-2094-8-69 21682930 PMC3142224

[B308] Yang L. Zhao W. Gong X. Yue D. Liu Y. Tian Y. (2023). Exploring potential network pharmacology-and molecular docking-based mechanism of melittin in treating rheumatoid arthritis. Medicine 102 (32), e34728. 10.1097/MD.0000000000034728 37565866 PMC10419517

[B309] Ye M. Chung H.-S. Lee C. Hyun Song J. Shim I. Kim Y.-S. (2016a). Bee venom phospholipase A2 ameliorates motor dysfunction and modulates microglia activation in Parkinson’s disease alpha-synuclein transgenic mice. Exp. Mol. Med. 48 (7), e244. 10.1038/emm.2016.49 27388550 PMC4973312

[B310] Ye M. Chung H.-S. Lee C. Yoon M. S. Yu A. R. Kim J. S. (2016b). Neuroprotective effects of bee venom phospholipase A2 in the 3xTg AD mouse model of Alzheimer’s disease. J. neuroinflammation 13, 10–12. 10.1186/s12974-016-0476-z 26772975 PMC4715334

[B311] Yin D. Hsieh Y.-C. Tsai W.-C. Zhi-Yang Wu A. Jiang Z. Chan Y.-H. (2017). Role of apamin-sensitive calcium-activated small-conductance potassium currents on the mechanisms of ventricular fibrillation in pacing-induced failing rabbit hearts. Circulation Arrhythmia Electrophysiol. 10 (2), e004434. 10.1161/CIRCEP.116.004434 PMC5351779 28213506

[B312] Yook T.-H. Yu J.-S. Jung H.-S. (2008). Effects of sweet bee venom and bee venom on the heart rate variability. J. Pharmacopuncture 11 (1), 41–54. 10.3831/kpi.2008.11.1.041

[B313] Yoon J. Jeon J.-H. Lee Y.-W. Cho C.-K. Kwon K.-R. Shin J.-E. (2012). Sweet bee venom pharmacopuncture for chemotherapy-induced peripheral neuropathy. J. Acupunct. meridian Stud. 5 (4), 156–165. 10.1016/j.jams.2012.05.003 22898064

[B314] Yoon S.-Y. Yeo J.-H. Han S.-D. Bong D.-J. Oh B. Roh D.-H. (2013). Diluted bee venom injection reduces ipsilateral mechanical allodynia in oxaliplatin-induced neuropathic mice. Biol. Pharm. Bull. 36 (11), 1787–1793. 10.1248/bpb.b13-00469 23985901

[B315] Yoon Heera Y. H. Kim Minjoon K. M. Yoon Insoo Y. I. Li Dongxing L. D. Bae Hyunsu B. H. Kim Sunkwang K. S. (2015). Nicotinic acetylcholine receptors mediate the suppressive effect of an injection of diluted bee venom into the GV3 acupoint on oxaliplatin-induced neuropathic cold allodynia in rats. 10.1248/bpb.b14-00797 25752933

[B316] You C. E. Moon S. H. Lee K. H. Kim K. H. Park C. W. Seo S. J. (2016). Effects of emollient containing bee venom on atopic dermatitis: a double-blinded, randomized, base-controlled, multicenter study of 136 patients. Ann. dermatology 28 (5), 593–599. 10.5021/ad.2016.28.5.593 PMC5064189 27746639

[B317] Younossi Z. Henry L. (2016). Contribution of alcoholic and nonalcoholic fatty liver disease to the burden of liver-related morbidity and mortality. Gastroenterology 150 (8), 1778–1785. 10.1053/j.gastro.2016.03.005 26980624

[B318] Younossi Z. M. Wong G. Anstee Q. M. Henry L. (2023). The global burden of liver disease. Clinical Gastroenterology and Hepatology. 10.1016/j.cgh.2023.04.015 37121527

[B319] Yu J. Shen Q. Li J. (2024). Toxicology study profile of Nicotinamide mononucleotide after acute and 90-day sub chronic dosing in Wistar rats and mutagenicity tests. Curr. Res. Toxicol., 100171. 10.1016/j.crtox.2024.100171 38765763 PMC11101926

[B320] Yu X. Jia S. Yu S. Chen Y. Zhang C. Chen H. (2023). Recent advances in melittin-based nanoparticles for antitumor treatment: from mechanisms to targeted delivery strategies. J. nanobiotechnology 21 (1), 454. 10.1186/s12951-023-02223-4 38017537 PMC10685715

[B321] Zarei S. Carr K. Reiley L. Diaz K. Guerra O. Altamirano P. F. (2015). A comprehensive review of amyotrophic lateral sclerosis. Surg. Neurol. Int. 6, 171. 10.4103/2152-7806.169561 26629397 PMC4653353

[B322] Zarrinnahad H. Mahmoodzadeh A. Hamidi M. P. Mahdavi M. Moradi A. Bagheri K. P. (2018). Apoptotic effect of melittin purified from Iranian honey bee venom on human cervical cancer HeLa cell line. Int. J. peptide Res. Ther. 24, 563–570. 10.1007/s10989-017-9641-1 30416405 PMC6208649

[B323] Zein N. Yassin F. Eldeen E. Elshorbagy I. (2024). The effect of Bee venom as anti-inflammatory, antioxidant and antitumor agent in mice with hepatocellular carcinoma. Biochem. Lett. 20 (1), 12–25. 10.21608/blj.2024.340261

[B324] Zhang H.-Q. Sun C. Xu N. Liu W. (2024). The current landscape of the antimicrobial peptide melittin and its therapeutic potential. Front. Immunol. 15, 1326033. 10.3389/fimmu.2024.1326033 38318188 PMC10838977

[B325] Zhang J. Gold K. A. Lin H. Y. Swisher S. G. Xing Y. Lee J. J. (2015). Relationship between tumor size and survival in non–small-cell lung cancer (NSCLC): an analysis of the surveillance, epidemiology, and end results (SEER) registry. J. Thorac. Oncol. 10 (4), 682–690. 10.1097/JTO.0000000000000456 25590605 PMC4368494

[B326] Zhang S. F. Chen Z. (2017). Melittin exerts an antitumor effect on non-small cell lung cancer cells. Mol. Med. Rep. 16 (3), 3581–3586. 10.3892/mmr.2017.6970 28713976

[B327] Zhao J. Hu W. Zhang Z. Zhou Z. Duan J. Dong Z. (2022). Bee venom protects against pancreatic cancer via inducing cell cycle arrest and apoptosis with suppression of cell migration. J. Gastrointest. Oncol. 13 (2), 847–858. 10.21037/jgo-22-222 35557571 PMC9086033

[B328] Zhao R. Xue M. Lin H. Smith M. Liang H. Weiler H. (2024). A recombinant signalling‐selective activated protein C that lacks anticoagulant activity is efficacious and safe in cutaneous wound preclinical models. Wound Repair Regen. 32 (1), 90–103. 10.1111/wrr.13148 38155595

[B329] Zhou Y. Tang J. Lan J. Zhang Y. Wang H. Chen Q. (2023). Honokiol alleviated neurodegeneration by reducing oxidative stress and improving mitochondrial function in mutant SOD1 cellular and mouse models of amyotrophic lateral sclerosis. Acta Pharm. Sin. B 13 (2), 577–597. 10.1016/j.apsb.2022.07.019 36873166 PMC9979194

[B330] Zuazo-Gaztelu I. Casanovas O. (2018). Unraveling the role of angiogenesis in cancer ecosystems. Front. Oncol. 8, 248. 10.3389/fonc.2018.00248 30013950 PMC6036108

